# Assessing the capabilities of AI-based large language models (AI-LLMs) in interpreting histopathological slides and scientific figures: Performance evaluation study

**DOI:** 10.37796/2211-8039.1698

**Published:** 2026-03-01

**Authors:** Khanisyah E. Gumilar, Grace Ariani, Priangga A. Wiratama, Rimbun, Tri H. Yuliawati, Hong Chen, Ibrahim H. Ibrahim, Cheng-Han Lin, Tai-Yu Hung, Dewanti Anggrahini, Arya S. Rajanagara, Khaled E. Omran, Zih-Ying Yu, Yu-Cheng Hsu, Erry G. Dachlan, Jer-Yen Yang, Li-Na Liao, Ming Tan

**Affiliations:** a Graduate Institute of Biomedical Science, China Medical University, Taichung, Taiwan, ROC; b Department of Obstetrics and Gynecology, Universitas Airlangga Hospital - Faculty of Medicine, Universitas Airlangga, Surabaya, Indonesia; c Department of Pathology Anatomy, Faculty of Medicine, Universitas Airlangga, Surabaya, Indonesia; d Department of Anatomy, Histology and Pharmacology, Faculty of Medicine, Universitas Airlangga, Surabaya, Indonesia; e Department of Industrial Engineering and Engineering Management, National Tsing Hua University, Hsinchu, Taiwan, ROC; f Department of Industrial & Systems Engineering, Institut Teknologi Sepuluh Nopember, Surabaya, Indonesia; g Department of Public Health, China Medical University, Taichung, Taiwan, ROC; h School of Chinese Medicine, China Medical University, Taichung, Taiwan, ROC; i Institute of Biochemistry and Molecular Biology and Research Center for Cancer Biology, China Medical University, Taichung, Taiwan, ROC

**Keywords:** AI-LLMs, Artificial intelligence, Large language model, Histopathological image, Scientific figure, ChatGPT, Gemini, Copilot, Medical diagnostics

## Abstract

**Background:**

Integrating artificial intelligence-based large language models (AI-LLMs) into medical and other scientific domains is increasingly recognized as a tool to support complex tasks, such as interpreting histopathology slides and scientific figures. AI-LLMs can simplify these processes by providing clearer explanations. By improving accessibility and comprehension, AI-LLMs can significantly assist healthcare professionals in diagnosing and therapy determination. Students and the public also find it easier to understand complex scientific concepts and images.

**Objectives:**

This study explores the capability of AI-LLMs in interpreting histopathological slides and scientific images. This study aims to evaluate the performance of AI-LLMs in supporting diagnostics and improving comprehension in biomolecular sciences.

**Methods:**

The study was divided into two parts: interpreting histopathology slides and scientific figures. Twelve histopathology images and twelve scientific figures were tested on each of the three most frequently used chatbots (ChatGPT-4, Gemini Advanced, and Copilot). Responses from the chatbots were coded and blindly examined by expert raters using five parameters—relevance, clarity, depth, focus, and coherence—on a 5-point Likert scale. Statistical analysis included one-way ANOVA and multiple linear regression.

**Results:**

ChatGPT-4 outperformed Gemini Advanced and Copilot in histopathology and scientific image interpretation (P < 0.001) with significantly higher scores across all parameters (relevance, clarity, depth, focus, and coherence). ChatGPT-4’s superior performance may be due to its advanced algorithms, extensive training data, specialized modules, and user feedback.

**Conclusions:**

ChatGPT-4 excels in interpreting histopathology and scientific images, which may lead to improving diagnostic accuracy, clinical decision-making, and reducing pathologists’ workload. It also benefits education by enhancing students’ understanding of complex images and promoting interactive learning. ChatGPT-4 shows a significant potential to improve patient care and enrich student learning.

## 1. Introduction

The integration of artificial intelligence (AI) technology into the daily routines and professional workflows of oncologists is steadily increasing. Effectively incorporating AI into clinical practice requires understanding the development, validation, and continuous improvement of these technologies.

Interpreting histopathology slides is a complex process that requires specialized skills and indepth experience. In this process, the pathologist must be able to identify and analyze the different types of tissues and cells present in the slide. Each tissue and cell type has unique morphological characteristics, requiring an in-depth understanding of anatomy and pathology [1,2]. Along the same lines, interpreting scientific figures published in scientific journals or textbooks can also be challenging for students and laypeople. These figures often contain highly technical and complex information, such as cellular biological mechanisms, mechanisms of tumor development, or cell death pathways, which require a deep understanding of the relevant disciplines [3]. This difficulty is compounded by using specialized terminology and unfamiliar symbols, which can confuse and hinder proper understanding. Without an academic background or experience in these fields, students and laypeople may struggle to understand the context and meaning of these images.

To overcome these challenges, AI-LLMs can offer significant assistance. With the ability to understand and analyze language and images, AI-LLMs can provide simpler and more accessible explanations of complex scientific figures. AI can identify key elements in an image, translate them into easy-to-understand language, and provide an explanation [4,5]. Moreover, AI-LLMs can offer interactive assistance, where users can ask specific questions and receive relevant answers and feedback, enhancing understanding and making scientific information more accessible to students and the public.

In this study, we investigate ChatGPT4, Gemini Advanced, and Copilot’s ability to interpret histopathology slides and illustrative scientific figures. It discusses the potential of AI LLM’s impact in enhancing the accessibility of medical and scientific material to a wider audience. As the amount of visual data used in medical diagnostics and scientific research grows, the capacity to efficiently understand and transmit this information becomes increasingly important. We anticipate that our research will demonstrate how AI-LLMs technology can be a valuable tool in assisting specialists in the medical and scientific fields, as well as individuals without specialized backgrounds, to interpret complex material. This study may help bridge the knowledge gap and open new opportunities for using AI in different research fields.

## 2. Methods

### 2.1. Ethics

In accordance with local legislation and institutional requirements, ethical review and approval were not required for the study of human participants.

### 2.2. Materials

We used three AI-based chatbots in this study: ChatGPT-4 (https://chatgpt.com/), Gemini Advanced (https://gemini.google.com/app), and Copilot (https://www.bing.com/).

### 2.3. Study design

**Fig. 1:**
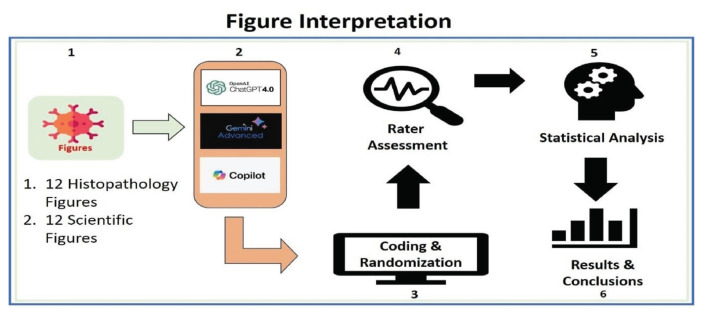
Workflow of image interpretation by chatbots. 12 histopathology and 12 scientific images were tested on each chatbot (CG -4, GemAdv, and Copilot). The interpretation of each chatbot will be coded and randomized to avoid bias. Raters will evaluate all answers, and then statistical analysis will be done.

The study was divided into two parts: 1. Interpretation of histopathology slides and 2. Interpretation of scientific figures. We tested three AI-LLMs (hereinafter referred to as chatbots): ChatGPT-4 (hereinafter referred to as CG-4), Gemini Advanced (hereinafter referred to as GemAdv), and Copilot with various figures (Fig. 1).

**Fig. 2:**
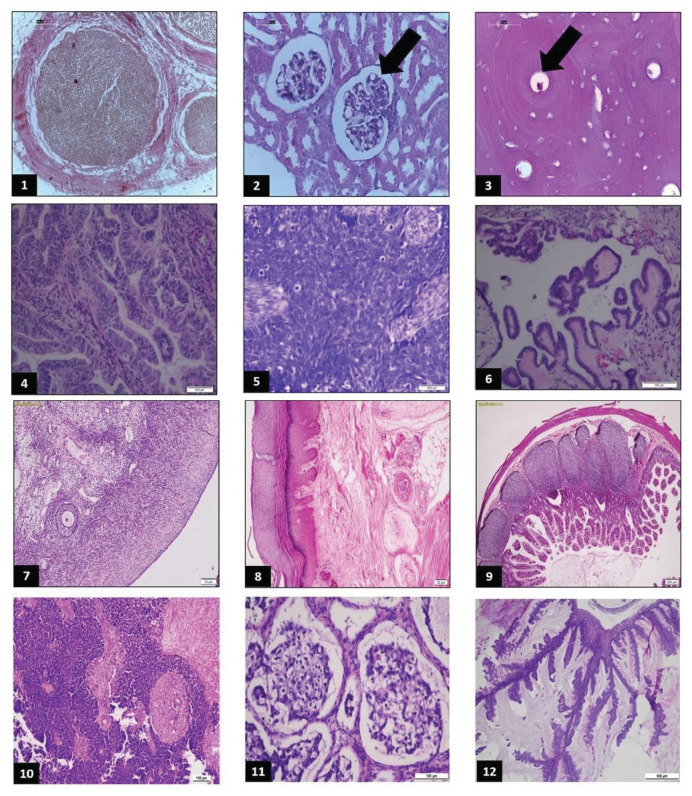
**Histopathology Slides**, consisting of 1) normal peripheral nerves; 2) normal kidney; 3) normal bone tissue; 4) ovarian cancer, endometrioid grade-1; 5) ovarian cancer, endometrioid grade-3; 6) serous borderline ovarian tumor with implantation; 7) normal ovarium; 8) Thick skin from palmar side; 9) normal ileum; 10) cervical cancer, small cell carcinoma; 11) ovarian cancer, clear cell carcinoma type; 12) ovarian cancer, mucinous type.

In the first part, we will provide 12 histopathology slides consisting of six normal tissue images and six neoplastic tissue images (Fig. 2). Each image will be given a code prompt, and then tested on each chatbot (Supp. 1 (https://www.biomedicinej.com/cgi/editor.cgi?article=1698&window=additional_files&context=biomedicine)). To address potential biases arising from these issues, we enlisted the expertise of four highly experienced histopathologists to carefully select a diverse range of histological slides. These included normal tissue slides from various organs and gynecologic cancer slides, which were specifically chosen to align with the experts’ specialized knowledge and practical experience. This selection process ensured that the dataset used in the study was both representative and relevant, providing a robust foundation for evaluating the performance of the chatbots. By incorporating expert input, we aimed to minimize bias and enhance the reliability of the study’s outcomes.

**Fig. 3:**
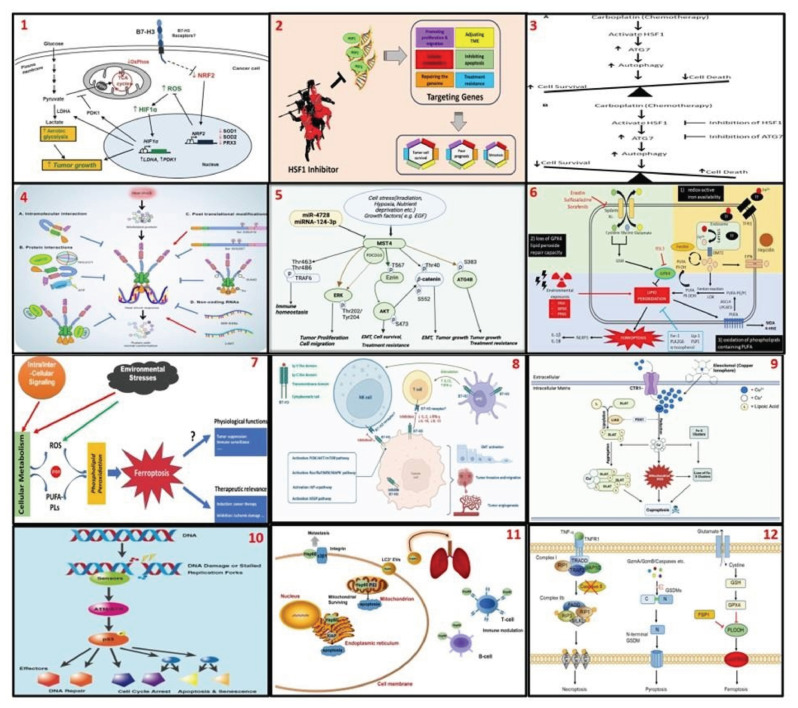
Illustrative scientific figures consisting of 1) The role of B7H3 regulating glucose metabolism; 2) The role of HSF1 in tumor cell survival, poor prognosis, and metastasis through several mechanisms; 3) Carboplatin, HSF1, and autophagy; 4) The heat shock response and the regulator of HSF1; 5) Mechanisms of MST4-induced tumor progression and treatment resistance; 6) Three hallmarks of ferroptosis; 7) An overview of ferroptosis; 8) B7-H3 non-immune-mediated and immune-mediated signaling pathways; 9) Mechanisms of copper-induced cell death; 10) Schematic representation of p53-mediated DNA damage response; 11) A model illustrating Hsp60’s regulation of breast cancer; and 12) Key non-apoptotic regulated cell death pathways.

In the second part, we provided 12 scientific figures (Table 1) (Fig. 3). Each image was assigned a code prompt and examined with each chatbot (Supp. 2 (https://www.biomedicinej.com/cgi/editor.cgi?article=1698&window=additional_files&context=biomedicine)). The selection of images in this section is based on the expertise and professional experience of the researchers in our laboratory, which focuses on oncology, immunology, and mechanisms of cell death.

The interpretations were promptly recorded in a database, coded, and subjected to blinded review by a team of raters (four histopathology experts in part 1 and four biomedical scientists in part 2). To eliminate bias, the AI chatbot responses were coded and randomized before being scored by the raters. The raters evaluated the responses without knowing which came from the chatbot. To analyze the output of the chatbots, we used 5 parameters, including “relevance”, “clarity”, “depth”, “focus”, and “coherence” [18–21] with a 5-point Likert scale (Table 2) [22–25].

### 2.4. Statistical analysis

We investigated the performance of three chatbots in interpreting images. For the evaluation of five parameters, the scores for the five parameters were categorized as follows: 1–2, 3, and 4–5 were classified as “poor,” “fair,” and “good,” respectively [25]. To enhance the interpretability of responses [26–29], the 5-point Likert scale ratings were linearly converted to a 0–100 scale, with higher scores representing superior performance. To assess the consistency among different raters, we reported Pearson and Spearman correlation coefficients for all ratings and employed a one-way ANOVA test with Scheffe’s post hoc analysis to examine differences in total scores across raters. The presence of high inter-rater consistency enhances the generalizability of the evaluation results. To compare the interpretative abilities of three chatbots regarding histopathology images or scientific illustrations, a one-way ANOVA test with Scheffe’s post hoc analysis was employed. Furthermore, to reduce the confounding impact of evaluator subjectivity and the complexity of images, multiple linear regression models were utilized. All statistical analyses were performed using the SAS software (Version 9.4, SAS Institute, Cary, NC, USA), with a significance level set at 0.05.

## 3. Results

### 3.1. CG-4 surpasses GemAdv and Copilot in providing histopathology image interpretation

**Fig. 4:**
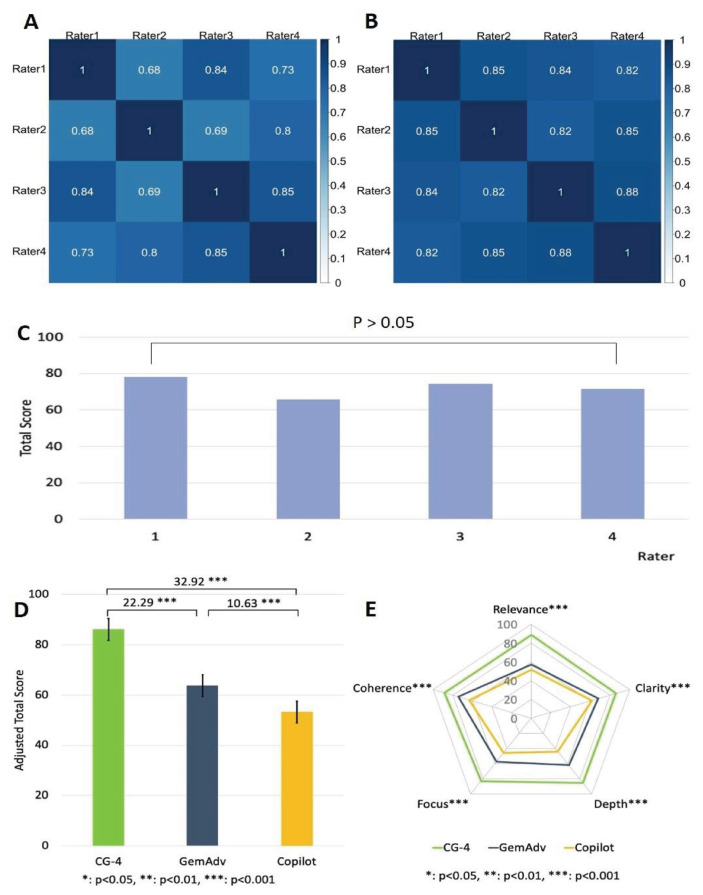
CG-4 surpasses GemAdv and Copilot in providing histopathology image interpretation. Pearson test (**A**) and Spearman test (**B**) showed high homogeneity among raters, respectively 0.68–0.85 & 0.82–0.88. One-way ANOVA test with Scheffe’s post hoc analysis showed no significant differences among the raters in giving assessments (**C**). CG4 scores significantly better than the other two AI-LLMs in providing histopathology image interpretation (**D**). The 5 parameters used for interpretation quality assessment show CG4’s superiority significantly (**E**).

Recently, advanced software tools and platforms in digital pathology have emerged to assist pathologists in analyzing histopathological images and making diagnoses [30]. However, not all pathologists, especially those in less developed countries/regions, have access to these smart pathology AI technologies. Therefore, it is crucial to evaluate the value of publicly accessible AI chatbots for interpreting histopathology images. To evaluate the capacities of the chatbots for histopathology image interpretation, 12 histopathology images were tested on each chatbot. The image interpretations by the chatbots were coded and blindly evaluated by four board-certified pathologists (raters). To validate our results, we used three statistical methods to analyze the homogeneity of the raters’ rating scores. We found that most raters showed correlation coefficients between 0.68 and 0.85 by the Pearson test (Fig. 4A), and 0.820.88 by the Spearman test (Fig. 4B). Furthermore, a one-way ANOVA test with Scheffe’s post hoc analysis also showed no significant variation either (Fig. 4C). All these results indicate that the raters’ scores are highly homogenous, indicating that the scoring process is reliable. To assess the quality of the image interpretations by the chatbots, we analyzed the scores of the chatbots in the five individual parameters. Overall, among the three chatbots, CG-4 scored higher than GemAdv and Copilot by a significant (*P* < 0.001) (Fig. 4D), suggesting that CG-4 provides better interpretations of histopathology images. In addition, CG-4 also showed convincing superiority in all 5 parameters (*P* < 0.001) (Fig. 4E).

### 3.2. CG-4 can provide superior scientific figure interpretation than GemAdv or Copilot

**Fig. 5:**
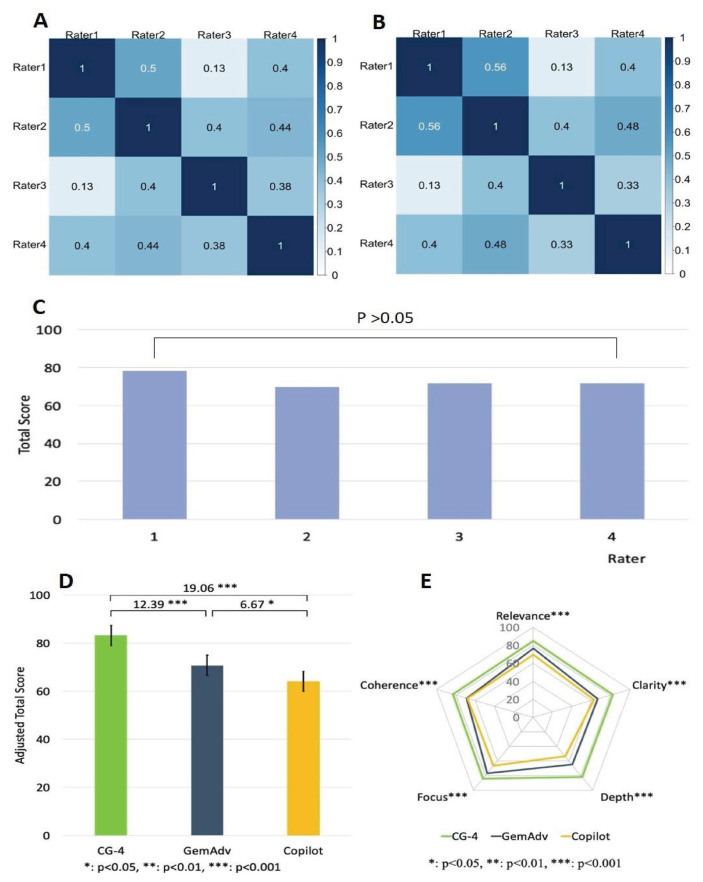
G-4 can provide superior scientific figure interpretation. Pearson test **(A)** and Spearman test **(B)** showed homogeneity among raters, respectively 0.13–0.5 & 0.13–0.56. One-way ANOVA test with Scheffe’s post hoc analysis showed no significant differences among the raters in giving assessments **(C)**. CG-4 scores significantly better than the other two AI-LLMs in providing scientific image interpretation **(D)**. CG-4 convincingly outperformed in 5 assessment parameters with varying significance. Depth and Focus (P < 0 (0.001); Clarity and Coherence P < 0.01); and relevance (P < 0.05), respectively **(E)**.

Understanding the figures in scientific papers and textbooks is essential for grasping the key points in the articles, so it is crucial to comprehend them. Next, we tested 12 scientific figures from scientific journals on the 3 chatbots (CG-4, GemAdv, and Copilot). The image interpretations by the chatbots were coded and blindly evaluated by four researchers with intensive training in the fields that are related to scientific figures. To validate our results, we analyzed the rater’s scoring homogeneity as aforementioned. We found that most raters showed correlation coefficients between 0.13 and 0.5 using the Pearson test (Fig. 5A), and 0.130.56 using the Spearman test (Fig. 5B). Moreover, we used a one-way ANOVA test with Scheffe’s post hoc analysis to assess the raters’ scoring homogeneity (Fig. 5C). The analyses show that there are no significant statistical variations among these raters, which supports the reliability of our results.

To determine the chatbot that provided the most effective responses, we analyzed the scores of three different chatbots across five parameters. Our findings revealed that CG-4 achieved a significantly higher score than both GemAdv and Copilot (Fig. 5D) by a substantial margin (P < 0.001). Notably, CG-4 demonstrated superior performance in every aspect (P < 0.001) (Fig. 5E). This indicates the significant superiority of CG-4 compared to the other two chatbots in providing interpretations of complex and intricate scientific images.

## 4. Discussion

This study identified significant differences in the ability of the three chatbots to interpret histopathology and scientific figures. CG-4 outperformed GemAdv and Copilot in the first part of the study, which focused on the interpretation of histopathological images. This superiority was evident in the overall total scores as well as in the five evaluation parameters (relevance, clarity, depth, focus, and coherence). This outstanding performance may be attributed to CG4’s more advanced and experienced model in image analysis compared to the other two AILLMs [31,32]. This conclusion is further supported by a high level of evaluator consensus (Fig. 4A—C), indicating strong agreement among experts in their assessments.

In the second part of the study, although the homogeneity index among evaluators in this section was lower (Fig. 5A & B) compared to the evaluators of histopathological images, no significant differences were observed between them (Fig. 5C). The use of three different statistical tests provided an effective and reliable approach to assess the consistency of evaluators’ perceptions in their assessments. Thus, the evaluations provided by the experts serve as a critical foundation for conclusions.

CG-4 outperformed the other chatbots in interpreting scientific images. Its performance demonstrated significant superiority across all parameters, indicating that CG-4 possesses a better capability to comprehend and convey complex information from scientific images compared to its competitors.

Possible factors causing the differences in results among the three AI-LLMs include: A) Algorithm and Model Architecture [33]: CG-4 may have employed more sophisticated algorithms and model architectures trained on more diverse and specific data, leading to more accurate and in-depth interpretations. B) Training Data [34,35]: CG-4 may be trained with larger and more diverse datasets, including histopathology data and scientific images, giving the model a better understanding of different types of images and their context. C) Focus on Medical and Scientific [36]: CG-4 may have components or modules specifically developed for medical and scientific applications, allowing the model to provide more focused and relevant interpretations in these domains. D) User Experience [37]: The wider use of CG-4 and more user feedback may also contribute to the improved performance of this model compared to GemAdv and Copilot.

The main challenges in histopathological slide interpretation include variability in assessment among pathologists, slide quality, morphological similarities between entities, and tissue heterogeneity. To mitigate biases arising from these issues, we engaged four histopathology experts to select normal slides from various organs and gynecologic cancer slides, areas that align with our expertise and practice. The prompts and clinical scenarios we posed also played a significant role in guiding the chatbot’s responses. For instance, adding phrases like *“What is the structure pointed to by the black arrow in the image?”* (Sup.1 Q3 & Q4 (https://www.biomedicinej.com/cgi/editor.cgi?article=1698&window=additional_files&context=biomedicine)) or *“What grade category does this slide belong to?”* (Sup.1 Q5 (https://www.biomedicinej.com/cgi/editor.cgi?article=1698&window=additional_files&context=biomedicine)) directed and facilitated satisfactory chatbot responses. Clinical scenarios that included initial diagnoses and procedures performed for all cancer cases (Sup.1 Q4–6 & Q10–12 (https://www.biomedicinej.com/cgi/editor.cgi?article=1698&window=additional_files&context=biomedicine)) also served as critical contextual elements that helped the chatbot avoid bias when providing interpretations. Lastly, we tested rare and specific samples (Sup.1 Q6, Q11 & Q12 (https://www.biomedicinej.com/cgi/editor.cgi?article=1698&window=additional_files&context=biomedicine)).

The participation of histopathology experts and researchers as raters in this study had a positive impact and added objectivity to the findings. Both groups of raters demonstrated no significant differences in their assessments (Figs. 4C & 5C), indicating a shared perception of the interpretations provided by the chatbots. Furthermore, we employed five standardized evaluation parameters to assess the chatbots’ responses. Our previous research has shown that this approach comprehensively evaluates the accuracy and correctness of the responses [24].

For the evaluation of scientific images, the prompts we used consistently began with the title of the image (as stated in the reference title) and concluded with the command, *“Please give a suitable interpretation of this picture,”* across all chatbots. This structured prompt approach ensured that the chatbot provided accurate and expected interpretations. Considering the potential of AI-LLMs to fabricate [38], hallucinate [39], and generate misinformation [40], the structured command methodology implemented in this study was a crucial element in ensuring reliability and consistency.

## 5. Implications of AI-LLMs for patient care

The incorporation of AI into professional healthcare is progressively advancing. A thorough understanding of the methodologies underlying the design, validation, and ongoing optimization of these technologies is essential for their effective integration into clinical practice. Our findings indicate that CG-4 has strong abilities in interpreting histopathology images, which could lead to important benefits for patient care. First, CG-4 can help pathologists make more accurate diagnoses by better identifying and analyzing tissue and cell structures on histopathology slides.

For instance, all raters were impressed by the accuracy of the LLM in identifying grade-3 in ovarian cancer specimens (Supp.1 Bot-1 & Bot-2 on Q5 (https://www.biomedicinej.com/cgi/editor.cgi?article=1698&window=additional_files&context=biomedicine)), recognizing “tumor borderline malignancy” (Supp.1 Bot-1 on Q6 (https://www.biomedicinej.com/cgi/editor.cgi?article=1698&window=additional_files&context=biomedicine)), small cell carcinoma (Supp.1 Bot-1 on Q10 (https://www.biomedicinej.com/cgi/editor.cgi?article=1698&window=additional_files&context=biomedicine)), clear cell carcinoma (Supp.1 Bot-1 on Q11 (https://www.biomedicinej.com/cgi/editor.cgi?article=1698&window=additional_files&context=biomedicine)), and mucinous carcinoma (Supp.1 Bot-1 on Q11 (https://www.biomedicinej.com/cgi/editor.cgi?article=1698&window=additional_files&context=biomedicine)). This inferential ability greatly helps the pathologist in making accurate diagnoses, thereby enabling precise therapeutic interventions for patients. This can simultaneously lower the possibility of misdiagnosis and avoid patient morbidity and mortality. Additionally, CG-4’s ability to provide quick and accurate interpretations shortens the time needed for diagnosis, which is especially valuable when fast decisions are needed. The results of our experiment further reinforce the necessity of AI involvement in patient services to achieve better diagnostic quality.

In line with this, pathologists have shown significant interest in integrating AI into digital health systems as an innovation in the medical field [41]. The utilization of digital pathology also demonstrates superior performance in diagnostic and enhanced assessment [42–45]. Another study demonstrates the capability of AI to provide satisfactory responses in different branches of medicine and healthcare [46–50]. This remarkable capability is also useful for detecting out of-distribution data, or data that does not align with the distribution of training data [51]. The benefits and potential of this technology should serve as tools to assist professionals [52], although the risks of bias and ethical concerns remain challenges that must be anticipated [53]. The role of humans as validators and supervisors should remain a priority in addressing these issues [54].

Finally, the use of AI-LLMs can reduce the workload of pathologists, allowing them to concentrate on more complex cases that require greater human intervention. Furthermore, CG-4 can improve diagnostic accessibility in remote areas with a shortage of specialist pathologists. In such areas, CG-4 can be an invaluable tool, assisting local medical personnel in making initial diagnoses and offering treatment recommendations.

### 5.1. Implications of AI-LLMs on student learning of complex scientific figures

The study indicates that CG-4 excels in interpreting scientific figures compared to the other two AI-LLMs, which has several important implications for student learning. CG-4 can enhance students’ understanding of complex scientific images by providing clearer, more relevant, and in-depth explanations, thereby facilitating the learning process, and improving their comprehension of the material.

Although the tested images appeared complex, with variations in color, shape, and intricate symbols, all models provided satisfactory interpretations. All raters agreed that the chatbots’ ability to deliver systematic and coherent explanations was commendable. However, CG-4 outperformed the other two chatbots across all parameters.

The raters were also impressed by the chatbot’s ability to interpret up-to-date information. For instance, explanations regarding cuproptosis, a phenomenon discovered in 2022, were presented clearly and accurately (Supp.2 Q9 (https://www.biomedicinej.com/cgi/editor.cgi?article=1698&window=additional_files&context=biomedicine)). The chatbots also demonstrated the capacity to distinguish mechanisms of cell death (Supp.2 Q12 (https://www.biomedicinej.com/cgi/editor.cgi?article=1698&window=additional_files&context=biomedicine)) without including irrelevant details unrelated to the images. Overall, all chatbots exhibited strong performance and hold potential for further improvement in the future.

Additionally, CG-4 promotes learning interactivity, allowing students to engage directly with the AI, ask specific questions, and receive relevant answers, which supports their active and independent learning efforts.

CG-4 also contributes to the development of analytical skills among students by offering opportunities for data analysis exercises and immediate feedback. Students can independently analyze scientific figure data and then verify their results with CG-4, thereby honing their critical analytical skills in the scientific field. The AI provides immediate feedback, helping students identify strengths and weaknesses in their interpretations and guiding them toward skill improvement. Additionally, CG-4 broadens access to learning resources by providing explanations and interpretations of scientific images from various sources that might have been previously inaccessible. This enhances opportunities for broader and deeper learning. Lecturers and researchers can also utilize CG-4 as a teaching and research tool, offering additional explanations that students or research participants may require.

### 5.2. Limitations of the study

Although AI-LLMs demonstrate clear potential as educational tools for doctors and medical students, their application in diagnostic support remains in its early stages. The use of histopathology images in this study served as a simplified approach that does not fully capture the complexity of whole slide images (WSIs) encountered in routine pathology practice.

Further research is needed to evaluate the performance of chatbots in real-world clinical diagnostic settings.

## 6. Conclusion

This study highlights significant differences in the performance of three AI-LLMs (CG-4, GemAdv, and Copilot) in interpreting histopathological slides and scientific figures, with CG-4 demonstrating superior capabilities. CG-4 consistently outperformed its counterparts across evaluation parameters such as relevance, clarity, depth, focus, and coherence, particularly in histopathological image analysis. The chatbot’s advanced algorithms, diverse training datasets, and specific design for medical and scientific applications contributed to its strong performance. Furthermore, structured prompts and clinical scenarios ensured accurate responses, reducing bias and enhancing reliability in interpretations.

The findings underscore the potential of AI-LLMs like CG-4 to transform patient care by improving diagnostic accuracy, reducing pathologists’ workloads, and enhancing accessibility to diagnostic services in underserved regions. CG-4’s ability to identify key histopathological features and provide precise interpretations supports more accurate diagnoses and timely therapeutic interventions, ultimately improving patient outcomes. The study also demonstrates the potential for AI to complement human expertise, offering critical support in areas requiring rapid or complex decision-making.

In the context of education, CG-4 exhibited a significant capacity to enhance learning by offering detailed and systematic explanations of complex scientific figures. The chatbot’s ability to facilitate interactive learning, provide immediate feedback, and broaden access to educational resources makes it a valuable tool for students and educators. By fostering analytical and critical thinking skills, CG-4 contributes to a more effective and engaging learning environment, underscoring its potential as an educational aid in the scientific and medical fields.

## Supplementary Information





## Figures and Tables

**Table 1:** List of scientific figures.

Number	Title	Reference
1	The role of B7H3 in regulating glucose metabolism	[6]
2	The role of HSF1 in tumor cell survival, poor prognosis, and metastasis through several mechanisms	[7]
3	Carboplatin, HSF1, and autophagy	[8]
4	The heat shock response and the regulator of HSF1	[9]
5	Mechanisms of MST4-induced tumor progression and treatment resistance	[10]
6	Three hallmarks of ferroptosis	[11]
7	An overview of ferroptosis	[12]
8	B7-H3 non-immune-mediated and immune-mediated signaling pathways	[13]
9	Mechanisms of copper-induced cell death	[14]
10	Schematic representation of p53-mediated DNA damage response	[15]
11	A model illustrating Hsp60’s regulation of breast cancer	[16]
12	Key non-apoptotic regulated cell death pathways	[17]

**Table 2:** Assessment parameters and scoring.

Assessment Parameters	Definition
Relevance	The response is closely related or appropriate to the issue at hand
Clarity	Clear, easy to understand, free from ambiguity, and transparent
Depth	The answer provides detailed and specific information, not just a general or surface answer
Focus	Contains the main points or keywords expected
Coherence	All parts of the answer work together in a logical and structured way, with no conflicting parts

**Scoring Scale**	**Definitions**

1 = Very poor	The answer does not fulfil the basic criteria, is highly irrelevant, or shows no evident effort
2 = Poor	The answer fulfils very few of the expected criteria, with many basic errors
3 = Average	The answer fulfils the basic criteria but does not show more effort or understanding than expected
4 = Good	The answer fulfils all the basic criteria well and shows some aspects that are more than expected
5 = Outstanding	A perfect answer of flawless quality, showing exceptional understanding and complete mastery of the material
