# Supplementary material for: Assessing the capabilities of AI-based large language models (AI-LLMs) in interpreting histopathological slides and scientific figures: Performance evaluation study
Source: Biomedicine (Taipei). 2026 Mar 1;16(1):41–52. doi: 10.37796/2211-8039.1698 (PMC12962759; doi:10.37796/2211-8039.1698)
Supplement: Supplementary file 1 [file bmed-16-01-041-g001.pdf]

Supplementary 2 - Scientific figures

Question	Title
Q1	The role of B7H3 in regulating glucose metabolism
Q2	HSF1 plays an important role in tumor cell survival, poor prognosis, and metastasis through several mechanisms
Q3	Carboplatin, HSF1 and Autophagy
Q4	The heat shock response and the regulator of HSF1
Q5	Mechanisms of MST4-induced Tumor Progression and Treatment Resistance
Q6	Three hallmarks of ferroptosis
Q7	An Overview of Ferroptosis
Q8	B7-H3 non-immune-mediated and immune-mediated signaling pathways
Q9	Mechanisms of copper-induced cell death
Q10	Schematic representation of p53-mediated DNA Damage Response
Q11	A model illustrating Hsp60's regulation of breast cancer
Q12	Key non-apoptotic regulated cell death pathways

BOT-1 : ChatGPT-4
BOT-2 : Gemini Advanced
BOT-3 : Copilot

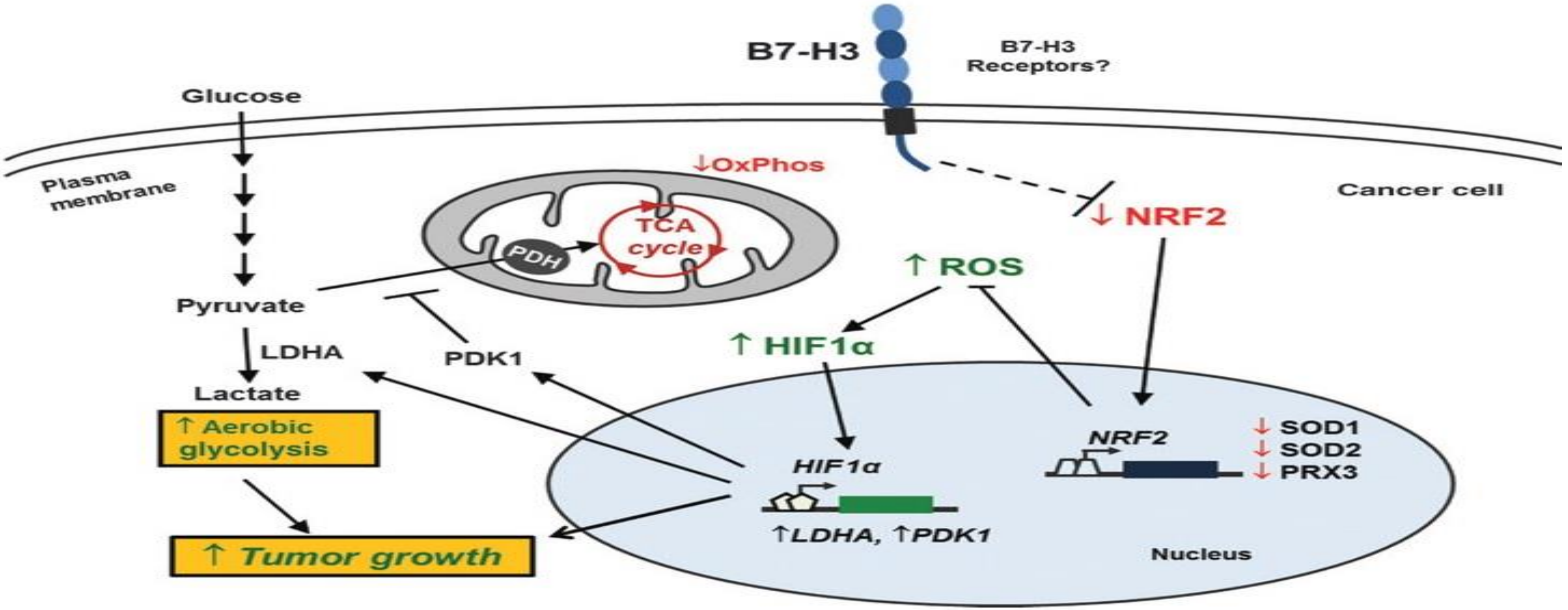

(Explanation from the original article)
Model for the role of B7-H3 regulating glucose metabolism. Through unknown mechanisms, B7-H3 suppresses Nrf2 transcriptional activity, which in turn reduces transcription of the antioxidant enzymes SOD1, SOD2, and PRX3. As a result, B7-H3 overexpression leads to increased ROS in cancer cells. B7-H3–induced ROS stabilizes HIF1a, thus increasing the expression of glycolytic enzymes LDHA and PDK1, which promotes pyruvate conversion into lactate while inhibiting pyruvate flux through the TCA cycle. As a result, B7-H3 promotes aerobic glycolysis in cancer cells and therefore tumor growth

1

BOT-1

The image appears to be a schematic representation of cellular metabolic pathways involved in cancer cell metabolism, specifically highlighting the role of B7-H3, a protein that may contribute to cancer progression.

In normal cells, glucose is processed through a series of steps to produce energy. First, it is converted into pyruvate in the cytoplasm through a process called glycolysis. Then, pyruvate is transported into the mitochondria, where it enters the tricarboxylic acid (TCA) cycle and undergoes oxidative phosphorylation (OxPhos) to generate ATP, the energy currency of the cell.

The image suggests that B7-H3 may interfere with this normal process in cancer cells. Instead of utilizing the efficient mitochondrial OxPhos, cancer cells often rely on aerobic glycolysis (also known as the Warburg effect), which is the conversion of glucose to lactate even in the presence of oxygen. This is a less efficient way to produce ATP but is thought to benefit cancer cells by redirecting carbon substrates for the synthesis of macromolecules needed for rapid cell growth and by creating a microenvironment that promotes invasion and metastasis.

Here's what the various parts of the image indicate:

1. Increased Aerobic Glycolysis: The increased conversion of glucose to lactate despite the presence of oxygen, marked by upregulation of enzymes like LDHA and PDK1.
2. Tumor Growth: This aerobic glycolysis contributes to increased tumor growth.
3. TCA Cycle and OxPhos: The diagram shows that these normal cellular processes are somehow diminished, which is typical for cancer cells.
4. HIF-1α: Hypoxia-inducible factor 1-alpha is a transcription factor that responds to low oxygen levels, often upregulated in tumors, promoting survival pathways including aerobic glycolysis.
5. NRF2: Nuclear factor erythroid 2-related factor 2 is a transcription factor that typically works to protect cells against oxidative stress. However, the image shows its activity is reduced, which might contribute to increased reactive oxygen species (ROS) in cancer cells.
6. ROS: These are damaging molecules that can contribute to cancer progression.
7. B7-H3 Receptors?: This is questioning whether there are receptors for B7-H3 on cancer cells that mediate its effects.

Overall, the image suggests a link between B7-H3 expression and metabolic changes in cancer cells that favor glycolysis over OxPhos, contributing to tumor growth and survival. The question marks next to "B7-H3 Receptors?" imply that the exact mechanism by which B7-H3 affects these pathways may still be under investigation.

BOT-2

The image you sent is a diagram of a proposed signaling pathway in cancer cells, centered on the enzyme lactate dehydrogenase A (LDHA) and the reactive oxygen species (ROS). The diagram shows how these molecules may influence tumor growth.

Here's a breakdown of the pathway illustrated in the image:

- The diagram starts with glucose entering the cancer cell. Normally, glucose would be broken down through a process called oxidative phosphorylation (OxPhos) in the mitochondria, but the diagram indicates that cancer cells tend to rely more on aerobic glycolysis, a less efficient process that produces lactate as a byproduct.
- The increase in ROS due to aerobic glycolysis is thought to activate NRF2, a transcription factor that helps the cell to defend itself against oxidative stress.
- NRF2 can also increase the levels of LDHA and PDK1, both of which can promote aerobic glycolysis.
- HIF1α is another transcription factor that can be induced by ROS. HIF1α can increase the expression of LDHA and other genes that promote tumor growth.
- B7-H3 is a receptor protein on the surface of the cancer cell. The text near the top of the image speculates that it might interact with other B7-H3 receptors
- High levels of LDHA and HIF1α can contribute to tumor growth.
- The study suggests that this pathway may be a target for cancer therapy.
- Here are some additional points to consider:
- The specific details of this pathway are still being researched.
- The Warburg effect, the tendency of cancer cells to rely on aerobic glycolysis, is not fully understood.
- B7-H3 is a protein that can inhibit the immune system's attack on cancer cells.

Overall, the image depicts a complex pathway that may be important for cancer cell growth and survival.

BOT-3

The figure you’ve shared appears to illustrate the role of **B7-H3** in regulating glucose metabolism within a cancer cell.
B7-H3 is a type of protein that has been implicated in various cellular processes, including immune responses and metabolic regulation. In the context of this figure, it seems to be involved in the regulation of glucose metabolism, a key process in both normal and cancer cells.

In normal cells, glucose is primarily metabolized through a process called oxidative phosphorylation, which is efficient and produces a lot of energy. However, cancer cells often switch to a less efficient process called aerobic glycolysis, also known as the Warburg effect. This allows them to rapidly consume glucose and produce the building blocks needed for rapid cell growth and division.

The figure suggests that B7-H3 might play a role in promoting this switch to aerobic glycolysis in cancer cells, thereby supporting tumor growth. This makes B7-H3 a potential target for therapeutic interventions aimed at disrupting cancer metabolism.

Please note that this is a general interpretation based on the description of the image. For a more accurate interpretation, it would be best to refer to the original source or consult with a specialist in the field.

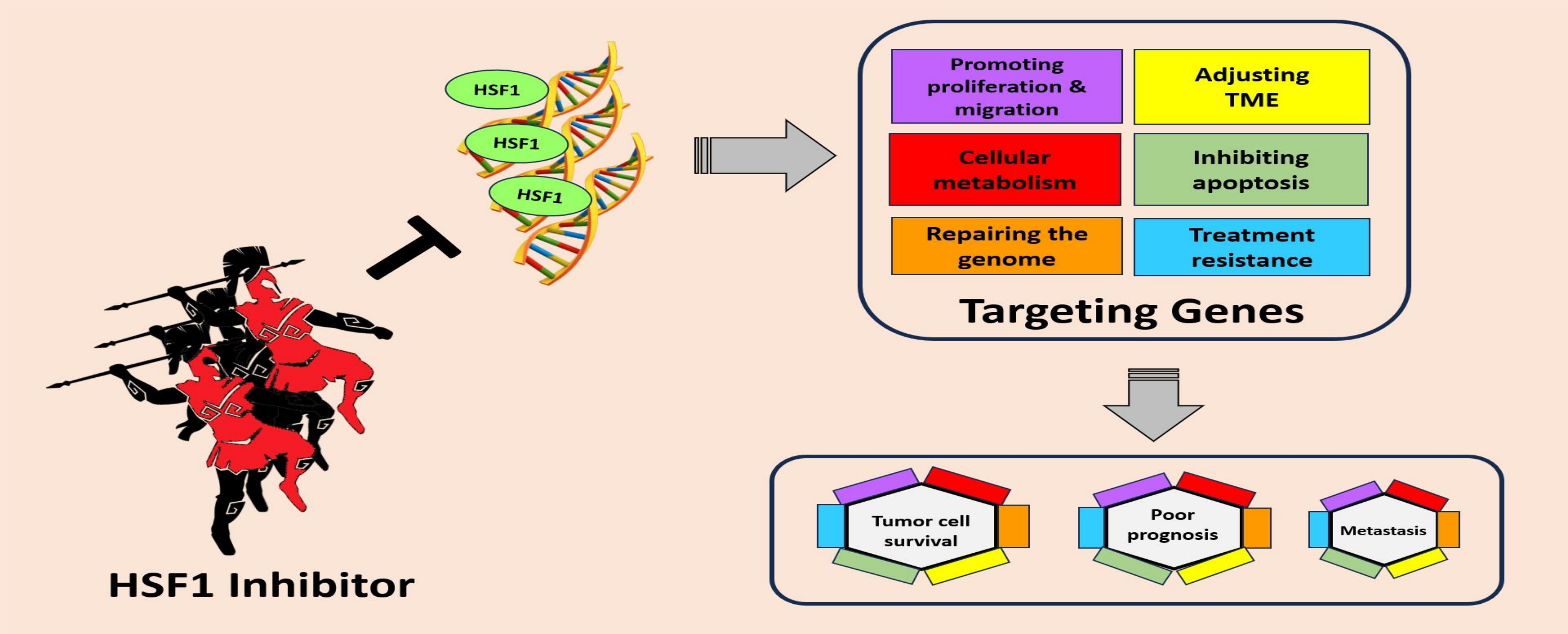

(Explanation from the original article)
HSF1 plays an important role in tumor cell survival, poor prognosis, and metastasis through several mechanisms. HSF1 can increase the expression of genes involved in anti-apoptotic mechanisms, cellular metabolism, promoting migration, and even drug resistance. By inhibiting HSF1, cancer progression can be suppressed and provide better hope to patients

BOT-1

This image appears to be an infographic or conceptual diagram related to molecular biology, specifically the role of Heat Shock Factor 1 (HSF1) in cancer and the effects of its inhibition.

Here’s a breakdown of the infographic:

- At the top left, there are multiple instances of a molecule labeled “HSF1” bound to DNA, which is represented by the iconic double helix structure. This likely represents the transcription factor HSF1 binding to DNA to regulate gene expression.
- Next to it, there’s a symbol that typically represents inhibition, leading into a box labeled “Targeting Genes.” Within this box, there are various outcomes listed that are presumably affected by HSF1 activity: promoting proliferation & migration, adjusting TME (Tumor Microenvironment), inhibiting apoptosis, cellular metabolism, repairing the genome, and treatment resistance. These are processes that HSF1 can influence, and they are all processes relevant to cancer progression and treatment response.
- Below, there is an icon of a figure riding a horse, reminiscent of a warrior or samurai, which is striking through the HSF1 with a hammer, labeled “HSF1 Inhibitor.” This metaphorically represents a treatment or substance that inhibits the action of HSF1.
- At the bottom, there are two outcomes inside hexagons, with arrows pointing down from the “Targeting Genes” box. These outcomes are “Tumor cell survival,” “Poor prognosis,” and “Metastasis.” These hexagons are likely meant to show that inhibiting HSF1 could impact these aspects of cancer, potentially leading to decreased tumor cell survival, better prognosis, and less metastasis.

The overall message seems to be that HSF1 is a factor that contributes to tumor survival and progression through various mechanisms and that inhibiting HSF1 could be a therapeutic strategy in cancer treatment, leading to better outcomes. The graphic uses symbolic and literal imagery to explain a complex biological process in a simplified manner.

BOT-2

The image you sent is a diagram depicting a novel anti-cancer strategy targeting Heat Shock Factor 1 (HSF1) for precision oncology.
Here’s a detailed interpretation:

- The left side of the image illustrates the stages of tumor progression: proliferation, migration, and metastasis.
- On the right side, it highlights the cellular effects of HSF1 inhibition, including:
  - ❑ Inhibiting proliferation and migration
  - ❑ Disrupting cellular metabolism
  - ❑ Inducing apoptosis (programmed cell death)
  - ❑ Repairing the genome
- The text “poor prognosis” is associated with tumor cell survival and metastasis, while “treatment resistance” is linked to repairing the genome.

Overall, the image suggests that HSF1 inhibition could be a promising therapeutic approach for targeting and eliminating cancer cells.
Please note that this is a complex topic and further research is recommended for a complete understanding.

BOT-3

The image you've shared appears to be a graphical representation of the effects of an HSF1 inhibitor on gene targeting related to cancer.

Here's a breakdown:

1. HSF1 Inhibition: The image shows "HSF1" being inhibited by an "HSF1 Inhibitor". HSF1 is a protein that plays a crucial role in the response to various types of stress, including heat shock and oxidative stress.
2. Targeting Genes: The inhibition of HSF1 affects several cellular activities, as represented by the box labeled "Targeting Genes". These activities include:
   - Promoting proliferation & migration
   - Adjusting the Tumor Microenvironment (TME)
   - Cellular metabolism
   - Inhibiting apoptosis
   - Repairing the genome
   - Treatment resistance
3. Outcomes: The image suggests that targeting these genes can affect:
   - Tumor cell survival
   - Prognosis
   - Metastasis

In summary, the image illustrates the potential therapeutic strategy of using an HSF1 inhibitor to target specific genes associated with tumor cell survival, prognosis, and metastasis. Please consult a healthcare professional or a researcher for more detailed information. This interpretation is based on the image and may not fully capture all the nuances of the research or therapeutic strategy depicted.

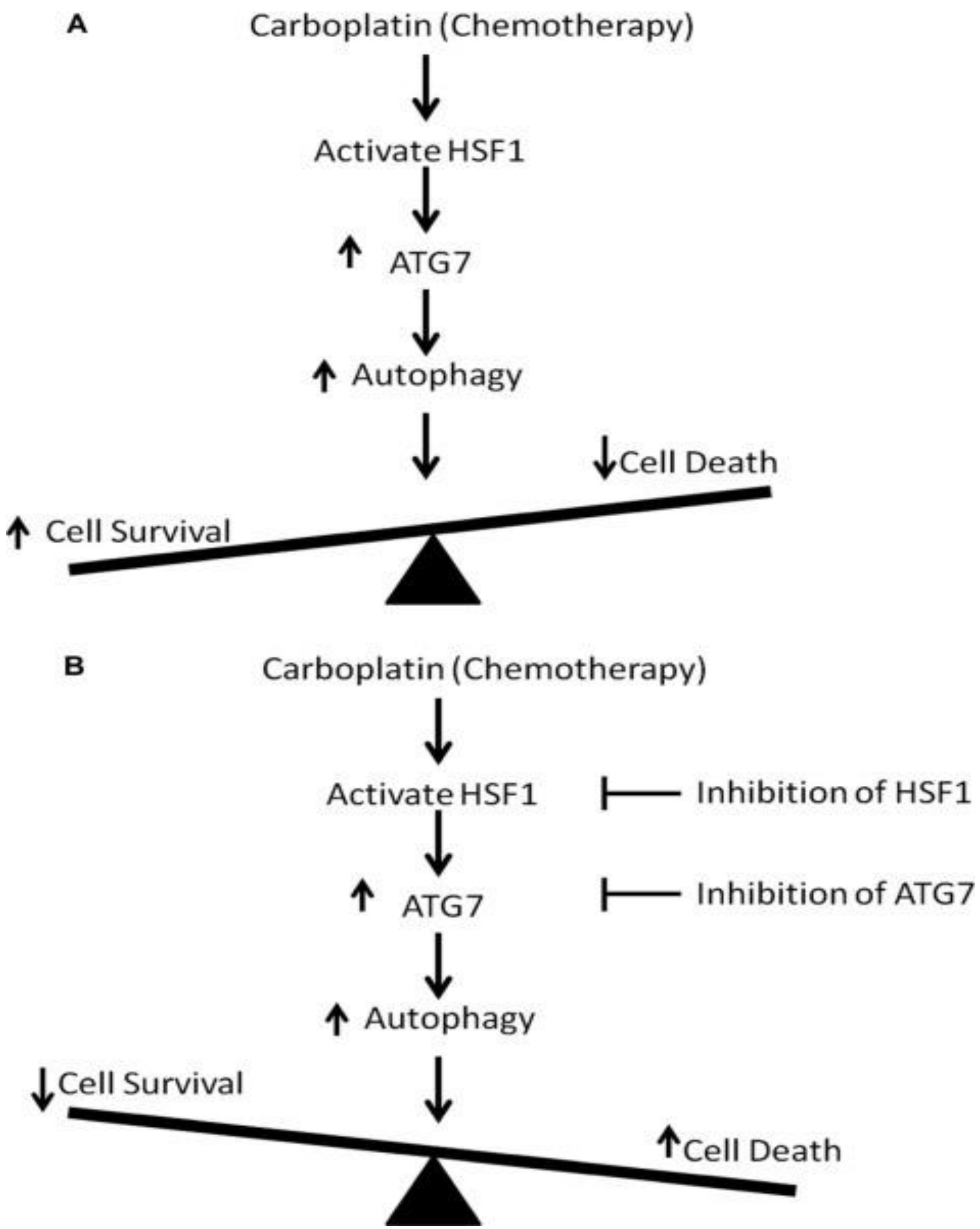

(Explanation from the original article)
A, carboplatin treatment activates HSF1 (increasing trimer formation, nuclear translocation, and phosphorylation) which allows HSF1 to bind to and up-regulate transcription of ATG7 leading to an increase in autophagy. An increase in autophagy leads to an increase in cell survival and a reduction in amount of cell death.

B, inhibition of HSF1 activity or reduction in ATG7 levels may lead to a reduction in autophagy and improve the cellular response to carboplatin.

BOT-1

The image depicts two pathways (A and B), both concerning the role of HSF1 in cancer treatment, particularly when using the chemotherapy drug carboplatin.

Pathway A:
- Carboplatin treatment leads to the activation of HSF1.
- Activated HSF1 increases the expression of ATG7, a gene that's important for the process of autophagy.
- Autophagy, a cellular degradation and recycling process, is upregulated.
- The increase in autophagy leads to decreased cell death and therefore increases cell survival, which could be undesirable in the context of cancer treatment as it might allow cancer cells to persist despite chemotherapy.

Pathway B:
- Again, carboplatin activates HSF1.
- But this time, there's an additional step where HSF1 is inhibited (not shown how).
- When HSF1 is inhibited, the activation of ATG7 is presumably decreased.
- This pathway also shows the inhibition of ATG7 independently (possibly suggesting another therapeutic target or intervention point).
- The decrease in ATG7 leads to decreased autophagy.
- Reduced autophagy results in increased cell death, which is generally the goal of chemotherapy—to kill cancer cells.

The balance in the center symbolizes the therapeutic goal of tipping the scales from cell survival towards cell death in cancer treatment. By inhibiting key players like HSF1 or ATG7, which are upregulated by chemotherapy, the treatment could be made more effective by promoting the death of cancer cells instead of their survival.

BOT-2

The correct interpretation of the image depends on whether HSF1 is activated or inhibited.

HSF1 activation

- Carboplatin, a chemotherapy drug, can activate HSF1.
- Activated HSF1 increases the expression of ATG7, a protein required for autophagy.
- Increased autophagy helps cancer cells survive by breaking down cellular components for nutrients and energy.

Overall, HSF1 activation in cancer cells can lead to chemoresistance.

HSF1 inhibition

- Carboplatin can also inhibit HSF1.
- Inhibited HSF1 reduces the expression of ATG7, thereby inhibiting autophagy.
- Decreased autophagy reduces the ability of cancer cells to survive under stress conditions.

Overall, HSF1 inhibition in cancer cells can increase their sensitivity to chemotherapy.

The image suggests that HSF1 plays a complex role in cancer cell metabolism and chemoresistance. Whether HSF1 activation or inhibition is beneficial for cancer treatment depends on the specific context.

BOT-3

The image you provided seems to illustrate the pathways of how **Carboplatin**, a chemotherapy drug, affects cancer cell metabolism through the activation and inhibition of **HSF1** and **autophagy**, leading to either cell survival or death.

Here's a brief interpretation based on the image description:

- **Carboplatin** is a chemotherapy drug used to treat various types of cancer. It works by interfering with the growth of cancer cells, which are then destroyed by the body.
- **HSF1** (Heat Shock Factor 1) is a protein that in humans is encoded by the HSF1 gene. It is a stress-responsive transcription factor that plays a central role in protecting cells from various stress conditions.
- **Autophagy** is a cellular process that breaks down and recycles cellular components when nutrients are scarce, which can promote cell survival.

In the context of cancer cell metabolism, the image suggests that Carboplatin can influence the balance between HSF1 and autophagy. This balance can determine the fate of a cancer cell, leading to either survival or death. For instance, the activation of HSF1 or inhibition of autophagy might promote cancer cell survival, while the opposite could lead to cell death.

Please note that this is a simplified interpretation and the actual biological processes are much more complex. For a more detailed understanding, it would be best to refer to scientific literature or consult with a specialist in the field.

I hope this helps! If you have any more questions or need further clarification, feel free to ask. 😊

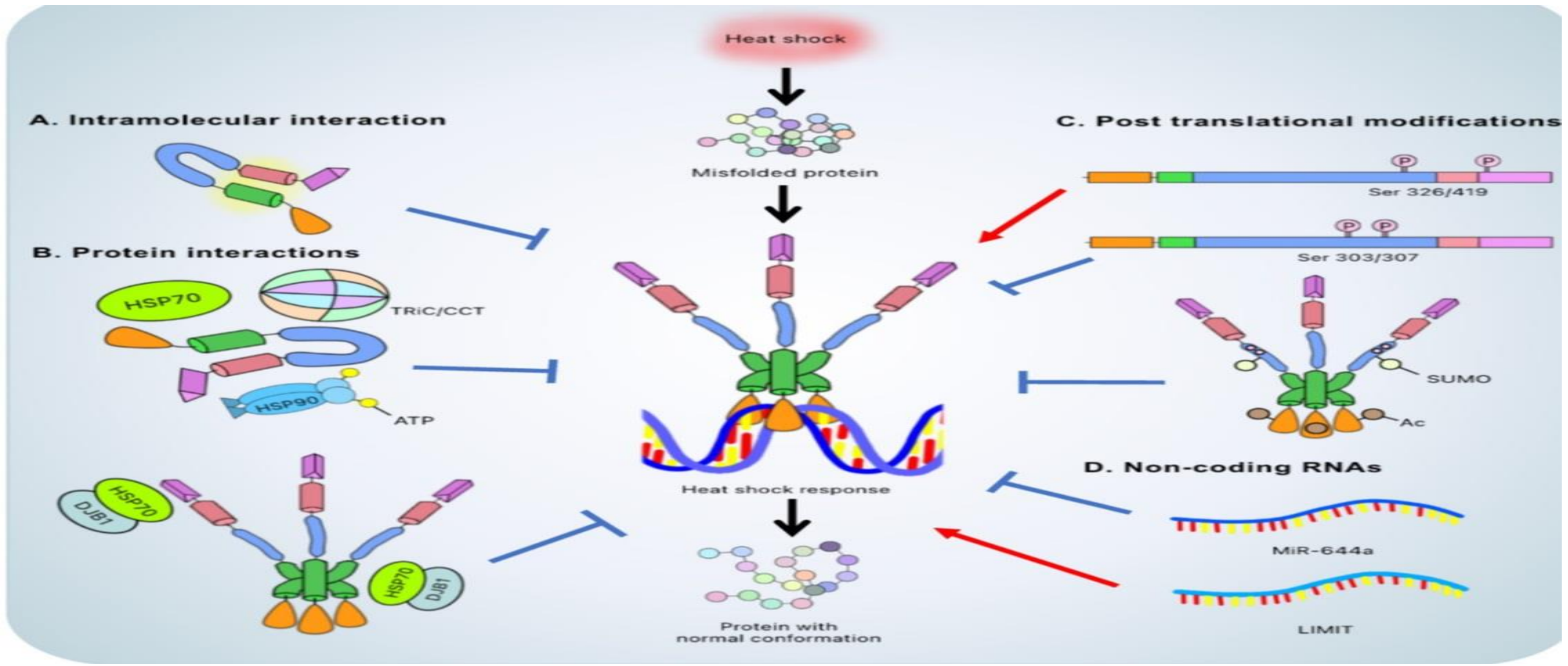

(Explanation from the original article)
The heat shock response and the regulation of HSF1. The heat shock response is induced by the HSF1 activated trimer binding to the heat shock element that leads to resolution of the heat shock induced stress and accurate protein conformation. The methods to regulate the HSF1 include:

A. intramolecular interaction between LZ1-3 and LZ4 to keep the inert monomeric state;
B. interaction with other proteins, such as ATP-binding ‘‘closed’’ HSP90, HSP70 and TRiC/CCT to keep the inert monomeric state or interaction between HSP70-DnaJB1 complex and LZ1-3 or TAD to attenuate the heat shock response;
C. post translational modification such as phosphorylation of Ser326/419 and Ser303/307 considered to be the activated and inactivated signals, respectively; acetylation of DBD and phosphorylation-dependent SUMOylation of RD to attenuate the heat shock response and **D.** non-coding RNA to inactivate and activate the heat shock response via miR-644a and LIMIT, respectively.

Color representation: DNA-binding domain (orange); leucine zipper 1-3 (green); regulatory domain (blue); leucine zipper 4 (pink); transactivation domain (purple).

BOT-1

This image appears to be a schematic representation of the heat shock response and the regulation of Heat Shock Factor 1 (HSF1), which is a transcription factor that is activated in response to stress, such as elevated temperatures. The diagram is divided into four parts, A through D, each illustrating different aspects of the process:

A. Intramolecular interaction:
This part shows a protein with a stable structure under normal conditions. Upon heat shock, the protein misfolds, which is depicted by the disorganized and tangled structure.

B. Protein interactions:
The misfolded protein then engages with molecular chaperones (HSP70 and HSP90) and co-chaperones (Hop, TRiC/CCT). The chaperones assist in the refolding of the protein, with ATP providing the energy required for this process. The end result is the restoration of the protein's normal conformation.

C. Post-translational modifications:
Here, the focus is on HSF1, which is modified after being translated. The modifications shown include phosphorylation (P) at specific serine residues (Ser 326/419 and Ser 303/307), SUMOylation (SUMO), and acetylation (Ac). These post-translational modifications can influence the activity, stability, and localization of HSF1, thus regulating the heat shock response.

D. Non-coding RNAs:
This section introduces the role of non-coding RNAs in the heat shock response. Specifically, it mentions microRNA (miR-644a) and long non-coding RNA (LIMIT). These RNAs can regulate the expression of genes involved in the heat shock response, possibly including HSF1, by interacting with their target messenger RNAs.

The red arrows typically indicate activation or an increase in activity or expression, while the blue arrows indicate a progression or flow from one state to another.
Overall, this image provides a detailed view of how cells respond to heat shock by refolding misfolded proteins with the help of chaperones and how the heat shock response is finely regulated through post-translational modifications of HSF1 and interactions with non-coding RNAs.

BOT-2

The image you sent me depicts the heat shock response and how it is regulated by HSF1 (Heat Shock Factor 1). Here's a breakdown of the cellular processes involved:

- **Heat shock:** This is the initial stressor that disrupts protein folding. When this happens, misfolded proteins accumulate in the cell.
- **Misfolded proteins:** These can cause a number of problems in the cell, including interfering with other cellular processes and protein aggregation.
- **Protein interactions:** There are several different protein interactions that can occur in response to misfolded proteins. These interactions are depicted by the letters A, B, and C in the image:
    a. Intramolecular interaction (A): This refers to the way that the misfolded protein folds in on itself.
    b. Protein interactions (B): This refers to the way that misfolded proteins interact with other proteins. In the image, this is exemplified by the TriC/CCT complex, which is a chaperone complex that helps proteins fold correctly.
    c. Post translational modifications (C): These are modifications that are made to proteins after they have been translated from RNA. In the image, this is exemplified by phosphorylation (Ser 326/419 and Ser 303/307) and SUMOylation (SUMO). Phosphorylation is the addition of a phosphate group to a protein, while SUMOylation is the addition of a small ubiquitin-like modifier (SUMO) group. Both of these modifications can affect the way that a protein folds and functions.
- **Heat shock response:** This is the cell's response to the accumulation of misfolded proteins. It is mediated by HSF1, which is a transcription factor that turns on the expression of heat shock proteins (HSPs).
- **Heat shock proteins (HSPs):** These are a family of proteins that help other proteins fold correctly and prevent protein aggregation. In the image, HSP70 and HSP90 are specifically mentioned.
- **Non-coding RNAs:** These are RNAs that do not code for proteins. Some non-coding RNAs, such as miR-644a, can regulate the heat shock response.

The takeaway from this image is that the heat shock response is a complex and tightly regulated process that helps cells to cope with the stress of misfolded proteins. HSF1 plays a central role in this process by turning on the expression of HSPs.

BOT-3

The image you’ve shared is a representation of the heat shock response and the regulation of HSF1, a crucial protein in cellular stress response. Here’s a brief interpretation based on the description:

The image illustrates the complex biological processes involved in the heat shock response, a cellular mechanism that is activated in response to increased temperatures and other stress factors. The key player in this process is Heat Shock Factor 1 (HSF1), which is regulated through various mechanisms such as intramolecular interactions, protein interactions, post-translational modifications, and non-coding RNAs.
When a cell is subjected to heat shock or other stress conditions, misfolded proteins accumulate in the cell. HSF1 plays a critical role in managing this stress by activating the transcription of heat shock proteins (HSPs). These HSPs assist in refolding the misfolded proteins, thereby restoring normal cellular function.

The image likely shows the different stages of this process, from the activation of HSF1 in response to stress, to the transcription of HSPs, and finally the refolding of misfolded proteins. It’s a visual representation of how cells manage and survive stressful conditions.

Please note that this is a high-level interpretation. The exact details might vary depending on the specific pathways and mechanisms depicted in the image. For a more detailed understanding, it would be best to refer to the source or context from which the image was taken.

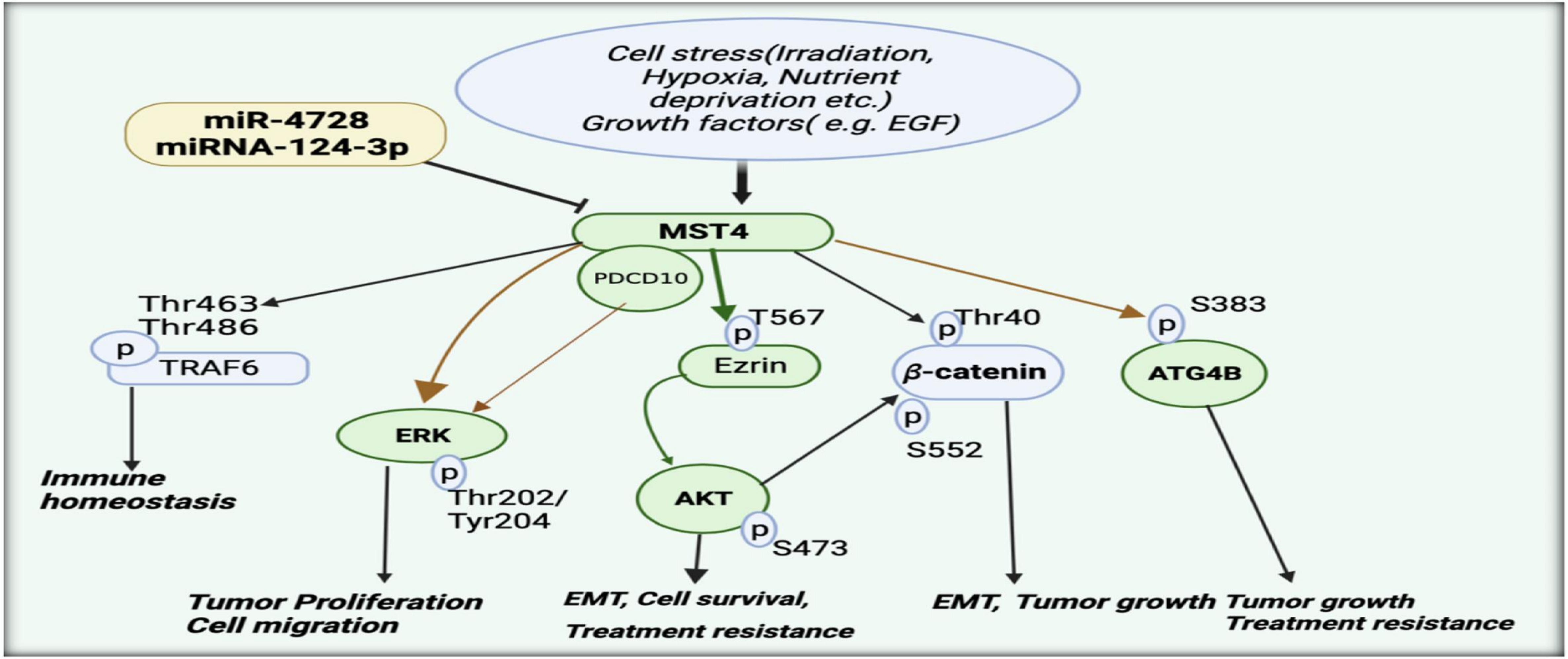

(Explanation from the original article)
Mechanisms of MST4 induced tumor progression and treatment resistance. Through the activation of several downstream signaling pathways, MST4 is contributing to tumor cell proliferation, promoting migration/invasion and metastasis of tumor cells with the activation of EMT associated molecules, and enhance tumor cell survival and resistance for therapeutics.

BOT-1

This image appears to be a schematic diagram of the molecular pathways by which the protein kinase MST4 (Mammalian Sterile20-like kinase 4) contributes to tumor progression and resistance to treatment. Here's an interpretation of the diagram:

- Cell Stress and Growth Factors: The diagram begins with external stimuli, such as cell stress (irradiation, hypoxia, nutrient deprivation, etc.) and growth factors (e.g., EGF or Epidermal Growth Factor). These stimuli can activate MST4.
- MST4 Activation: MST4, once activated, phosphorylates (as indicated by the 'P' and arrows) several downstream effector proteins.

Downstream Effectors

- PDCD10: MST4 phosphorylates PDCD10, which then appears to have a role in activating Ezrin.
- Ezrin: This protein is phosphorylated at T567 and plays a role in cell signaling pathways that promote cell survival, epithelial-mesenchymal transition (EMT), and treatment resistance.
- β-catenin: Phosphorylated at T40 and S552, β-catenin is involved in EMT and tumor growth.
- ATG4B: This is phosphorylated at S383 and is implicated in tumor growth and treatment resistance.
- ERK: Extracellular signal-regulated kinase (ERK) is phosphorylated at T202/Y204 by MST4, and this is associated with tumor proliferation, cell migration, and the EMT process.
- AKT: Also known as Protein Kinase B, phosphorylated at S473, AKT signaling is involved in EMT, cell survival, and treatment resistance.
- miR-4728 and miRNA-124-3p: These microRNAs are shown to interact with MST4, suggesting a regulatory feedback or control mechanism, though the diagram doesn't specify the nature of their effect.
- TRAF6: This is another protein involved in immune homeostasis and is phosphorylated at Thr463 and Thr486, although its direct link to MST4 in this diagram is not indicated.

The connections between proteins are shown with arrows, indicating the direction of signal transduction. The phosphorylation sites are indicated with a "P" and the specific amino acid positions (e.g., S383 means serine at position 383).

Overall, the diagram highlights a complex network of interactions and modifications that lead to the promotion of cancerous behaviors in cells, including their proliferation, their ability to invade other tissues, and their resistance to therapies aimed at killing them. Understanding these pathways can be critical for developing targeted cancer treatments.

BOT-2

The figure you sent illustrates the mechanisms by which MST4, a protein kinase, can influence tumor progression and treatment resistance. The left side of the figure depicts cellular stress factors that can activate MST4, including irradiation, hypoxia, nutrient deprivation, and growth factors. These factors can activate miR-4728 and miR-124-3p, which in turn, reduce the levels of MST4 protein.
MST4 can phosphorylate several target proteins, including PDCD10, Ezrin, β-catenin, and ATG4B. Phosphorylation of these proteins can then lead to the activation of various signaling pathways that promote tumor proliferation, EMT (epithelial-to-mesenchymal transition), cell survival, cell migration, and treatment resistance.

Here’s a more detailed breakdown of the pathways illustrated:

- **AKT pathway:** MST4 can inhibit the AKT pathway by phosphorylating AKT on threonine 308. The AKT pathway is a well-known signaling cascade that promotes cell proliferation and survival.
- **ERK pathway:** MST4 can also inhibit the ERK pathway by phosphorylating RAF on serine 259. The ERK pathway is another important signaling cascade that promotes cell proliferation and survival.
- **EMT:** MST4 can inhibit EMT by phosphorylating Ezrin. Ezrin is a protein that helps to link the cytoskeleton to the plasma membrane. When Ezrin is phosphorylated by MST4, it can no longer interact with β-catenin, which is a key EMT transcription factor.
- **Autophagy:** MST4 can inhibit autophagy by phosphorylating ATG4B. ATG4B is a protein that is required for autophagosome formation. Autophagy is a cellular process that can help cancer cells to survive during periods of stress.

Overall, the figure suggests that MST4 can function as a tumor suppressor by inhibiting several signaling pathways that promote tumor progression and treatment resistance. However, MST4 protein levels can be reduced by certain cellular stresses and growth factors, which may allow tumors to escape MST4-mediated suppression.

BOT-3

The image you've shared appears to illustrate the complex pathways and interactions involved in MST4 (Mammalian Sterile 20-like kinase 4) induced tumor progression and treatment resistance.

Here's a brief interpretation based on the image description:

- The image highlights the roles of various molecules and proteins such as **miR-4728**, **miRNA-124-3p**, **ERK**, **AKT**, and others in this process. These molecules and proteins are key players in the cellular mechanisms of MST4-induced tumor progression and treatment resistance.
- The diagram likely represents the intricate molecular interactions and pathways that contribute to the progression of a tumor and its resistance to treatment when MST4 is induced.
- The pathways shown might be signaling routes that these molecules take within a cell, influencing various cellular processes that could lead to tumor growth and resistance to treatment.

Please note that this is a high-level interpretation. For a detailed understanding, it would be best to refer to the source or consult with a specialist in this field. The exact mechanisms can be quite complex and are often the subject of ongoing research.

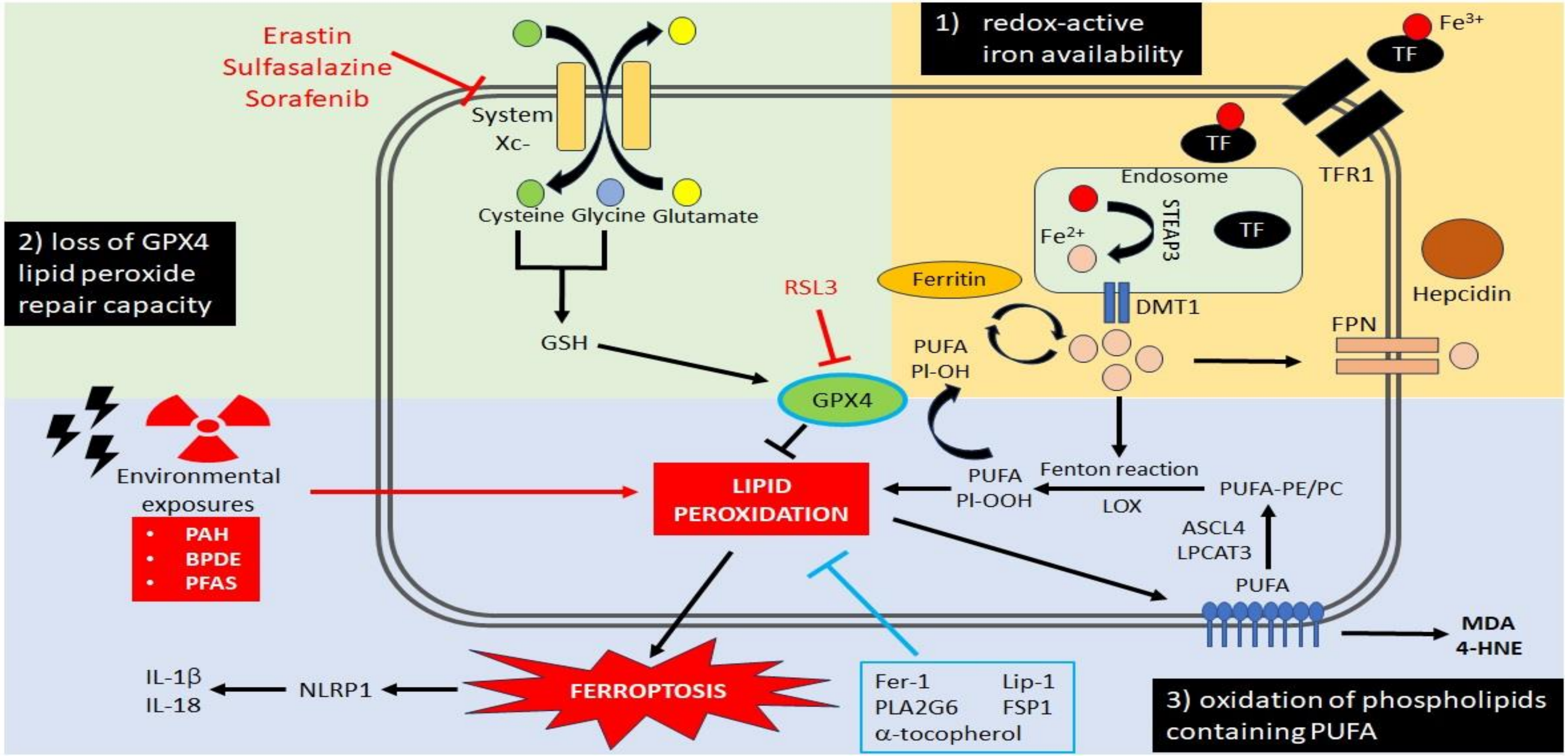

(Explanation from the original article)

Three hallmarks of ferroptosis. Ferroptosis occurs via three separate routes that can be identified. The increase of intracellular Fe2+ indicates redox-active iron availability. This occurrence initiates the Fenton reaction, which results in lipid peroxidation. The second mechanism involves the loss of GPX4 capacity, a powerful antioxidant capable of inhibiting lipid peroxidation and protecting cells from harmful PUFA PI-OOH. GPX4 is the primary regulator of ferroptosis and is essential in this situation. The oxidation of PUFA, which makes up the cell membrane, is the final feature of ferroptosis. Excessive lipid peroxidation converts PUFA into PUFA PI-OOH, and disintegrates the cell membrane, allowing the cells to undergo ferroptosis

BOT-1

This image is a diagram illustrating the biochemical process of ferroptosis, which is a form of programmed cell death distinguished by the accumulation of lipid peroxides. It's divided into three main pathways or steps:

1. Redox-Active Iron Availability:
    - Iron is transported into cells via transferrin (TF) and transferrin receptor 1 (TFR1). The iron is then released into the cytosol through endocytosis and the action of divalent metal transporter 1 (DMT1).
    - Ferritin stores iron, and ferroportin (FPN) exports it from the cell.
    - The diagram shows that redox-active iron contributes to the production of reactive oxygen species (ROS), which can lead to lipid peroxidation.

2. Loss of GPX4 Lipid Peroxide Repair Capacity:
    - Glutathione peroxidase 4 (GPX4) is an enzyme that repairs lipid peroxides, preventing them from causing cell damage.
    - The System Xc- imports cysteine into the cell in exchange for glutamate. Cysteine is necessary for the synthesis of glutathione (GSH), which is crucial for the function of GPX4.
    - Certain drugs like Erastin, Sulfasalazine, and Sorafenib, and environmental exposures such as to polycyclic aromatic hydrocarbons (PAH), benzo[a]pyrene diol epoxide (BPDE), and perfluoroalkyl substances (PFAS) can inhibit System Xc-, leading to a decrease in GPX4 activity and thus, lipid peroxide accumulation.

3. Oxidation of Phospholipids Containing Polyunsaturated Fatty Acids (PUFA) :
    - Phospholipids containing PUFA can be oxidized through non-enzymatic reactions (like the Fenton reaction) or enzymatic pathways involving lipoxygenases (LOX).
    - Products of lipid peroxidation such as malondialdehyde (MDA) and 4-hydroxynonenal (4-HNE) are toxic to cells and are markers of oxidative stress.

Finally, the image shows the end result of these pathways: ferroptosis, which is a type of cell death characterized by the overwhelming accumulation of lipid peroxides. It also points out several inhibitors of ferroptosis such as Fer-1, Lip-1, PLA2G6, FSP1, and alpha-tocopherol, which are molecules that can inhibit different points in the pathway to prevent cell death. Conversely, the activation of NLRP1 and subsequent release of inflammatory cytokines like IL-1β and IL-18 are associated with the ferroptosis process.

This type of diagram is often used in biochemical research and medical education to explain complex cellular processes.

BOT-2

The image you sent is a diagram of the ferroptosis pathway, a form of programmed cell death. Ferroptosis is triggered by the iron-dependent accumulation of lipid peroxides.

Here's a breakdown of the image with some additional information:

- Environmental Exposures: These can include heavy metals, ultraviolet radiation, and certain medications. They can disrupt the redox balance in cells and promote ferroptosis.
- Erastin, Sulfasalazine, Sorafenib: These are all chemical compounds that can induce ferroptosis.
- System Xc- and SLC7A11: System Xc- is an antiporter protein complex that exchanges glutamate for cystine. SLC7A11 is the gene that codes for the light chain of System Xc-. Erastin is shown to inhibit System Xc-.
- GPX4: Glutathione peroxidase 4 is an enzyme that helps protect cells from ferroptosis by reducing lipid hydroperoxides.
- Lipid Peroxidation: This is a process where polyunsaturated fatty acids (PUFAs) in cell membranes are damaged by free radicals. In the context of ferroptosis, iron promotes lipid peroxidation.
- Ferroptosis executioner proteins: These include lipoxygenases (LOXs), acyl-CoA synthetase 4 (ACSL4), lysophosphatidylcholine acyltransferase 3 (LPCAT3) and monoamine oxidase A (MAOA). They are all enzymes that are involved in pathways that lead to ferroptosis.
- Ferroptosis markers: These include malondialdehyde (MDA), 4-hydroxynonenal (4-HNE), and stearoyl-CoA desaturase (SCD). Their levels increase in cells undergoing ferroptosis.

The overall process depicted in the image is that environmental exposures or certain medications can disrupt the redox balance in cells and lead to iron-dependent lipid peroxidation. This, in turn, can trigger ferroptosis through the action of ferroptosis executioner proteins. Ferroptosis markers are produced as byproducts of the pathway.

BOT-3

The image you've uploaded is a detailed diagram explaining the process of ferroptosis, a regulated form of cell death driven by iron-dependent lipid peroxidation.

It outlines the biochemical reactions and pathways involved, including:

1. Redox-active iron availability: This pathway involves the availability of iron in a form that can participate in redox reactions.
2. Loss of GPX4 lipid peroxide repair capacity: GPX4 is an enzyme that repairs lipid peroxides, preventing them from causing damage to the cell. Its loss can lead to an increase in lipid peroxidation.
3. Oxidation of phospholipids containing PUFA: Phospholipids containing polyunsaturated fatty acids (PUFA) are susceptible to oxidation, which can lead to cell damage and eventually ferroptosis.

The diagram also shows how environmental exposures like PAH, PBDE, PFAS can affect the loss of GPX4 lipid peroxide repair capacity. Specific chemicals like Erastin, Sulfasalazine, and Sorafenib are indicated as inhibitors in the system Xc-, affecting cysteine uptake.

The arrows in the diagram indicate the flow and interaction between different elements leading to lipid peroxidation, which then leads to ferroptosis. Various molecules and compounds are represented with different shapes and colors.
Please note that this is a high-level interpretation of the diagram. For a detailed understanding, it would be best to refer to a textbook or scientific literature on cell biology or consult with a subject matter expert.

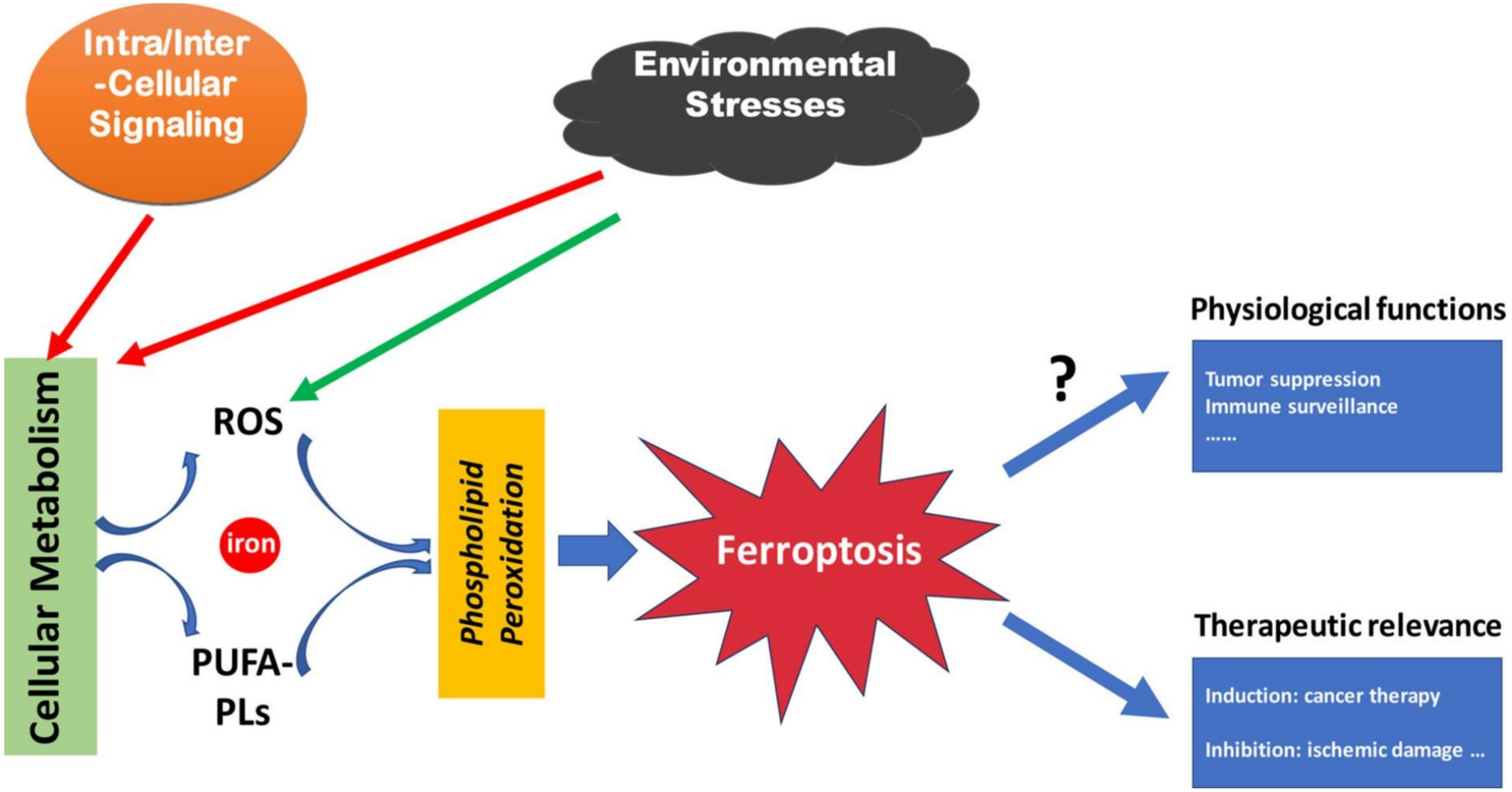

This is a picture of "An Overview of ferroptosis" Please give a suitable interpretation of this picture

(Explanation from the original article)
ferroptosis is executed by phospholipid peroxidation, a process relying on metabolic products reactive oxygen species (ROS), phospholipid containing polyunsaturated fatty acid chain(s) (PUFA-PL), and transition metal iron, and that intra- and intercellular signaling events and environmental stresses can impact ferroptosis by regulating cellular metabolism and ROS level. The figure also shows the role of ferroptosis in disease and its potential physiological functions.

BOT-1

This image provides an overview of ferroptosis, a form of regulated cell death driven by iron-dependent lipid peroxidation. Here's an interpretation of its key elements:

1. **Triggers of Ferroptosis**:
    - **Intra/Inter-Cellular Signaling**: Signaling pathways influence cellular metabolism, impacting ferroptosis.
    - **Environmental Stresses**: External factors such as oxidative stress, nutrient depletion, or toxins contribute to initiating ferroptosis.
2. **Central Role of Cellular Metabolism**:
    - Cellular metabolism generates reactive oxygen species (ROS), which, in conjunction with polyunsaturated fatty acid phospholipids (PUFA-PLs) and iron, promote phospholipid peroxidation.
    - Phospholipid peroxidation is a crucial step leading to ferroptosis.
3. **Mediators of Ferroptosis**:
    - **ROS** (Reactive Oxygen Species): Produced during cellular metabolism, they initiate oxidative damage.
    - **Iron**: Catalyzes ROS production and lipid peroxidation.
    - **PUFA-PLs**: Target lipids for peroxidation.
4. **Outcomes of Ferroptosis**:
    - **Physiological Functions**: It plays roles in tumor suppression, immune surveillance, and potentially other processes (indicated by the question mark).
    - **Therapeutic Relevance**:
        - **Induction**: Ferroptosis induction is explored in cancer therapy to eliminate cancer cells.
        - **Inhibition**: Blocking ferroptosis can protect against ischemic damage or neurodegenerative diseases.

This figure highlights ferroptosis as a process interlinked with metabolism, oxidative stress, and lipid peroxidation, with significant implications for physiological functions and therapeutic strategies.

BOT-2

This image provides a good overview of ferroptosis, a type of regulated cell death, by highlighting its key features:

1. Central Mechanism:

- **Phospholipid peroxidation:** This is the core process driving ferroptosis. It involves the oxidation of specific phospholipids (PLs) in the cell membrane that contain polyunsaturated fatty acids (PUFAs). This peroxidation is depicted as the yellow box leading to the red "Ferroptosis" burst.

2. Key Players:

- **ROS (Reactive Oxygen Species):** These highly reactive molecules play a crucial role in initiating and propagating lipid peroxidation. The red arrow from "Environmental Stresses" emphasizes that external factors can increase ROS.
- **Iron:** Essential for ferroptosis, likely by catalyzing the production of ROS through the Fenton reaction.

3. Triggers:

- **Intra/Inter-Cellular Signaling:** Signals from within the cell or from neighboring cells can initiate ferroptosis. The red arrow suggests this can increase ROS.
- **Environmental Stresses:** External factors like radiation, toxins, and nutrient deprivation can trigger ferroptosis, often by increasing ROS (green arrow).

4. Physiological Functions:

•While not fully understood, ferroptosis is believed to play a role in:

- •**Tumor suppression:** By eliminating damaged or cancerous cells.
- •**Immune surveillance:** Aiding the immune system in recognizing and destroying abnormal cells.

5. Therapeutic Relevance:

•**Induction:** Promoting ferroptosis could be a strategy for cancer therapy by selectively killing tumor cells.

•**Inhibition:** Blocking ferroptosis might protect against damage caused by conditions like ischemia (reduced blood flow), where excessive cell death occurs.

In Summary: This image effectively illustrates the key components and processes involved in ferroptosis, emphasizing its causes, consequences, and potential therapeutic applications.

BOT-3

The image titled "An Overview of Ferroptosis" illustrates the process and implications of ferroptosis, a type of regulated cell death. The diagram highlights several key components and interactions:

1. **Cellular Metabolism**: This is shown as a green box and is influenced by intra/inter-cellular signaling (orange oval) and environmental stresses (black cloud). Both signaling and stresses have arrows pointing towards cellular metabolism, indicating their influence.

2. **Reactive Oxygen Species (ROS)**: Cellular metabolism leads to the production of ROS, depicted by a green arrow. ROS is central to the process and is influenced by iron (red circle) and polyunsaturated fatty acid-phospholipids (PUFA-PLs), shown with blue arrows.

3. **Phospholipid Peroxidation**: This is shown as a yellow box and is a process influenced by ROS, leading to ferroptosis (red starburst).

4. **Ferroptosis**: The central event in the diagram, shown as a red starburst, is the result of phospholipid peroxidation.

5. **Physiological Functions**: Ferroptosis has potential physiological functions such as tumor suppression and immune surveillance, indicated by a blue arrow pointing to a blue box with a question mark.

6. **Therapeutic Relevance**: Ferroptosis has therapeutic relevance, with potential for induction in cancer therapy and inhibition in ischemic damage, shown by another blue arrow pointing to a blue box.

This image provides a visual summary of the complex interactions leading to ferroptosis and its potential implications in physiology and therapy. It's interesting and relevant for understanding the cellular mechanisms and the potential clinical applications of this type of cell death.

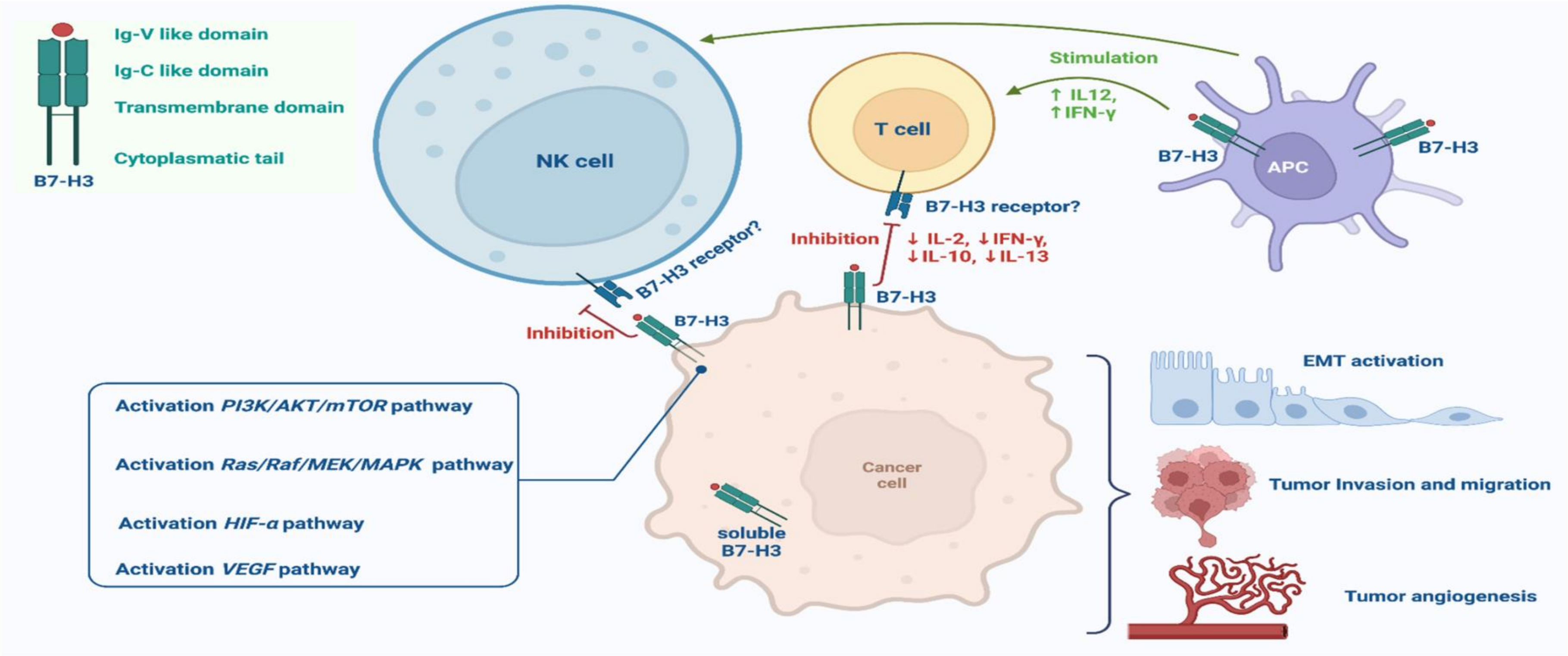

This is a picture of "B7-H3 non-immune-mediated and immune-mediated signaling pathways" Please give a suitable interpretation of this picture

(Explanation from the original article)
B7-H3 non-immune-mediated (box in the lower left corner) and immune-mediated (cartoon) signaling pathways. *PI3K/AKT/mTOR* and *Ras/Raf/MEK/MAPK* signaling pathways are involved in promoting the migration, invasion, and epithelial–mesenchymal transition (ETM) of cancer cells. *HIF-α* pathway is involved in glucose metabolic reprogramming and enhancing neoplastic tumor growth. *VEGF* signaling pathway is involved in promoting neo-angiogenesis and metastasis. B7-H3 acts as immune co-stimulatory molecule, increasing IFN-γ and IL-12 levels and promoting CD4+ and CD8+ T cells' proliferation and enhancing cytotoxic T cell activity. Moreover, B7-H3 plays an immune co-inhibitory role, reducing cytokines (IL-2, IL-10, IL-13, and IFN-γ) and inhibiting T cell proliferation and NK cell activity.

This figure illustrates the dual roles of B7-H3 in immune-mediated and non-immune-mediated signaling pathways, particularly in cancer biology. Here's an interpretation of the key elements:

Structure of B7-H3The top left shows the structure of B7-H3 with Ig-V like domains, Ig-C like domains, transmembrane domain, and cytoplasmic tail.

Immune-Mediated Effects of B7-H3

1. Interaction with T Cells:
   - Inhibition: B7-H3 downregulates key cytokines such as IL-2, IFN-γ, IL-10, and IL-13, leading to reduced T cell activity.
   - The specific receptor for B7-H3 on T cells remains unidentified (indicated by a question mark).

2. Interaction with NK Cells:

NK cells may also be inhibited by B7-H3, though the specific receptor is unknown.

3. Interaction with Antigen-Presenting Cells (APCs):

B7-H3 expressed on APCs enhances the production of pro-inflammatory cytokines such as IL-12 and IFN-γ, which stimulate immune responses.

Non-Immune-Mediated Effects of B7-H3 in Cancer Cells

1. Signaling Pathways Activated by B7-H3:
   - PI3K/AKT/mTOR Pathway: Promotes cell growth and survival.
   - Ras/Raf/MEK/MAPK Pathway: Drives cell proliferation and differentiation.
   - HIF-α Pathway: Supports hypoxia tolerance in tumors.
   - VEGF Pathway: Facilitates angiogenesis.
2. Effects on Tumor Progression:
   - EMT Activation: B7-H3 triggers epithelial-mesenchymal transition, increasing cancer cell motility.
   - Tumor Invasion and Migration: B7-H3 facilitates the spread of cancer cells to surrounding tissues.
   - Tumor Angiogenesis: B7-H3 promotes the formation of blood vessels to supply nutrients to tumors.
3. Soluble B7-H3:
   - Released by cancer cells and may enhance the tumor microenvironment by activating these pathways.

Overall Significance

- Immune Suppression: B7-H3 inhibits T cell and NK cell functions, helping cancer cells evade immune detection.
- Tumor Promotion: Through non-immune signaling pathways, B7-H3 supports tumor growth, invasion, and angiogenesis.
- Therapeutic Implications: Targeting B7-H3 could simultaneously restore immune responses and block tumor-promoting pathways, making it a promising therapeutic target in cancer treatment.

BOT-2

This image depicts the complex roles of B7-H3, an immune checkpoint molecule, in both immune and non-immune-related pathways, particularly in the context of cancer. Let's break down the key components:

1. B7-H3 Structure:

- The image shows B7-H3 as a transmembrane protein with:
  - **Ig-V like domain and Ig-C like domain:** These are extracellular regions that likely interact with other molecules.
  - **Transmembrane domain:** Anchors the protein in the cell membrane.
  - **Cytoplasmic tail:** Extends into the cell's interior and may participate in intracellular signaling.

2. Immune-Mediated Signaling:

- **Stimulation:** B7-H3 on antigen-presenting cells (APCs) can stimulate T cells and NK cells, leading to increased production of cytokines like IL-12 and IFN-γ, which promote an anti-tumor immune response.
- **Inhibition:** However, B7-H3 can also inhibit T cell activity, reducing the production of IL-2 and IFN-γ (which help fight tumors) and increasing IL-10 and IL-13 (which can suppress the immune response). This suggests a complex and context-dependent role in immune regulation.

3. Non-Immune-Mediated Signaling:

- **Cancer cell:** B7-H3 is often expressed on cancer cells, where it can activate several signaling pathways involved in tumor growth and progression:
  - **PI3K/AKT/mTOR pathway:** Promotes cell survival and growth.
  - **Ras/Raf/MEK/MAPK pathway:** Regulates cell proliferation and differentiation.
  - **HIF-α pathway:** Involved in angiogenesis (formation of new blood vessels) and adaptation to low oxygen conditions.
  - **VEGF pathway:** Stimulates angiogenesis, providing tumors with nutrients and oxygen.

4. Tumor-Promoting Effects:

- **EMT activation:** B7-H3 can promote epithelial-mesenchymal transition (EMT), a process where cells lose their adhesion and gain migratory properties, leading to:
  - **Tumor invasion and migration:** Cancer cells spread to surrounding tissues.
  - **Tumor angiogenesis:** Formation of new blood vessels that support tumor growth.

5. Soluble B7-H3:

- The presence of soluble B7-H3 suggests that it can be shed from the cell surface and potentially act as a signaling molecule in the tumor microenvironment.

In Summary: This image highlights the multifaceted nature of B7-H3, illustrating its involvement in both immune regulation and cancer progression. It emphasizes the potential of B7-H3 as a therapeutic target, where blocking its inhibitory effects or enhancing its stimulatory functions could be beneficial in cancer treatment.

BOT-3

The image illustrates the B7-H3 non-immune-mediated and immune-mediated signaling pathways, showcasing how B7-H3, a protein expressed on various cells, interacts and influences these cells' behaviors.

Key Elements:

1. B7-H3 Structure:
- Ig-V-like domain and Ig-C-like domain: These domains are part of the structure of B7-H3.
- Transmembrane domain: Anchors B7-H3 to the cell membrane.
- Cytoplasmic tail: Extends into the cell interior.

2. NK Cell Interaction:
- B7-H3 interacts with Natural Killer (NK) cells. The receptor for B7-H3 is uncertain, marked with a question mark.
- This interaction potentially inhibits NK cell function.

3. T Cell Interaction:
- B7-H3 binds to T cells, leading to inhibition and decreased production of cytokines such as IL-2, IFN-γ, IL-10, and IL-13.
- Similar to NK cells, the exact receptor on T cells is not definitively identified (question mark).

4. APC Interaction:
- On Antigen-Presenting Cells (APCs), B7-H3 stimulates the increase of cytokines like IL-12 and IFN-γ.

5. Cancer Cell Interaction:

B7-H3 on cancer cells inhibits immune responses and activates several key pathways:
- PI3K/AKT/mTOR pathway
- Ras/Raf/MEK/MAPK pathway
- HIF-1α pathway
- VEGF pathway

These pathways lead to:
- Epithelial-Mesenchymal Transition (EMT): Contributes to cancer cell invasion and migration.
- Tumor angiogenesis: The formation of new blood vessels to supply the tumor.

Overall Significance:

The image highlights the dual role of B7-H3 in both immune regulation and cancer progression. By affecting NK cells, T cells, and APCs, B7-H3 can modulate immune responses. Its role in cancer cells, particularly in activating pathways that promote tumor growth and spread, makes it a potential target for therapeutic interventions. This dual function underscores the complexity and significance of B7-H3 in cancer biology and immunology.

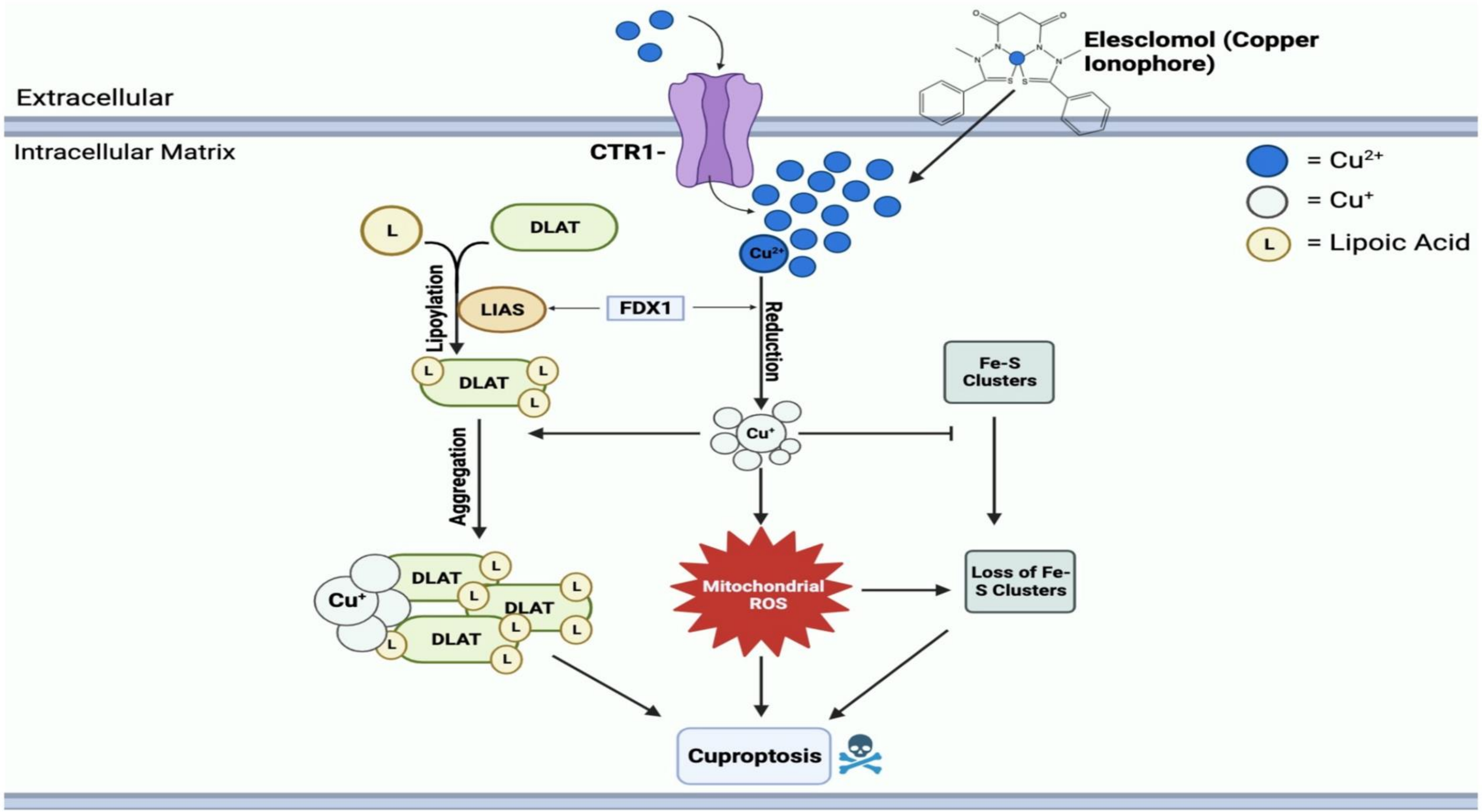

This is a picture of "Mechanisms of copper-induced cell death"

Please give a suitable interpretation of this picture

(Explanation from the original article)
Elesclomol and CTR1 shuttle Cu^{2+} into the mitochondrial matrix, creating an accumulation of copper. FDX1 reduces Cu^{2+} to Cu^{+}, resulting in ROS formation. Cu^{+} and ROS block the synthesis of Fe-S clusters. FDX1 also binds to LIAS, promoting the lipoylation of DLAT. Cu^{+} further binds to lipoylated DLAT and induces aggregation. Mitochondrial ROS, loss of Fe-S clusters, and DLAT aggregation all contribute to cuproptosis cell death.

BOT-1

This image illustrates the mechanisms of copper-induced cell death, also known as **cuproptosis**, highlighting the role of copper (Cu) accumulation and its impact on mitochondrial processes. Here's an interpretation of the key elements:

Copper Transport and Accumulation

1. **CTR1 (Copper Transporter 1)**:
   - CTR1 facilitates the uptake of extracellular **Cu^{2+}** ions into the intracellular matrix.
   - Once inside the cell, Cu^{2+} is reduced to **Cu^{+}**.
2. **Elesclomol (Copper Ionophore)**:
   - Elesclomol enhances the intracellular transport of Cu^{2+}, increasing its availability within the cell.

Copper Interaction with Cellular Components

1. **Lipoic Acid and Lipoylation**:
   - **Lipoic Acid (L)** is a key cofactor for mitochondrial enzymes, particularly **DLAT** (Dihydrolipoamide S-acetyltransferase).
   - **LIAS (Lipoic Acid Synthetase)**, regulated by **FDX1 (Ferredoxin 1)**, catalyzes lipoylation of DLAT.
   - Copper ions interact with lipoylated DLAT, leading to protein aggregation.
2. **Iron-Sulfur (Fe-S) Clusters**:
   - Copper disrupts Fe-S cluster assembly, causing their loss and impairing mitochondrial function.

Mitochondrial Dysfunction and ROS Production

1. **Mitochondrial Reactive Oxygen Species (ROS)**:
   - Copper-induced aggregation and Fe-S cluster loss lead to excessive mitochondrial ROS production.
   - ROS contribute to oxidative stress and cellular damage.

Cuproptosis Pathway

1. **Protein Aggregation**:
   - Copper interacts with lipoylated DLAT, promoting its aggregation and further mitochondrial dysfunction.
2. **Mitochondrial Damage**:
   - Loss of Fe-S clusters and elevated ROS levels collectively impair mitochondrial metabolism.
3. **Cell Death**:
   - These disruptions result in **cuproptosis**, a form of programmed cell death dependent on mitochondrial copper overload.

Therapeutic Implications

- **Induction of Cuproptosis**:
  - In cancer therapy, enhancing copper accumulation or using copper ionophores like Elesclomol can selectively target cancer cells with high mitochondrial activity.
- **Copper Homeostasis**:
  - Understanding cuproptosis mechanisms may aid in designing treatments for diseases associated with copper dysregulation.

Conclusion

This figure highlights the unique dependence of cuproptosis on mitochondrial processes, particularly the interplay between copper, lipoylated proteins, and Fe-S clusters. It underscores the therapeutic potential of manipulating copper metabolism to induce cell death in specific pathological contexts, such as cancer.

BOT-2

This image illustrates the mechanism of cuproptosis, a recently discovered form of regulated cell death triggered by excess copper. Here's a breakdown:

1. Copper Entry:
- **CTR1:** Copper enters the cell primarily through the CTR1 transporter, depicted as the purple channel on the cell membrane.
- **Elesclomol:** This copper ionophore (a molecule that helps transport ions across cell membranes) is shown facilitating copper influx, likely increasing intracellular copper levels.

2. Key Players:
- **DLAT:** Dihydrolipoamide S-acetyltransferase, a key enzyme in the tricarboxylic acid (TCA) cycle, plays a central role in cuproptosis.
- **Lipoic Acid (L):** An essential cofactor attached to DLAT, crucial for its function.
- **FDX1:** Ferredoxin 1, an iron-sulfur protein involved in cellular redox reactions.
- **LIAS:** Lipoyl synthase, the enzyme responsible for attaching lipoic acid to DLAT.

3. The Cuproptosis Pathway:
- **Copper binding:** Copper ions (Cu) bind directly to lipoylated DLAT.
- **Protein aggregation:** This copper binding leads to DLAT aggregation, forming clumps of dysfunctional proteins.
- **Loss of Fe-S clusters:** Copper also disrupts iron-sulfur clusters in proteins like FDX1, impairing their function.
- **Mitochondrial ROS:** These events ultimately lead to increased production of reactive oxygen species (ROS) in the mitochondria, the cell's powerhouses.
- **Cuproptosis:** The accumulation of ROS and dysfunctional proteins triggers cuproptosis, leading to cell death (represented by the skull and crossbones).

In Summary: This image effectively outlines the key steps involved in cuproptosis, highlighting the roles of copper, DLAT, lipoic acid, and iron-sulfur clusters. It emphasizes the importance of mitochondrial dysfunction and ROS production in this process. Understanding cuproptosis may offer new therapeutic strategies for diseases like cancer by manipulating copper levels to selectively induce tumor cell death.

BOT-3

The image titled "Mechanisms of Copper-Induced Cell Death" illustrates how copper ions (Cu^{2+}) trigger cell death, focusing on the process known as cuproptosis. Here's a step-by-step breakdown:

1. Copper Ion Transport:
   - CTR1 Transporter: Copper ions (Cu^{2+}) enter the cell through the CTR1 transporter.
   - Elesclomol: Acts as a copper ionophore, facilitating the transport of Cu^{2+} into the cell.

2. Reduction of Copper Ions:
   - Inside the cell, Cu^{2+} is reduced to Cu^{+} (copper in a lower oxidation state).

3. Lipoylation and Aggregation:
   - The enzyme LIAS (Lipoic Acid Synthetase), assisted by FDX1 (Ferredoxin 1), facilitates the lipoylation of DLAT (Dihydrolipoamide S-Acetyltransferase).
   - Lipoylated DLAT then binds with Cu^{+}, causing aggregation.

4. Mitochondrial Reactive Oxygen Species (ROS):
   - The aggregation of Cu^{+} with lipoylated DLAT leads to the production of mitochondrial ROS.

5. Fe-S Clusters:
   - Copper ions cause the disruption and loss of iron-sulfur (Fe-S) clusters within the cell.

6. Cuproptosis:
   - The combined effects of mitochondrial ROS and the loss of Fe-S clusters result in cell death via cuproptosis, characterized by mitochondrial dysfunction and metabolic chaos.

Key Components:

- Cu^{2+} (Blue Circles)
- Cu^{+} (White Circles)
- Lipoic Acid (L, Yellow Circles)
- CTR1 Transporter
- Elesclomol (Copper Ionophore)
- DLAT (Green)
- LIAS (Orange)
- FDX1 (Blue Box)
- Fe-S Clusters (Gray Box)
- Mitochondrial ROS (Red Burst)
- Cuproptosis (Skull and Crossbones)

This detailed pathway highlights how copper toxicity leads to cell death, an essential concept for understanding its potential therapeutic implications and toxicological risks.

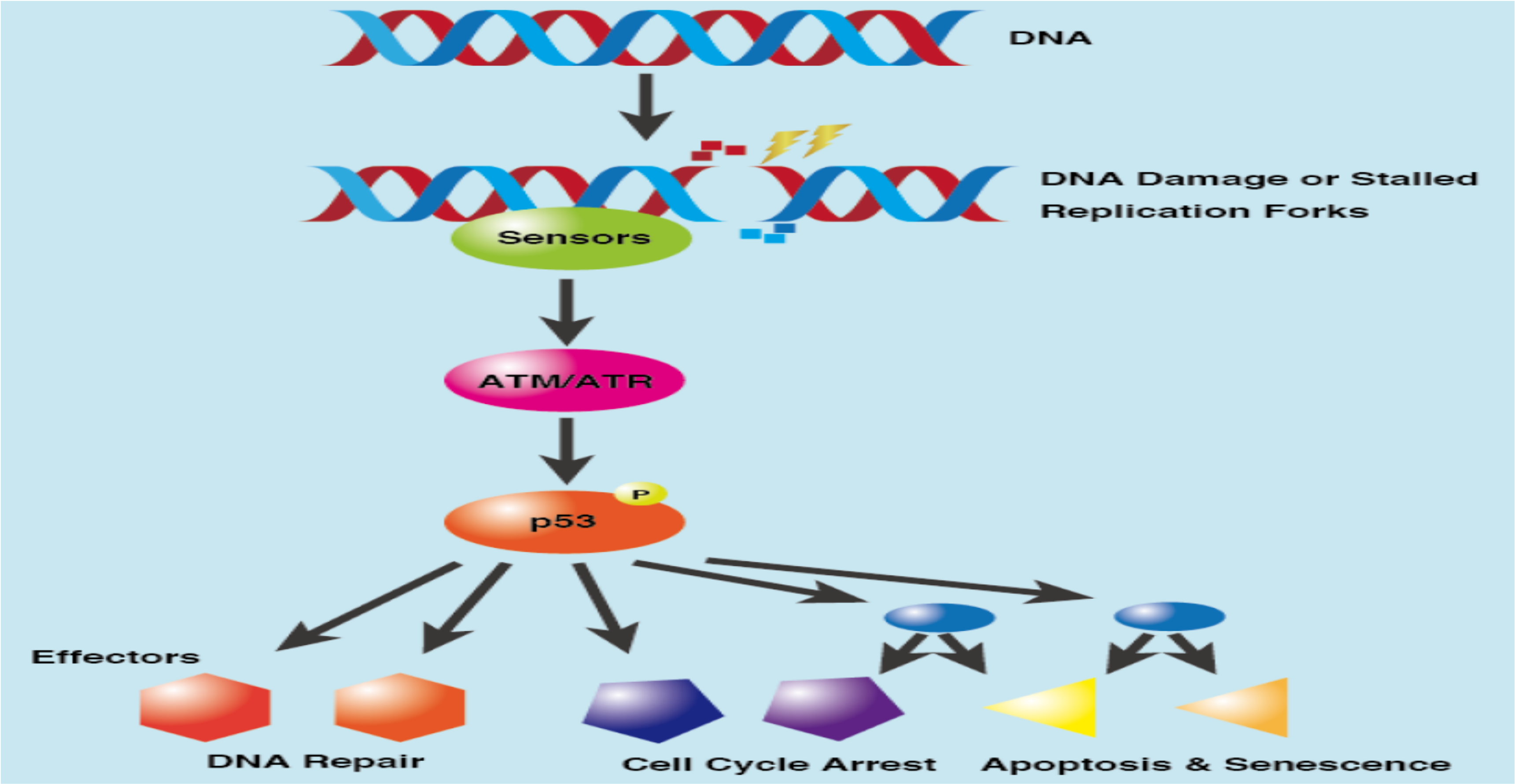

This is a picture of "Schematic representation of p53-mediated DNA Damage Response"

Please give a suitable interpretation of this picture

(Explanation from the original article)
Cellular DNA is constantly under attack by various endogenous metabolites, ionizing radiation, environmental and dietary carcinogens, as well as drugs such as genotoxic anti-cancer agents [39]. Such DNA damage includes base/sugar alterations, DNA-base mismatches, base adducts, sugar base cyclization, DNA protein crosslinking, or intra and inter-strand cross links, all of which result in DNA single- or double-strand breaks (SSBs and DSBs) [40]. While SSBs and other types of DNA damage are relatively less toxic, DSBs are quite dangerous, causing cell death, genomic instability, or carcinogenesis if left unrepaired [41]. DDR involves a broad signalling system that begins seconds after DNA damage and modulates various cellular processes in a concerted, structured manner. It is initiated by DNA damage sensors and signal transducers (ATM and ATR) and effectors (substrates of ATM and ATR) and induces cell cycle checkpoints, activates various DNA repair mechanisms, modulates gene expression, activity and turnover of a wide array of proteins, and thereby affects many aspects of cellular metabolism

BOT-1

This figure depicts the p53-mediated DNA Damage Response (DDR), a critical pathway in maintaining genomic stability and preventing the propagation of damaged DNA. Below is an interpretation of the key components:

Initial Event: DNA Damage

•DNA damage occurs due to factors such as replication fork stalling, double-strand breaks, or exposure to harmful agents (e.g., radiation, chemicals).

•The damage is depicted by breaks in the DNA double helix structure.

Detection of DNA Damage

1.Sensors:

- Specialized proteins detect DNA damage or stalled replication forks.
- These sensors initiate a signaling cascade to address the damage.

Signal Transduction via ATM/ATR

2.ATM/ATR Kinases:

- ATM (Ataxia Telangiectasia Mutated) and ATR (ATM and Rad3-Related) are key kinases activated by DNA damage.
- They phosphorylate downstream targets, including **p53**, to coordinate the cellular response.

Activation of p53

3.Phosphorylation of p53:

- p53, a tumor suppressor protein, is stabilized and activated through phosphorylation by ATM/ATR.
- Activated p53 acts as a transcription factor to regulate various downstream pathways.

Effector Functions Mediated by p53

4.DNA Repair:

- p53 activates genes involved in DNA repair mechanisms, ensuring the correction of damaged DNA.

5.Cell Cycle Arrest:

- p53 halts the cell cycle at checkpoints (e.g., G1/S, G2/M) to allow sufficient time for DNA repair.
- This prevents the replication of damaged DNA.

6.Apoptosis and Senescence:

- If the damage is irreparable, p53 triggers programmed cell death (apoptosis) or cellular senescence.
- Apoptosis eliminates severely damaged cells, while senescence prevents them from dividing further.

Conclusion

This schematic highlights the central role of p53 in maintaining genomic integrity through:

•**DNA repair** for damage correction.

•**Cell cycle arrest** to prevent propagation of errors.

•**Apoptosis or senescence** as a fail-safe mechanism against irreparable damage.

The pathway is crucial in tumor suppression, as mutations in p53 or associated proteins often result in genomic instability and cancer development. Understanding this pathway is essential for therapeutic approaches targeting DDR in cancer and other diseases.

BOT-2

This image provides a simplified view of how the p53 protein, a crucial tumor suppressor, responds to DNA damage within a cell. Here's a breakdown:

1. **DNA Damage:**
   - The image starts with a normal DNA molecule (represented by the red helix).
   - Then, it shows DNA damage or stalled replication forks (represented by the blue helix with breaks and lightning bolts). This damage can be caused by various factors like radiation, chemicals, or errors during DNA replication.
2. **Sensors:**
   - Specialized proteins act as sensors, detecting the DNA damage or replication problems. These sensors are not specifically named in the image but include proteins like ATM and ATR.
3. **ATM/ATR Activation:**
   - Once the sensors detect damage, they activate key proteins called ATM (Ataxia Telangiectasia Mutated) and ATR (ATM and Rad3-related). These proteins are central to initiating the DNA damage response.
4. **p53 Activation:**
   - ATM/ATR then activate p53 (shown as the orange circle). This activation often involves phosphorylation (adding a phosphate group - "P" in the image), which stabilizes and activates p53.
5. **p53 Effector Functions:**
   - Activated p53 acts as a transcription factor, meaning it can turn genes on or off. This leads to several crucial responses:
     - **DNA Repair:** p53 activates genes involved in DNA repair mechanisms, attempting to fix the damage (orange hexagon).
     - **Cell Cycle Arrest:** p53 can halt the cell cycle (purple pentagon), preventing the cell from dividing with damaged DNA. This gives the cell time to repair the damage or, if the damage is severe...
     - **Apoptosis & Senescence:** p53 can trigger programmed cell death (apoptosis - yellow triangles) or cellular senescence (permanent cell cycle arrest - yellow triangles), eliminating cells with irreparable DNA damage.

In Summary: This image effectively illustrates the central role of p53 in responding to DNA damage. It highlights the sequence of events from damage detection to the activation of various cellular responses aimed at preserving the integrity of the genome. p53 is often called the "guardian of the genome" due to its critical function in preventing the propagation of cells with damaged DNA, which could lead to cancer.

BOT-3

The image titled "Schematic representation of p53-mediated DNA Damage Response" illustrates the sequence of events that occur when DNA damage or stalled replication forks are detected in a cell.

Key Steps and Components:

1. **DNA Damage or Stalled Replication Forks**:
   - The process begins with the detection of DNA damage (depicted as breaks) or stalled replication forks.

2. **Sensors**:
   - These damages are recognized by sensor proteins, represented as a green oval labeled "Sensors."

3. **ATM/ATR Activation**:
   - The sensors activate ATM (Ataxia Telangiectasia Mutated) or ATR (ATM and Rad3-related) kinases, indicated by a pink oval labeled "ATM/ATR."

4. **p53 Activation**:
   - Activated ATM/ATR phosphorylates and activates the p53 protein, shown as an orange oval labeled "p53" with a phosphate group (P).

5. **Effector Pathways**:
  The activation of p53 leads to several downstream responses:
    - **DNA Repair**: Represented by pentagons.
    - **Cell Cycle Arrest**: Also indicated by pentagons.
    - **Apoptosis and Senescence**: Illustrated by triangles.

Overall Significance:
The diagram highlights the critical role of the p53 protein in maintaining genomic stability. When DNA damage is detected, p53 acts as a central regulator by either facilitating repair, halting cell division to prevent the spread of damaged DNA, or triggering programmed cell death (apoptosis) and cellular aging (senescence) to protect the organism from potential mutations that could lead to cancer. This intricate response ensures the integrity of the genetic material and the proper functioning of the cell.

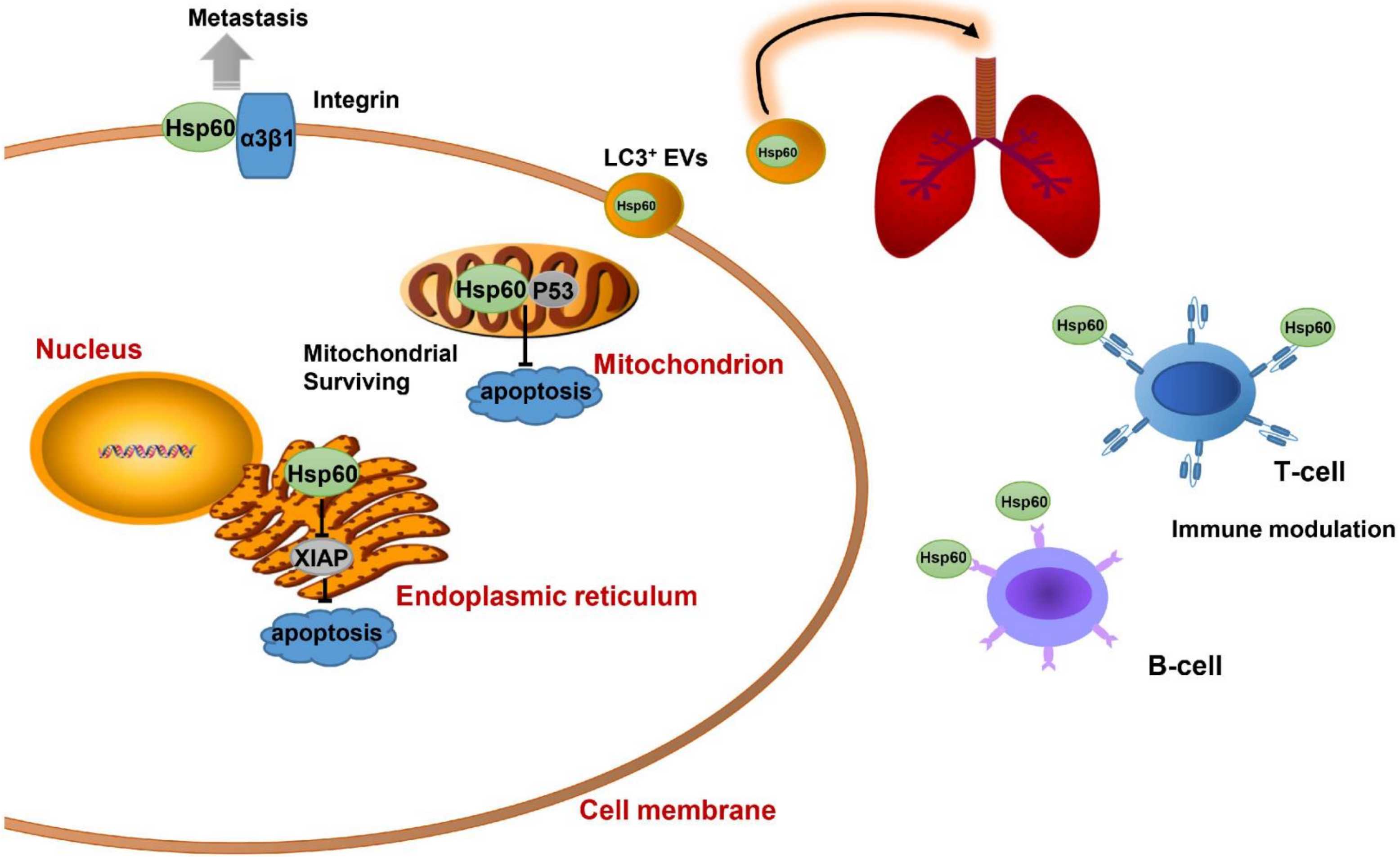

This is a picture of "A model illustrating Hsp60's regulation of breast cancer"

Please give a suitable interpretation of this picture

(Explanation from the original article)
Hsp60 in the endoplasmic reticulum inhibits XIAP expression to confer resistance to apoptosis. Mitochondrial Hsp60 interacts with P53 to suppress P53-dependent apoptosis. Hsp60 on the cell membrane interacts with α3β1-integrin to promote metastasis. Extracellular Hsp60 acts as an antigen for B-cells and T-cells to modulate the immune system in early stages of breast cancer. Exosomal Hsp60 in plasma accelerates the lung metastasis of breast cancer.

BOT-1

This figure illustrates the multifaceted role of **Hsp60 (Heat Shock Protein 60)** in regulating breast cancer progression and its interactions with various cellular compartments and immune cells. Below is a detailed interpretation:

Hsp60 at the Cellular and Subcellular Level

1.Cell Membrane:
- Hsp60 interacts with **Integrin α3β1** on the cell membrane, promoting **metastasis** by enhancing cell adhesion, migration, and invasion.

2.Mitochondrion:
- Hsp60 binds to **p53**, influencing mitochondrial pathways related to apoptosis.
- This interaction can support mitochondrial survival and inhibit apoptosis, favoring cancer cell persistence.

3.Endoplasmic Reticulum:
- Hsp60 interacts with **XIAP (X-linked inhibitor of apoptosis protein)** in the endoplasmic reticulum, inhibiting apoptosis and promoting cancer cell survival.

Hsp60 and Extracellular Vesicles (EVs)
- Hsp60 is released via **LC3-positive extracellular vesicles (LC3+ EVs)**, which can act as signaling molecules in the tumor microenvironment.
- These vesicles may contribute to metastasis or influence distant organ systems (e.g., the lungs in this model).

Hsp60 and Immune Modulation

1. T-Cells:
- Hsp60 modulates T-cell functions, possibly contributing to immune evasion or altering immune responses within the tumor microenvironment.

2. B-Cells:
- Similarly, Hsp60 influences B-cell activity, which may affect humoral immunity or immune surveillance in breast cancer.

Hsp60 and Metastasis
- By interacting with integrins and being secreted into the extracellular environment, Hsp60 promotes **cancer cell invasion** and supports the spread of breast cancer to distant organs (e.g., the lungs in this model).

Conclusion

Hsp60 serves as a regulator of breast cancer progression by:
- Supporting **cancer cell survival** through mitochondrial and endoplasmic reticulum interactions.
- Promoting **metastasis** via integrins and extracellular vesicles.
- Modulating the **immune system**, potentially suppressing anti-tumor immunity.

This model highlights the dual role of Hsp60 in intracellular survival mechanisms and extracellular communication, making it a potential target for breast cancer therapy.

BOT-2

This image depicts the diverse roles of Heat Shock Protein 60 (Hsp60) in breast cancer development and progression. Hsp60 is a chaperone protein, typically involved in protein folding and cellular stress responses. However, this illustration highlights its complex involvement in cancer:

1. Intracellular Roles:

•**Mitochondria:** Hsp60 can localize to the mitochondria, where it can have opposing effects:

- •**Mitochondrial survival:** It can promote cell survival by inhibiting apoptosis (programmed cell death).
- •**Mitochondrial apoptosis:** Conversely, it can also induce apoptosis under certain conditions.

•**Cytoplasm:** Hsp60 in the cytoplasm can inhibit XIAP (X-linked Inhibitor of Apoptosis Protein), a molecule that normally blocks apoptosis, potentially promoting cell death.

•**Nucleus:** Hsp60 can enter the nucleus and interact with p53, a crucial tumor suppressor protein. This interaction can influence cell survival and death pathways.

.

2. Extracellular Roles:

•**Secretion:** Hsp60 can be secreted from breast cancer cells, potentially through extracellular vesicles (EVs) marked with LC3 (a marker of autophagy).

•**Metastasis:** Extracellular Hsp60 may promote metastasis (spread of cancer) by interacting with integrin α3β1, a cell surface receptor involved in cell adhesion and migration. This interaction is depicted as contributing to the "Metastasis" arrow.

•**Immune Modulation:** Secreted Hsp60 can interact with immune cells, such as T cells and B cells, potentially modulating the immune response to the tumor. This interaction could either stimulate or suppress anti-tumor immunity.

3. Lung Metastasis:

•The image specifically highlights the lungs as a potential site of metastasis, indicating that breast cancer cells secreting Hsp60 may preferentially spread to the lungs.

In Summary: This image illustrates the multifaceted roles of Hsp60 in breast cancer. It shows how Hsp60 can influence cell survival, apoptosis, metastasis, and immune responses. This complexity suggests that Hsp60 could be a potential therapeutic target, but its diverse functions need to be carefully considered when designing interventions

BOT-3

The image you provided titled "A Model Illustrating Hsp60’s Regulation of Breast Cancer" offers a detailed view of the role Heat Shock Protein 60 (Hsp60) plays in breast cancer progression and its interaction with various cellular components.

Key Components and Interactions:

1. **Nucleus**:
   - Depicted with DNA inside, indicating the genetic material of the cell.

2. **Endoplasmic Reticulum (ER)**:
   - Hsp60 interacts with XIAP (X-linked inhibitor of apoptosis protein) in the ER, leading to apoptosis (programmed cell death).

3. **Mitochondrion**:
   - Hsp60 interacts with p53 within the mitochondrion, which also triggers apoptosis.
   - Hsp60 is involved in mitochondrial survival, ensuring proper mitochondrial function.

4. **Cell Membrane**:
   - Hsp60 interacts with integrin α3β1 on the cell membrane, which is associated with metastasis (the spread of cancer cells).

5. **LC3+ Extracellular Vesicles (EVs)**:
   - Hsp60 is present in LC3+ EVs, which are shown traveling to the lungs. These vesicles may play a role in the distant spread of cancer cells.

6. **Immune Modulation**:
   - Hsp60 is depicted on the surface of T-cells and B-cells, indicating its role in modulating the immune response.

Summary:
This schematic underscores the multifaceted role of Hsp60 in breast cancer. It highlights Hsp60's involvement in apoptosis through its interaction with XIAP and p53, its role in metastasis via integrin α3β1, and its presence in extracellular vesicles that may contribute to cancer spread. Furthermore, Hsp60's interaction with immune cells suggests it has a role in immune modulation. Understanding these pathways is crucial for developing potential therapeutic strategies targeting Hsp60 in breast cancer.

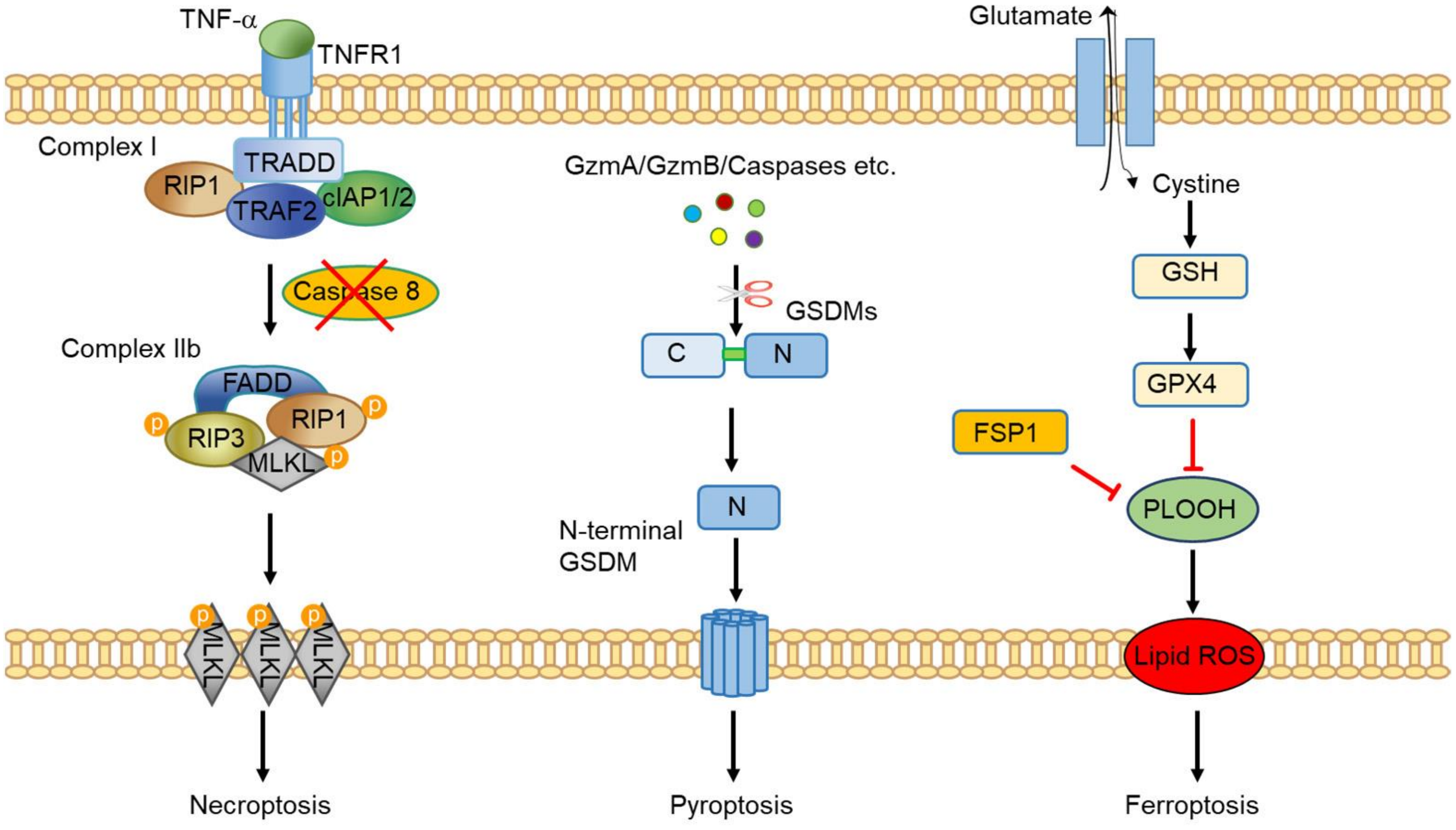

This is a picture of “Key non-apoptotic regulated cell death pathways"

Please give a suitable interpretation of this picture

(Explanation from the original article)

Figure 1. Key programmed necrosis pathways. The classical necroptotic pathway is activated by TNF stimulation. TNF binds to TNFR1 and leads to the formation of complex I. In the absence of caspase 8 activity, RIP1 interacts with RIPK3, FADD and MLKL to form complex IIb, which mediates necroptosis. Pyroptosis has been considered as gasdermin-mediated necrosis. Endogenous caspases and lymphocyte-derived Granzyme A/Granzyme B are able to cleave and activate individual gasdermins (GSDM) which further triggers pyroptosis. Ferroptosis is a failsafe rather than a typical cell death pathway. GPX4 and FSP1 prevent lipid peroxidation which is key to drive ferroptosis.

BOT-1

This figure provides an overview of three **non-apoptotic regulated cell death pathways**: necroptosis, pyroptosis, and ferroptosis. These pathways are alternative forms of programmed cell death that differ mechanistically from apoptosis. Here's an interpretation of the key components:

1. Necroptosis

A. **Trigger**: Necroptosis begins with the activation of **TNFR1 (Tumor Necrosis Factor Receptor 1)** by **TNF-α**.

B. **Signaling Cascade**:
   - Upon TNF-α binding, **Complex I** is formed, comprising **TRADD, RIP1, TRAF2**, and **cIAP1/2**.
   - When **caspase-8 is inactive or inhibited**, the signaling shifts to **Complex IIb**, which includes **FADD, RIP1, RIP3**, and **MLKL**.
   - **MLKL phosphorylation** by RIP3 leads to its oligomerization and insertion into the plasma membrane, causing membrane rupture and necroptosis.

C. **Outcome**: Necroptosis results in cell lysis, inflammation, and tissue damage.

2. Pyroptosis

A. **Trigger**: Pyroptosis is mediated by **gasdermins (GSDMs)** and is initiated by activation of inflammatory caspases (e.g., caspase-1, caspase-4/5/11) or granzymes (e.g., GzmA, GzmB).

B. **Signaling Cascade**:
   - GSDMs are cleaved into N-terminal and C-terminal fragments.
   - The **N-terminal GSDM** fragment oligomerizes to form pores in the plasma membrane.

C. **Outcome**: The formation of membrane pores leads to cell swelling, membrane rupture, and the release of pro-inflammatory cytokines, resulting in **pyroptosis**.

3. Ferroptosis

A. **Trigger**: Ferroptosis is driven by the accumulation of **lipid peroxides (PLOOH)** and **lipid ROS (Reactive Oxygen Species)**.

B. **Key Regulators**:
   - **Cystine uptake** via the glutamate-cystine antiporter provides cysteine for glutathione (GSH) synthesis.
   - **GPX4 (Glutathione Peroxidase 4)** detoxifies lipid peroxides, preventing ferroptosis.
   - **FSP1** (Ferroptosis Suppressor Protein 1) provides an alternative mechanism to inhibit lipid peroxidation.

C. **Outcome**: When GPX4 or FSP1 activity is insufficient, lipid ROS accumulates, leading to **ferroptosis**, characterized by oxidative membrane damage.

Comparison of the Pathways

•**Necroptosis**: Involves RIP1/RIP3/MLKL and results in inflammatory lysis.
•**Pyroptosis**: Driven by gasdermins and inflammatory caspases, leading to pore formation and cytokine release.
•**Ferroptosis**: Triggered by lipid peroxidation due to GPX4 or FSP1 failure, resulting in oxidative membrane damage.

Therapeutic Implications

•These pathways are important in various diseases:
   - **Necroptosis and Pyroptosis**: Linked to inflammatory disorders.
   - **Ferroptosis**: Explored as a therapeutic target in cancer, neurodegenerative diseases, and ischemia-reperfusion injury.

This schematic highlights the distinct molecular mechanisms and potential therapeutic targets for each non-apoptotic regulated cell death pathway.

BOT-2

This image illustrates three important non-apoptotic regulated cell death (RCD) pathways: necroptosis, pyroptosis, and ferroptosis. These are distinct mechanisms by which cells can undergo programmed death, differing from the more well-known process of apoptosis.

1. Necroptosis:

- **Triggered by:** TNF-α (tumor necrosis factor-alpha) binding to its receptor TNFR1.
- **Key players:** RIP1, TRADD, TRAF2, CIAP1/2, caspase 8, FADD, RIP3, and MLKL.
- **Mechanism:**
    - TNF-α binding initiates the formation of Complex I, which normally leads to apoptosis through caspase 8 activation.
    - However, if caspase 8 is inhibited (indicated by the red cross), Complex IIb forms, leading to RIP3 activation and phosphorylation of MLKL.
    - Phosphorylated MLKL then oligomerizes (forms clusters) and translocates to the cell membrane, disrupting its integrity and causing necroptosis.

2. Pyroptosis:

- **Triggered by:** Various stimuli, including pathogen-associated molecular patterns (PAMPs) and damage-associated molecular patterns (DAMPs).
- **Key players:** Caspases (e.g., caspase 1), GSDMs (Gasdermin family proteins).
- **Mechanism:**
    - Activation of caspases leads to cleavage of GSDMs, releasing their N-terminal domains.
    - These N-terminal domains form pores in the cell membrane, disrupting cellular integrity and leading to pyroptosis.
    - This process often involves the release of inflammatory cytokines.

3. Ferroptosis:

- **Triggered by:** Accumulation of lipid peroxides (PLOOH).
- **Key players:** GPX4 (glutathione peroxidase 4), GSH (glutathione), FSP1 (ferroptosis suppressor protein 1), cystine.
- **Mechanism:**
    - GPX4, a crucial enzyme that reduces lipid peroxides, requires glutathione for its activity.
    - Depletion of glutathione or inhibition of GPX4 (indicated by the red arrow blocking GPX4) leads to the accumulation of lipid peroxides.
    - This causes membrane damage and ultimately ferroptosis.
    - FSP1 can act as an alternative system to reduce lipid peroxides, offering some protection against ferroptosis.

In Summary: This image provides a concise overview of three distinct non-apoptotic RCD pathways, highlighting their key triggers, molecular players, and mechanisms. Understanding these pathways is crucial for comprehending various physiological and pathological processes, including inflammation, infection, and cancer.

BOT-3

The image illustrates the key non-apoptotic regulated cell death pathways, highlighting necroptosis, pyroptosis, and ferroptosis. Here's a breakdown of each pathway:

1. Necroptosis Pathway:
   - Initiation: Triggered by TNF-α binding to TNFR1.
   - Formation of Complex I: Involves TRADD, RIP1, TRAF2, and cIAP1/2.
   - Formation of Complex IIb: Occurs upon inhibition of Caspase 8 and involves FADD, RIP1, RIP3, and MLKL.
   - Execution: Phosphorylation of MLKL, leading to its translocation to the plasma membrane, causing cell membrane disruption and necroptosis.

2. Pyroptosis Pathway:
   - Initiation: Involves the cleavage of Gasdermin proteins (GSDMs) by enzymes such as GzmA, GzmB, and caspases.
   - Execution**: Cleavage releases the N-terminal domain of GSDMs, which form pores in the plasma membrane, leading to cell swelling, lysis, and pyroptosis.

3. Ferroptosis Pathway:
   - Cystine Import and Conversion: Cystine is imported and converted to GSH.
   - Role of GPX4: GSH is utilized by GPX4 to reduce lipid hydroperoxides (PLOOH) to non-toxic lipid alcohols.
   - Inhibition of GPX4: Leads to the accumulation of lipid ROS (Reactive Oxygen Species), causing ferroptosis.
   - FSP1: Inhibits the accumulation of lipid ROS, preventing ferroptosis.

Overall Significance:
This image is crucial for understanding how cells undergo non-apoptotic regulated cell death, which differs from the traditional apoptosis pathway. These pathways play significant roles in various physiological and pathological processes, including inflammation, cancer, and neurodegenerative diseases.
