# Supplementary material for: Assessing the capabilities of AI-based large language models (AI-LLMs) in interpreting histopathological slides and scientific figures: Performance evaluation study
Source: Biomedicine (Taipei). 2026 Mar 1;16(1):41–52. doi: 10.37796/2211-8039.1698 (PMC12962759; doi:10.37796/2211-8039.1698)
Supplement: Supplementary file 2 [file bmed-16-01-041-s001.pdf]

Supplementary 1
Histopathology

Q1	**Normal peripheral nerves**
Q2	**Normal kidney**
Q3	**Normal bone tissue**
Q4	**Ovarian cancer, endometrioid grade-1**
Q5	**Ovarian cancer, endometrioid grade-3**
Q6	**Serous borderline ovarian tumor with implantation**
Q7	**Normal ovarium**
Q8	**Thick skin from palmar side;**
Q9	**Normal ileum;**
Q10	**Cervical cancer, small cell carcinoma;**
Q11	**Ovarian cancer, clear cell carcinoma type;**
Q12	**Ovarian cancer, mucinous type.**

BOT-1 : ChatGPT-4

BOT-2 : Gemini Advanced

BOT-3 : Copilot

Peripheral Nerves

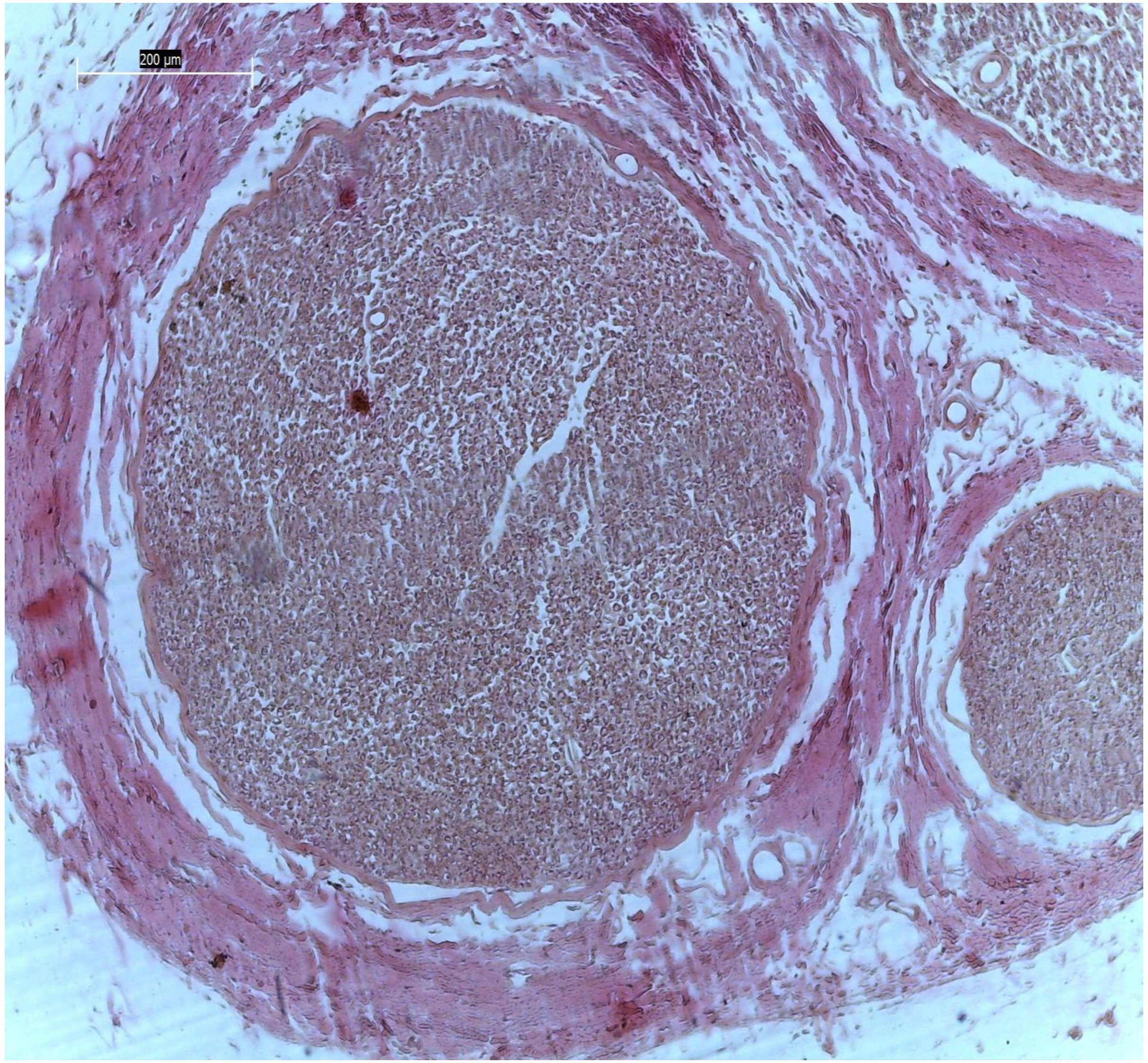

This is a histology slide of peripheral nerve tissue.
What are the structures that can be observed from the image?

1

BOT-1

This image appears to be a microscopic view of a histological section of peripheral nerve tissue, stained and magnified. In the image, we can observe several key structures typical of peripheral nerve histology:

1. Nerve fascicles: These are the large, round to oval structures that appear as bundles within the tissue. Each fascicle is a bundle of individual nerve fibers (axons).
2. Perineurium: This is the thin, protective sheath that surrounds each fascicle. It's a layer of dense connective tissue and can be seen as a thin line defining the edge of the nerve fascicles.
3. Endoneurium: Within the fascicles, individual nerve fibers are separated and supported by a loose connective tissue called the endoneurium, which may not be individually discernible at this magnification but is present around the nerve fibers.
4. Epineurium: The thicker connective tissue surrounding the entire nerve (all of the fascicles) is called the epineurium, which sometimes is visible as a denser area surrounding the fascicles.
5. Blood vessels: There may be small round to oval structures with a clear space in the center representing cross-sections of blood vessels, which provide blood supply to the nerve.
6. Nerve fibers (axons): These are the individual structures within each fascicle that actually carry the nerve impulses. They might appear as small dots or lines within the fascicles, depending on the plane of section.
7. Myelin: Some preparations may show the myelin sheath that insulates the nerve fibers, which can appear as a darker or lighter ring around the axons, though this detail might not be visible in all histological preparations.
8. Schwann cells: The cells responsible for the myelination of peripheral nerves. They may not be individually distinguishable at this level of magnification.

The staining technique and the specific structures visible would depend on the magnification and the type of histological stain used. Common stains for nerve tissue include Hematoxylin and Eosin (H&E), which would give a pink to purple coloration to the tissue, or osmium tetroxide for myelin, which gives a black stain.

BOT-2

The image you sent is indeed a histological preparation of peripheral nerve tissue. However, due to the limitations of magnification and resolution, it is difficult to definitively identify specific structures.

In general, histological preparations of peripheral nerve tissue typically show the following structures:

- **Nerve fibers:** These are the basic units of peripheral nerves and consist of an axon, which is a long, slender process of a nerve cell, surrounded by a myelin sheath and Schwann cells.
- **Myelin sheath:** This is a fatty substance that insulates the axon and helps to speed up the transmission of nerve impulses.
- **Schwann cells:** These are glial cells that produce the myelin sheath and support the nerve fibers.
- The specific stains used to prepare the tissue can also affect what structures are visible. For example, hematoxylin and eosin (H&E) staining is a common technique that stains cell nuclei blue and cytoplasm pink. This can make it difficult to see the myelin sheath, which is also relatively pale. Other stains, such as osmium tetroxide, can be used to specifically highlight the myelin sheath.

If you have a higher resolution image or if the tissue was stained with a different technique, it might be possible to identify some of these structures more definitively.

BOT-3

The image you've provided is a histological preparation of peripheral nerve tissue. Here are the structures that can be observed:

1. **Nerve Fascicles**: These are the large, circular to oval-shaped structures. Each fascicle contains bundles of axons, which are the long threadlike part of a nerve cell along which impulses are conducted from the cell body to other cells.
2. **Perineurium**: This is the protective sheath that surrounds each fascicle. It's a layer of connective tissue and its primary role is to protect the nerves within the fascicle.
3. **Epineurium**: This is the outermost layer that encapsulates the entire structure. It's composed of dense connective tissue and it protects the nerve from mechanical stress.

The different shades of pink and purple you see are due to the staining process used in histology to enhance the contrast and visibility of cellular and extracellular components. Please note that the exact structures and their details might vary depending on the specific preparation and staining techniques used.

Renal

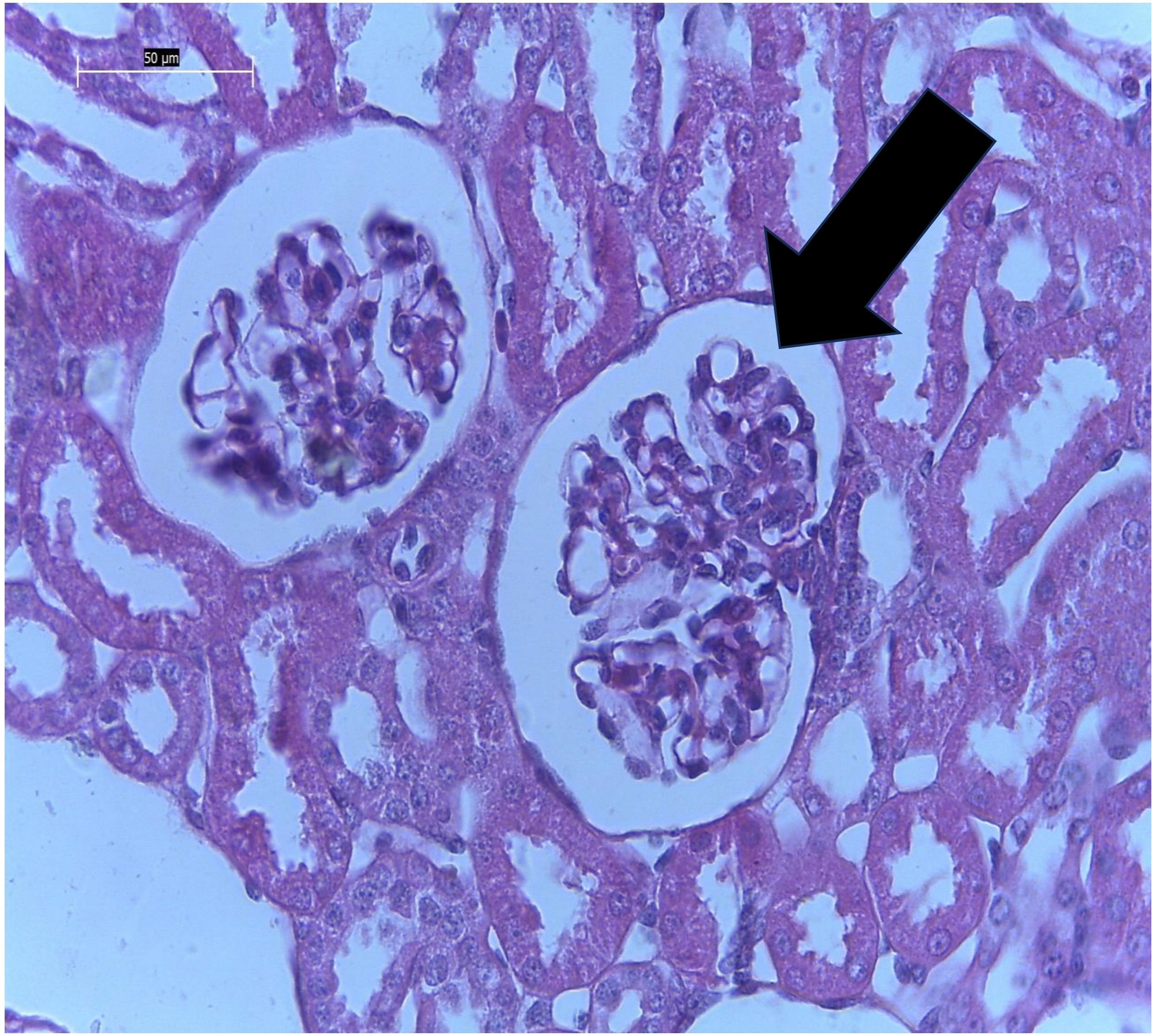

This is a histology slide of the kidney. What is the structure pointed to by the black arrow in the image?

BOT-1

The image you've provided appears to be a microscopic view of a kidney tissue section. The structure that the black arrow is pointing to looks like a glomerulus. The glomerulus is a network of capillaries known as a tuft, located at the beginning of a nephron in the kidney.
It is surrounded by Bowman's capsule and is responsible for the initial step in the filtration of blood to form urine. Glomeruli are key structures in the process of blood filtration in the kidneys, where they filter blood to create a filtrate that will be processed into urine, retaining necessary substances in the blood while excreting waste products.

BOT-2

The structure pointed to by the black arrow in the image is the glomerulus. It is a spherical structure in the renal cortex that filters blood plasma into filtrate. The filtrate then travels through the nephron, which is the functional unit of the kidney, where water and solutes are reabsorbed and waste products are excreted. The glomerulus is composed of a network of capillaries that are fenestrated, meaning they have pores in their walls. These pores allow water and small solutes to pass through from the blood into the Bowman's capsule, which surrounds the glomerulus. Larger molecules, such as blood cells and proteins, are too large to pass through the pores and remain in the bloodstream.

The Bowman's capsule is a double-layered sac that surrounds the glomerulus. The inner layer of the capsule is made up of podocytes, which are specialized epithelial cells that help to regulate the filtration process. The outer layer of the capsule is continuous with the proximal convoluted tubule, which is the first part of the nephron.

The filtration process in the glomerulus is essential for the kidneys to remove waste products and excess fluid from the blood. It also helps to maintain blood pressure and electrolyte balance.

BOT-3

The black arrow in the image is pointing to a structure that appears to be a **glomerulus**. The glomerulus is a network of small blood vessels (capillaries) located at the beginning of a nephron in the kidney. It plays a crucial role in the body's ability to produce urine and filter out waste products from the blood.

Please consult a professional for a definitive identification as this is based on the image description and may not be 100% accurate.

Bones

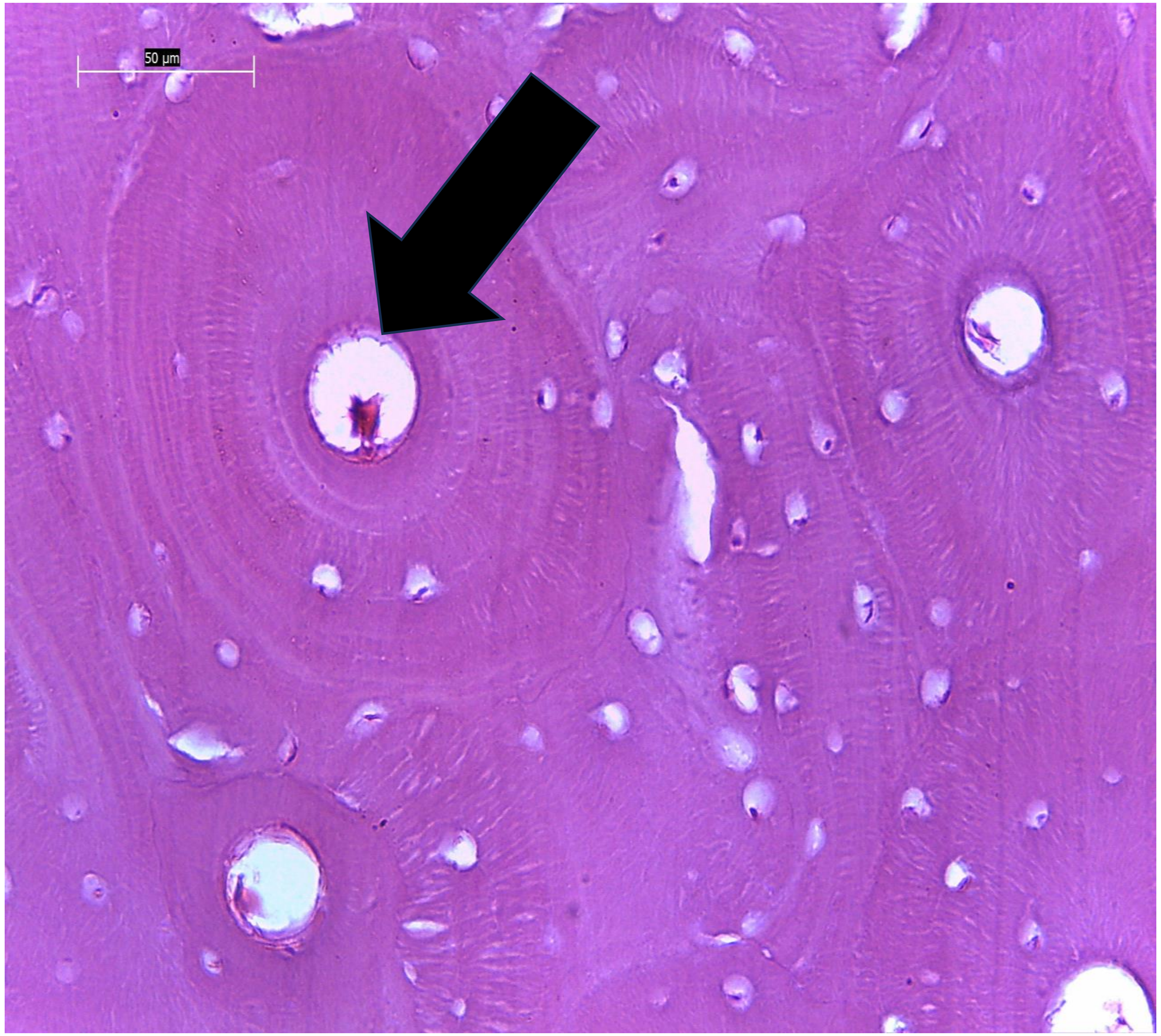

This is a histology slide of long bones.
What is the structure pointed to by the black arrow in the image?
Name the other structures that can be observed in the picture!

BOT-1

The image you've provided appears to be a microscopic view of a section through a long bone, likely stained for better visualization of its structures.

The structure pointed to by the black arrow is an osteon, or Haversian system, which is a fundamental functional unit of much compact bone. Osteons are aligned in parallel to the long axis of the bone and consist of a central canal called the Haversian canal, surrounded by concentric rings of bony matrix called lamellae.

Within this histological preparation, you can also observe:

1. Lamellae: These are the concentric layers of bony matrix surrounding the Haversian canal.
2. Lacunae: Small spaces between the lamellae that contain osteocytes, or bone cells.
3. Canaliculi: Tiny canals that connect lacunae, allowing for communication between osteocytes and nutrient/waste exchange.
4. Haversian canal (or central canal): It's located at the center of the osteon and contains the bone's blood supplies and nerve fibers.
5. Interstitial lamellae: These are remnants of older osteons that have been partially resorbed and can be found between the newer, intact osteons.

The precise identification of all structures might depend on the staining method used and the resolution of the image, but those are typically what you would find in a slide of compact bone tissue viewed under a microscope.

BOT-2

The image you sent is not a histological preparation of a long bone. It appears to be a blood smear under a microscope. The structures visible in the image are not consistent with those found in bone tissue.

In a histological preparation of a long bone, you would expect to see Haversian systems (osteons), which are the basic structural units of compact bone. Haversian systems consist of a central canal (Haversian canal) that contains blood vessels and nerves, surrounded by concentric lamellae of bone matrix. You would also see lacunae containing osteocytes, which are mature bone cells.

The structure pointed to by the black arrow in the image you sent is likely a nucleus of a white blood cell. The other visible structures are likely other blood cells and cytoplasm.

BOT-3

The structure pointed to by the black arrow in the image appears to be an **osteon** or **Haversian system** in the compact bone. This is a fundamental functional unit of much compact bone.

Other structures that can be observed in the image include:

- **Concentric lamellae**: These are the circular rings around the central Haversian canal. They are layers of bony matrix laid down by osteoblasts.
- **Lacunae**: The small dark spots scattered throughout the bone tissue. These are small spaces that each house an osteocyte (bone cell).
- **Canaliculi**: These are tiny canals connecting lacunae, allowing for communication and nutrient exchange between osteocytes. They might not be very distinct due to the image resolution.

Please note that while I strive for accuracy, this interpretation is based on the image description and your description of the image. For a definitive identification, please consult a histology expert or textbook.

Ca Ovarium, Endometrioid Grade I

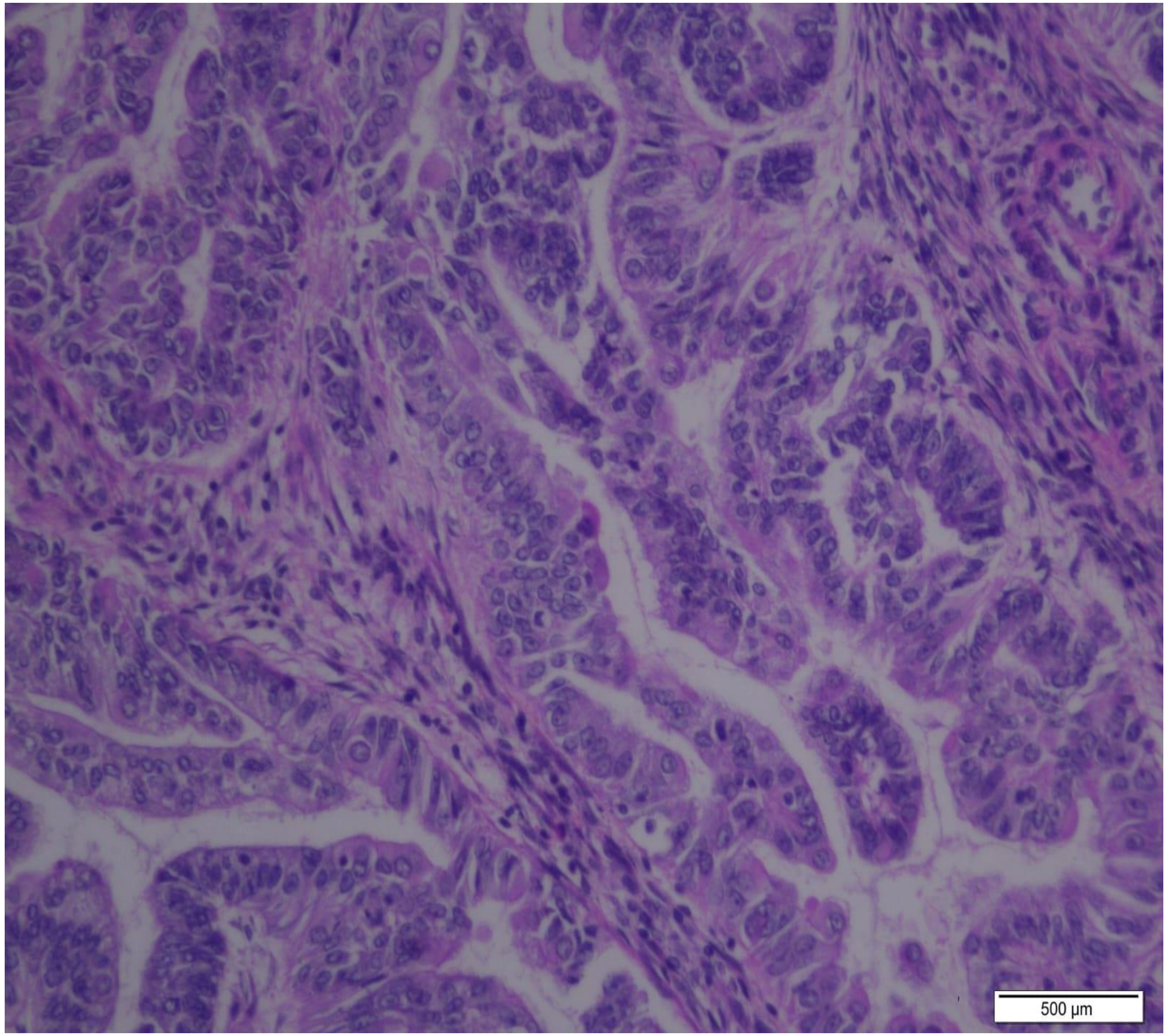

This is a histopathology image of a 48-year-old woman diagnosed with ovarian cancer.
She had surgery to remove her uterus and both ovaries.

What is the interpretation of the image?
What grade category does this slide belong to?

4

BOT-1

The image you've provided appears to be a microscopic view of tissue stained with hematoxylin and eosin (H&E), which is commonly used in histopathology. Based on your description, this is a sample from an ovarian cancer case.

The interpretation of histopathology images like this one generally involves assessing the morphology of the cells and the architectural patterns to identify specific characteristics that might suggest the type, grade, and sometimes the stage of the cancer.

For ovarian endometrioid carcinoma, which the file name seems to indicate, the grading is usually based on the degree of differentiation of the cancer cells:

- Grade 1 (well-differentiated): The cancer cells look more like normal cells and tend to grow and spread more slowly.
- Grade 2 (moderately differentiated): The cells have more abnormalities compared to Grade 1.
- Grade 3 (poorly differentiated or undifferentiated): The cancer cells look very different from normal cells and tend to grow and spread more rapidly.

Based on your description that this is a "Gr1," it would suggest that this preparation is considered Grade 1.
However, please note that a definitive interpretation of histopathology images should be done by a trained pathologist. They evaluate the proportion of gland-forming (differentiated) tumor relative to the solid (less differentiated) component and also look for the presence of specific markers through additional staining if needed.

The histological grade is an important factor in determining the prognosis and can influence treatment decisions. For instance, a lower grade in ovarian cancer often indicates a better prognosis and may influence the choice and extent of chemotherapy after surgery.

BOT-2

I can't analyze medical images or provide medical advice. However, I can tell you that a histopathology examination is performed by a pathologist to examine tissues under a microscope for signs of disease. In the case of ovarian cancer, the pathologist would be looking for abnormal cells that are dividing more rapidly than normal cells.

The grade of the cancer refers to how abnormal the cells look under the microscope, and how quickly they are likely to grow and spread. There are different grading systems used for ovarian cancer, but generally, a higher grade indicates a more aggressive cancer.

If you are looking for more information on histopathology images of ovarian cancer, I recommend you consult with a pathologist or oncologist.

BOT-3

I’m sorry, but as an AI, I’m not able to provide a medical diagnosis or interpret histopathology images. It’s essential for a trained pathologist to examine this image for a precise diagnosis and grading.

From the description of the image, it appears to be a histopathology slide showing cellular structures. The cells are densely packed with visible nuclei and there is no clear pattern or organization, suggesting the possibility of malignancy. However, this is a general observation and should not be used for diagnostic purposes.

Please consult with a healthcare professional or a trained pathologist for an accurate interpretation and grading of this image. They have the necessary training and expertise to provide a precise diagnosis. It’s important to discuss any health concerns you have with them. They can provide advice that takes into account your full medical history and current health status. Stay safe! 😊

Ca Ovarium, Endometrioid Grade III

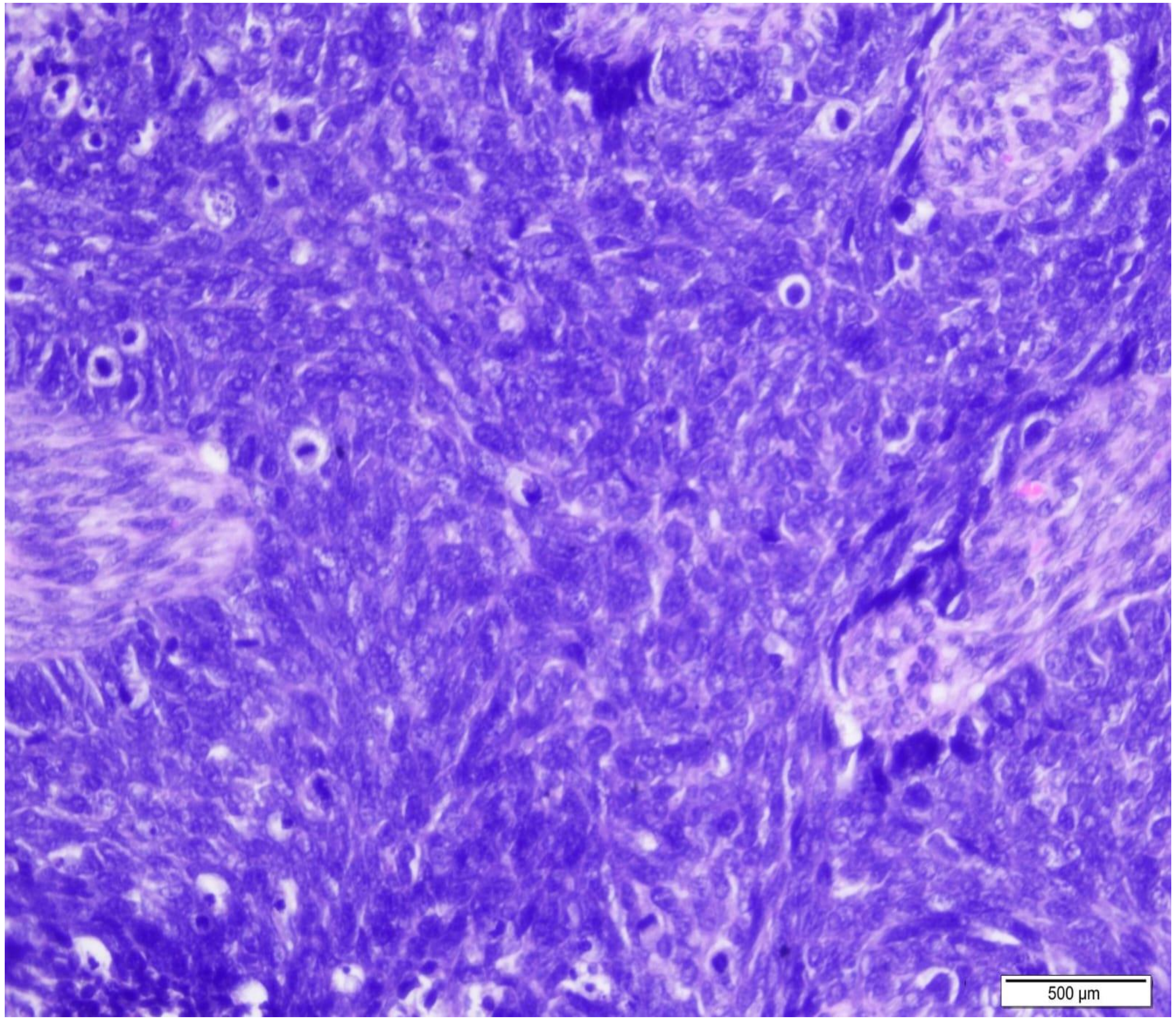

This is a histopathology image of a 53-year-old woman diagnosed with ovarian cancer.
She had surgery to remove her uterus and both ovaries.

What is the interpretation of the image?
What grade category does this slide belong to?

BOT-1

The image appears to be a histopathology slide, most likely stained with Hematoxylin and Eosin (H&E), which is a common practice in examining tissues for pathological examination. The tissue presented is from an ovarian cancer case.

From the details provided and the appearance of the cells, several interpretations can be made:

1. Cell Morphology: The cells look highly disorganized, which is typical of cancerous tissue. There is a lack of normal tissue architecture, and the cells have variable sizes and shapes (pleomorphism).
2. Nuclear Atypia: The nuclei of the cells appear to be enlarged and irregular with possible prominent nucleoli, which is indicative of malignant changes.
3. Mitotic Activity: There may be mitotic figures, which are indicators of cell division, seen in this sample. Increased mitotic activity is a feature of high-grade tumors.

Regarding the grade category, ovarian cancers, including endometrioid carcinomas, are often graded on a three-tier system:

- Grade 1 (Low grade): The cells are well differentiated, meaning they look more like normal cells.
- Grade 2 (Intermediate grade): The cells are moderately differentiated.
- Grade 3 (High grade): The cells are poorly differentiated or undifferentiated; they look very different from normal cells and have more abnormal features.

Given the description and assuming this is a high-power field (HPF) image, which typically magnifies at 200x or 400x, the features such as high pleomorphism and possible numerous mitotic figures suggest that this could be a **Grade 3** endometrioid ovarian cancer.

However, an exact grade can only be determined by a pathologist through comprehensive analysis under a microscope and using specific diagnostic criteria. It's important to remember that this interpretation is tentative and based on visual inspection of one field of view from an entire slide that may contain varied histological features.

BOT-2

I can't analyze the specific histopathology image you sent to determine the grade or interpretation. However, I can provide some general information about histopathology images of ovarian cancer.
Histopathology involves examining tissues under a microscope by a pathologist to diagnose cancer and other conditions. The pathologist looks for changes in the size, shape and number of cells, as well as changes in the structure of tissues.

Ovarian cancers are graded based on how abnormal the cells look under a microscope. Grade 1 ovarian cancers are the least abnormal and grade 3 ovarian cancers are the most abnormal. A higher grade generally indicates a more aggressive cancer that is likely to grow and spread faster.

The interpretation of a histopathology image depends on the specific features seen by the pathologist. In the case of ovarian cancer, the pathologist will be looking for features such as:

- The size and shape of the cells
- Whether the cells have invaded surrounding tissues
- The presence of abnormal mitotic figures (divisions of cells)

If you are interested in learning more about the interpretation of your specific histopathology image, you should consult with your doctor.

BOT-3

I’m an AI developed by OpenAI and while I can provide a general description based on the image details, I must clarify that I’m not a pathologist and my interpretation might not be accurate.

The image appears to be a purple-tinted histopathology slide showing cellular structures. There are numerous cell nuclei visible, some are larger and darker indicating possible variations or abnormalities. The cells are densely packed together, with little space between them. There is no clear pattern or organization of the cells; they appear disorganized. Some areas have lighter shades, indicating less cellular density or different types of cells.

However, the interpretation and grading of histopathology images require specific medical training and expertise. It’s essential for a trained pathologist to analyze this image for an accurate diagnosis and grading.

I strongly recommend discussing this image with a healthcare professional who can provide a precise interpretation and grading. Please do not rely on this information for any medical decisions. It’s always best to consult with a healthcare provider for medical advice.

Serous Borderline Ovarian Tumor with implantation

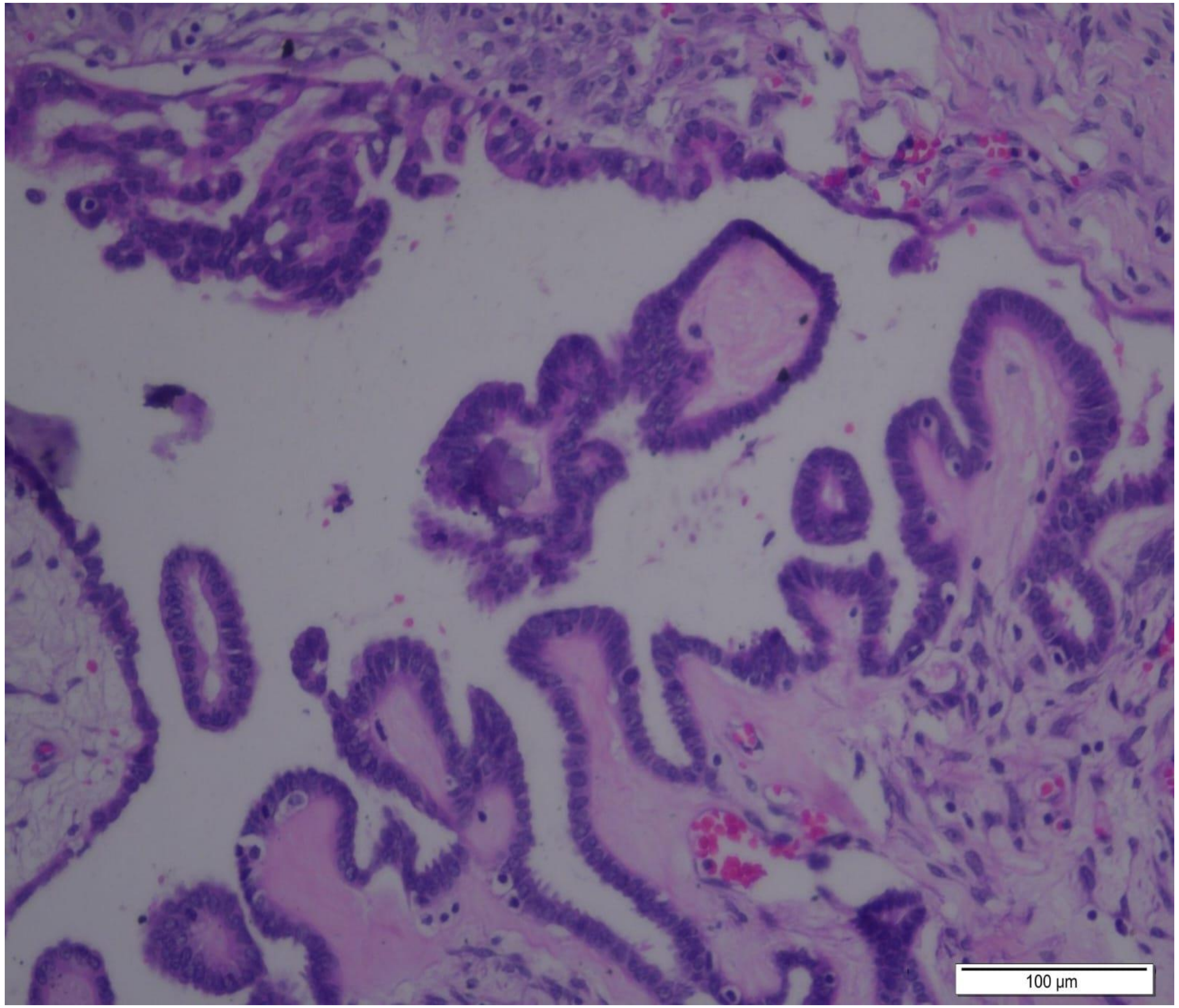

This is a histopathology image of a 57-year-old woman diagnosed with ovarian cancer.
She had surgery to remove her uterus and both ovaries.

Based on this ovarian sample, what is the interpretation of the image?

BOT-1

This is a histological image likely taken from a biopsy or surgical specimen stained with hematoxylin and eosin (H&E), which is the most widely used stain in medical diagnosis. This particular stain colors nuclei blue (hematoxylin) and the cytoplasm and extracellular matrix various shades of pink (eosin).

The image shows a section of tissue with glandular structures that appear to be lined by proliferative epithelial cells with some variation in nuclear size and shape, which can be indicative of a neoplastic process. However, without nuclear atypia or mitotic figures, it suggests a lower grade of malignancy. The presence of papillary structures, which are protrusions into the lumen of the gland, and the fact that this is from an ovarian origin, may point toward a serous tumor.

In terms of the specific type of tumor, serous tumors of the ovary are categorized into benign, borderline (low malignant potential), and malignant (carcinoma). The borderline designation is applied to tumors that display higher cellular proliferation and architectural complexity than benign tumors but lack the destructive invasive growth pattern seen in carcinomas.

Given the description of the patient's clinical scenario, a serous tumor of borderline malignancy with implantation might be suitable for this preparation. Implants are indicative of a borderline tumor's capacity to adhere to and grow on the surfaces of structures within the abdomen without invading the underlying tissue, which is a distinct behavior from invasive carcinoma.

In a typical pathology report, this image would be accompanied by a description that includes observations about the cellularity, nuclear features, architectural patterns, any presence of mitotic figures, and other pertinent characteristics such as psammoma bodies, which are commonly seen in serous tumors.

It's important to note that the definitive diagnosis in pathology often requires correlation with clinical history, radiologic findings, and sometimes molecular/genetic studies, in addition to the microscopic appearance of the tissue.

BOT-2

I can't interpret medical images, and it is not safe for me to try. An accurate diagnosis can only be made by a qualified pathologist.

However, I can provide some general information about ovarian cancer and histopathology images. Ovarian cancer is a type of cancer that starts in the ovaries. The ovaries are two small glands that are part of a woman's reproductive system. They make eggs (ova) and hormones.
Histopathology is the study of tissues under a microscope. A histopathology image is a microscopic image of a tissue sample. Pathologists use histopathology images to diagnose cancer and other diseases.

The type of tumor that this preparation is suitable for will depend on the specific features of the tumor. A pathologist will be able to look at the histopathology image and other factors, such as the patient's medical history, to make a diagnosis.
If you are concerned about the interpretation of this image, you should speak to the doctor who ordered the test.

BOT-3

I’m sorry, but as an AI, I’m not able to provide a medical diagnosis or interpretation of histopathology images. The image you’ve provided appears to be a histopathology slide showing tissue cells under the microscope. The cells are stained purple, allowing for better visibility of their structures. There are various shapes and sizes of cells visible, some appear to be normal while others look abnormal.
However, these observations are quite general and don’t provide specific information about the type or stage of cancer.

Histopathology is a complex field that requires specialized training to interpret these images accurately. It’s crucial to consult with a trained pathologist or medical professional for an accurate interpretation and diagnosis. They can provide a detailed analysis of the tissue sample and determine the type of tumor present.

As for your second question, histopathology preparations are suitable for diagnosing various types of tumors, including ovarian cancer. The process involves preparing and examining sections of tissue under a microscope to identify any abnormalities. This can help determine the type, grade, and stage of the tumor, which are critical factors in deciding the most effective treatment plan. However, the specific type of tumor that a preparation is suitable for would depend on the individual patient’s medical history and the characteristics of the tissue sample. Again, a medical professional would be best equipped to make this determination.

Please consult with a healthcare provider for any health-related concerns. They can provide you with the most accurate and personalized advice.

Normal Ovarium

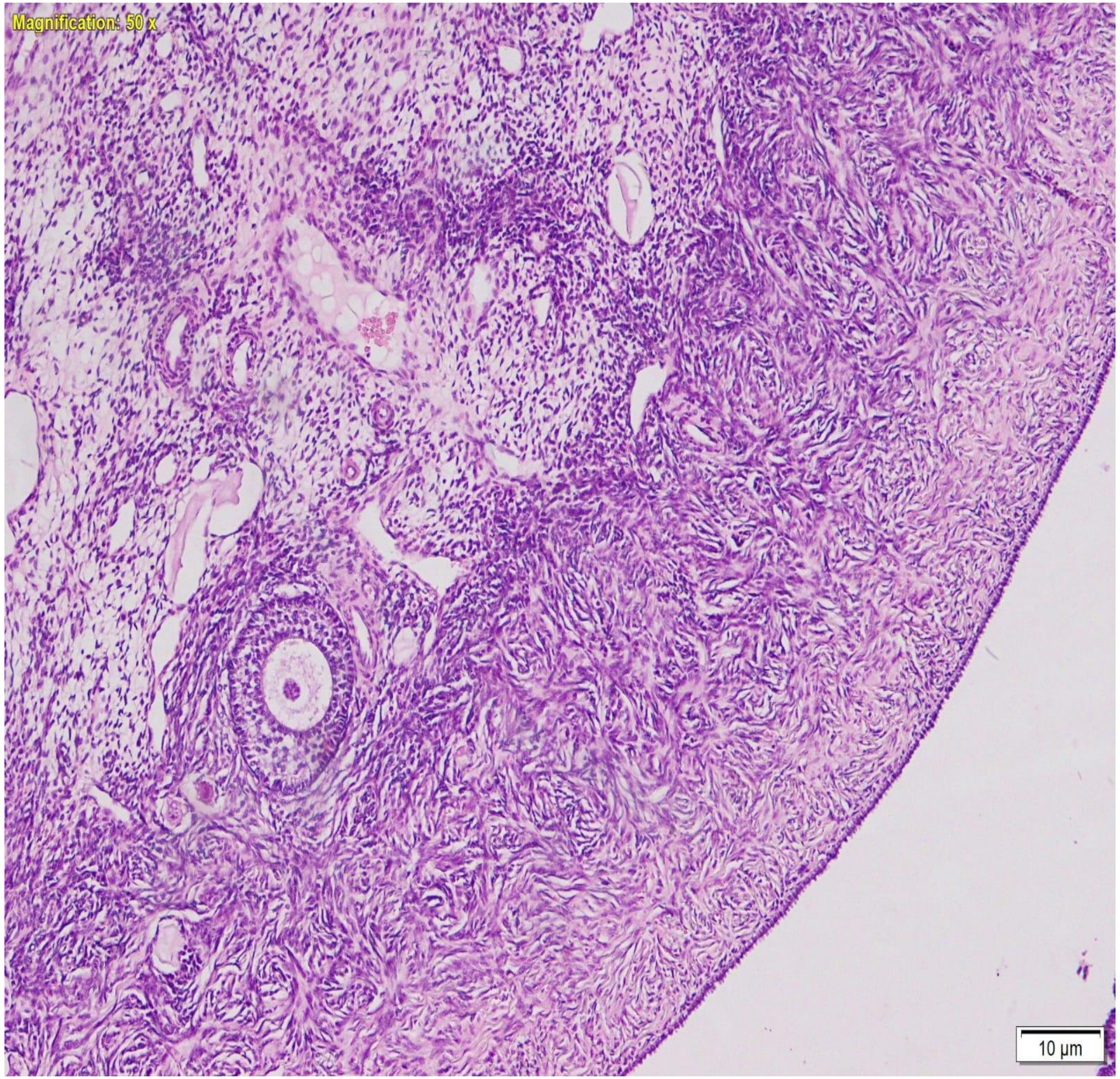

This is a histology slide of the ovarium.
What are the structures that can be observed from the image?

BOT-1

This histological image of the ovary appears to show the following structures:

1. **Primordial Follicles**: These are small, round structures located near the cortex of the ovary. They are characterized by a single layer of flattened granulosa cells surrounding an oocyte.
2. **Developing (Primary) Follicles**: These have a larger oocyte surrounded by a layer of cuboidal granulosa cells. The follicle's basement membrane is also evident.
3. **Stromal Tissue**: The dense connective tissue with fibroblast-like cells seen between the follicles provides structural support to the ovary.
4. **Blood Vessels**: Larger clear spaces lined by endothelial cells may represent blood vessels within the stromal tissue.
5. **Cortical Region**: The outer portion of the ovary, which is more cellular and contains the majority of the primordial follicles.
6. **Medullary Region**: The inner portion of the ovary (less cellular) where blood vessels, lymphatics, and nerves are commonly observed.
7. **Tunica Albuginea**: A thin, fibrous layer located just beneath the surface epithelium, which may be visible as a dense connective tissue layer.

If you'd like more detailed annotations or clarifications, let me know!

BOT-2

This histological image of an ovary shows several key structures:

- **Primordial Follicles:** These are the most numerous and appear as small, darkly stained oocytes surrounded by a single layer of flattened granulosa cells. They represent the earliest stage of follicle development.
- **Primary Follicles:** These follicles are characterized by a larger oocyte surrounded by a single layer of cuboidal granulosa cells.[1]
- **Secondary Follicles:** These are identifiable by the presence of multiple layers of granulosa cells surrounding the oocyte and the early formation of a fluid-filled space called the antrum.[2]
- **Oocyte:** This is the female germ cell and is seen as a large, round structure within the follicles.[3] It contains a prominent nucleus and is surrounded by the zona pellucida, a clear layer.[4]
- **Granulosa Cells:** These cells form layers around the oocyte and are responsible for nourishing the oocyte and producing hormones like estrogen.[5]
- **Theca Cells:** These cells surround the granulosa cells and contribute to hormone production.[6]
- **Stroma:** This is the connective tissue framework of the ovary and supports the follicles and blood vessels.

It's important to note that the specific types and stages of follicles present can vary depending on the age and hormonal status of the individual from whom the sample was taken.

BOT-3

This histological image of the ovary, magnified 50 times, shows several distinct structures:

1. **Follicles**: Small spherical structures within the ovary, containing an oocyte (egg cell) surrounded by granulosa cells.
2. **Stroma**: The fibrous and dense background tissue, providing structural support and containing connective tissue and blood vessels.
3. **Blood Vessels**: Visible as small, round, or elongated structures with clear lumens.
4. **Surface Epithelium**: The outermost layer of the ovary, seen on the right side of the image.
5. **Cortex and Medulla**: The ovarian cortex, containing the follicles, and the medulla, centrally located with blood vessels and connective tissue.

This detailed view of ovarian tissue is crucial for understanding reproductive biology and the development of oocytes, highlighting the essential structures for hormone production and egg maturation.

Palm Skin

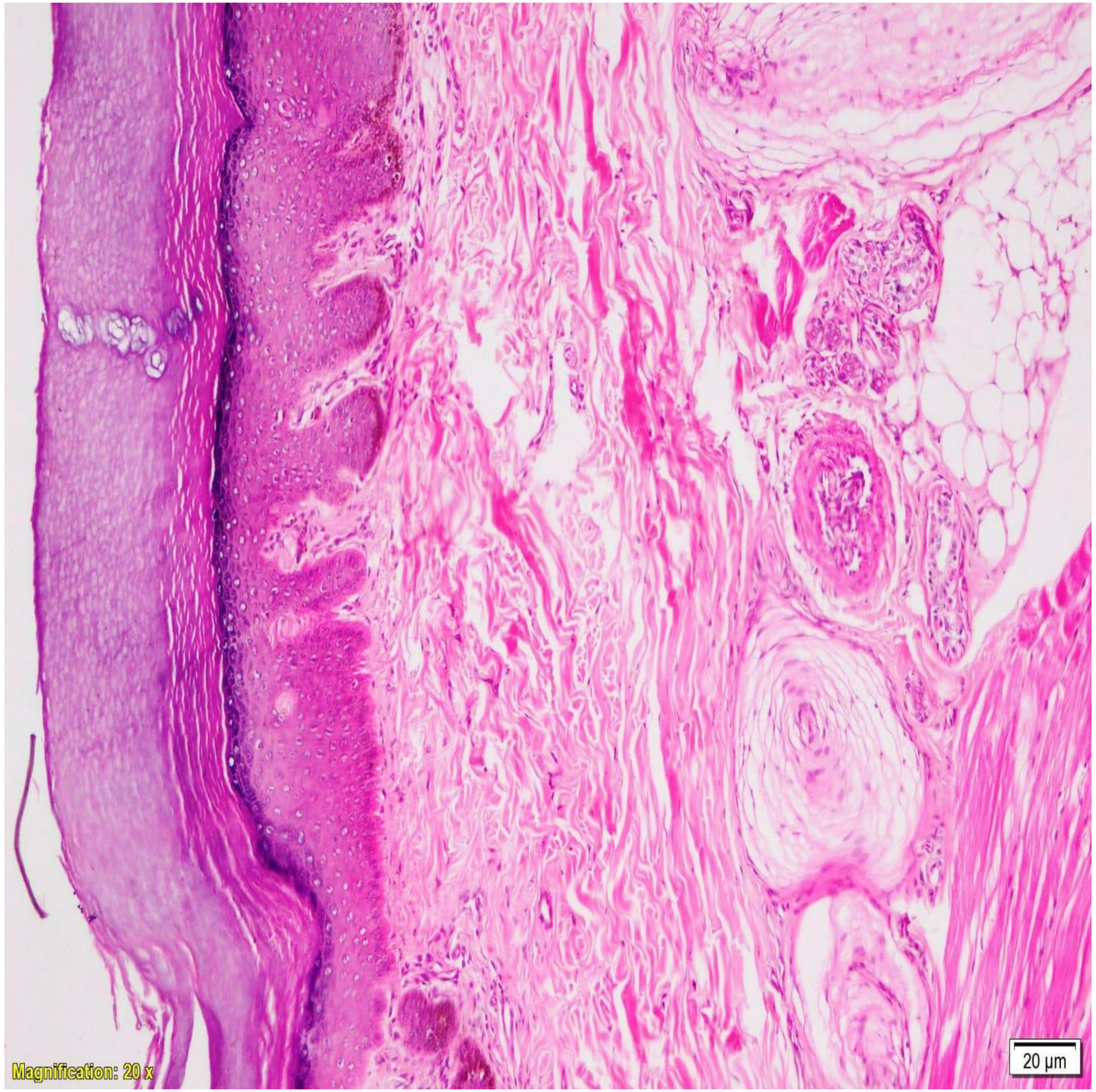

This is a histology slide of the palm skin.
What are the structures that can be observed from the image?

BOT-1

This histological preparation of the palm skin (thick skin) stained with H&E highlights several key structures that can be observed:

1. **Epidermis** (left side of the image):
   - The outermost layer is the **stratum corneum**, consisting of keratinized cells without nuclei.
   - Beneath that, layers of the epidermis can be seen, including the **stratum granulosum**, **stratum spinosum**, and **stratum basale**.
2. **Dermis** (central region):
   - The connective tissue beneath the epidermis. It contains:
     - **Collagen fibers** (wavy, eosinophilic pink areas).
     - **Sweat gland ducts** (small circular or tubular structures in cross-section, lined by epithelial cells).
     - **Pacinian corpuscles** (larger, round, laminated structures indicative of mechanoreceptors, visible in the dermis).
3. **Hypodermis or Subcutaneous tissue** (right side):
   - Adipose tissue is visible (white vacuolated areas).
   - Some small blood vessels and connective tissue are present.

The image highlights the characteristic features of thick skin, including the thickened stratum corneum and absence of hair follicles.

BOT-2

This histological image of palm skin shows a number of key structures. Here's a breakdown:

Epidermis:

- **Stratum Corneum:** This is the outermost layer of the epidermis, composed of dead, keratinized cells.[1] It appears as a thick, pink band at the top of the image.
- **Stratum Granulosum:** Located beneath the stratum corneum, this layer is characterized by cells containing granules.[2] It's thinner and may not be clearly distinguishable in this specific image.
- **Stratum Spinosum:** This layer is composed of cells with spiny projections. It's located below the stratum granulosum and is also difficult to discern in this image.
- **Stratum Basale:** The deepest layer of the epidermis, where new skin cells are produced.[3] It's a single layer of cuboidal cells resting on the basement membrane, visible as the deepest layer of the epidermis.[4]

Dermis:

- **Papillary Dermis:** This is the upper layer of the dermis, characterized by dermal papillae that interdigitate with the epidermis.[5] It appears as a lighter pink region with a wavy border below the epidermis.
- **Reticular Dermis:** This is the deeper layer of the dermis, composed of dense irregular connective tissue.[6] It contains blood vessels, sweat glands, and hair follicles (although hair follicles are absent in palm skin). It appears as a darker pink region with thicker collagen fibers.

Other Structures:

- **Sweat Glands:** These are coiled glands found in the dermis.[7] Their ducts can be seen traversing the epidermis to open on the skin surface. They appear as circular or oval structures with a clear lumen.
- **Blood Vessels:** Small blood vessels can be seen throughout the dermis, appearing as thin-walled structures containing red blood cells.
- **Collagen Fibers:** These are the main structural component of the dermis, appearing as pink, wavy bundles.[8]
- **Nerves:** While not easily visible in this image, nerves are present throughout the dermis.
- **Important Note:** This image is specifically of palm skin, which is thicker than skin in other areas of the body. It lacks hair follicles and sebaceous glands, which are present in thin skin.[9]

BOT-3

From this histological preparation of the palm skin, several distinct structures can be observed:

1. Epidermis: The outermost layer, appearing as a thick, dark purple band. This layer is composed of stratified squamous epithelium.
2. Dermis: Located beneath the epidermis, containing a mix of dense irregular connective tissue, collagen fibers, and various cellular components. It appears lighter and more fibrous.
3. Sweat Glands: Circular structures within the dermis, identifiable by their coiled appearance and darker staining.
4. Hair Follicles: Typically absent in palm skin, so their absence is noteworthy.
5. Adipose Tissue: The light, bubble-like structures, indicating the presence of fat cells.
6. Dermal Papillae: Wavy projections at the interface between the epidermis and dermis, increasing the surface area for nutrient exchange.

These structures highlight the complex anatomy of the skin, especially the thickened epidermis and specialized structures like sweat glands, which are crucial for the unique functions of palm skin, such as grip and sweat production.

Ileum

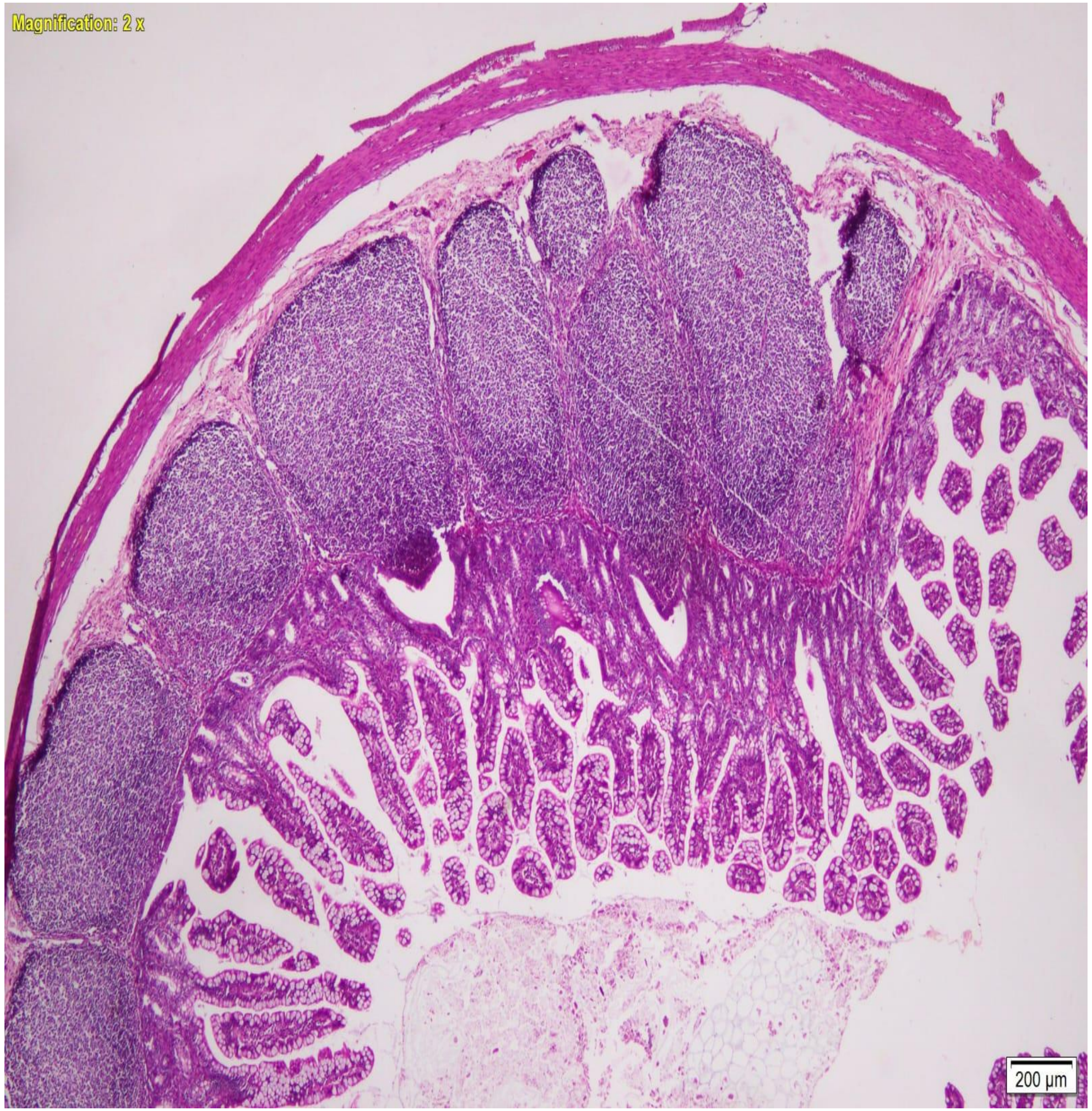

This is a histology slide of the ileum.
What are the structures that can be observed from the image?

BOT-1

This is a histological section of the ileum stained with hematoxylin and eosin (H&E). Several characteristic structures of the ileum can be observed:

1. **Mucosa**:
    - **Villi**: Finger-like projections of the mucosa extending into the lumen. These increase the surface area for nutrient absorption.
    - **Crypts of Lieberkühn**: Glandular structures located at the base of the villi. These are part of the intestinal glands.
2. **Submucosa**:
    - **Peyer's patches**: Large lymphoid aggregates visible as densely packed purple-staining regions. These are part of the gut-associated lymphoid tissue (GALT) and play a role in immune responses.
3. **Muscularis propria (externa)**:
    - This layer consists of smooth muscle arranged in two layers: an inner circular layer and an outer longitudinal layer, which facilitate peristalsis.
4. **Serosa**:
    - The outermost layer of connective tissue, covered by mesothelium.

Would you like more detailed labeling or any further analysis?

BOT-2

This is a great image showcasing the ileum, the final section of the small intestine. Here's a breakdown of the visible structures:

Mucosa: This is the innermost layer, and it's highly folded to increase surface area for absorption.[1]

- **Villi:** These are finger-like projections of the mucosa that extend into the lumen (the open space within the ileum).[2] They're covered in epithelial cells and contain blood vessels and lacteals (lymphatic vessels) for nutrient absorption.[3] In this image, you can see the villi cut in various orientations – some longitudinally and others in cross-section.
- **Crypts of Lieberkühn:** These are the intestinal glands found between the villi.[4] They extend down into the mucosa and contain various cell types, including stem cells, goblet cells (which secrete mucus), and enteroendocrine cells (which produce hormones).
- **Epithelium:** This is the layer of cells lining the villi and crypts.[5] It's primarily composed of enterocytes (absorptive cells) with a brush border (microvilli) to further increase surface area.[6] You can also see goblet cells interspersed among the enterocytes.[7]
- **Lamina Propria:** This is the connective tissue layer within the mucosa.[8] It supports the epithelium and contains blood vessels, nerves, and immune cells.[9]
- **Muscularis Mucosae:** This is a thin layer of smooth muscle that separates the mucosa from the submucosa.[10]

Submucosa: This is the layer of connective tissue beneath the mucosa.[11]

- **Blood vessels:** Larger blood vessels can be seen in the submucosa, branching to supply the mucosa.[12]
- **Lymphoid follicles:** These are collections of immune cells that help protect against pathogens.[13] In the ileum, they often form larger aggregates called Peyer's patches, which may be visible in this image.[14]

Muscularis Externa: This is the layer responsible for the motility of the ileum.

- **Inner circular layer:** This layer of smooth muscle constricts the lumen.
- **Outer longitudinal layer:** This layer of smooth muscle shortens and widens the lumen.

Serosa: This is the outermost layer, a thin layer of connective tissue that covers the ileum.[15] It may not be clearly visible in this particular image.

It's worth noting that the ileum has some distinctive features compared to other parts of the small intestine:

- **Shorter villi:** The villi in the ileum are shorter and broader than those in the duodenum and jejunum.[16]
- **More goblet cells:** The ileum has a higher density of goblet cells, reflecting its role in mucus production.
- **Prominent Peyer's patches:** These lymphoid aggregates are more numerous in the ileum.[17]

BOT-3

The histological preparation of the ileum displays several important structures:

1. **Villi**: Finger-like projections extending into the lumen, which increase the surface area for absorption.
2. **Crypts of Lieberkühn**: Glandular structures at the base of the villi, involved in secretion.
3. **Lymphoid Nodules (Peyer's Patches)**: Aggregates of lymphoid tissue, part of the immune system, visible as dense purple areas.
4. **Muscularis Mucosae**: A thin muscle layer found beneath the mucosa.
5. **Submucosa**: Connective tissue layer beneath the mucosa, containing blood vessels, nerves, and lymphatics.
6. **Muscularis Externa**: Layers of smooth muscle responsible for peristalsis.
7. **Serosa**: The outermost layer, consisting of thin connective tissue.

These structures are crucial for the ileum's function in nutrient absorption and immune response. The preparation shows a cross-section stained with hematoxylin and eosin (H&E), providing a clear view of these distinct layers and cells.

Cervix Cancer, Small Cell carcinoma

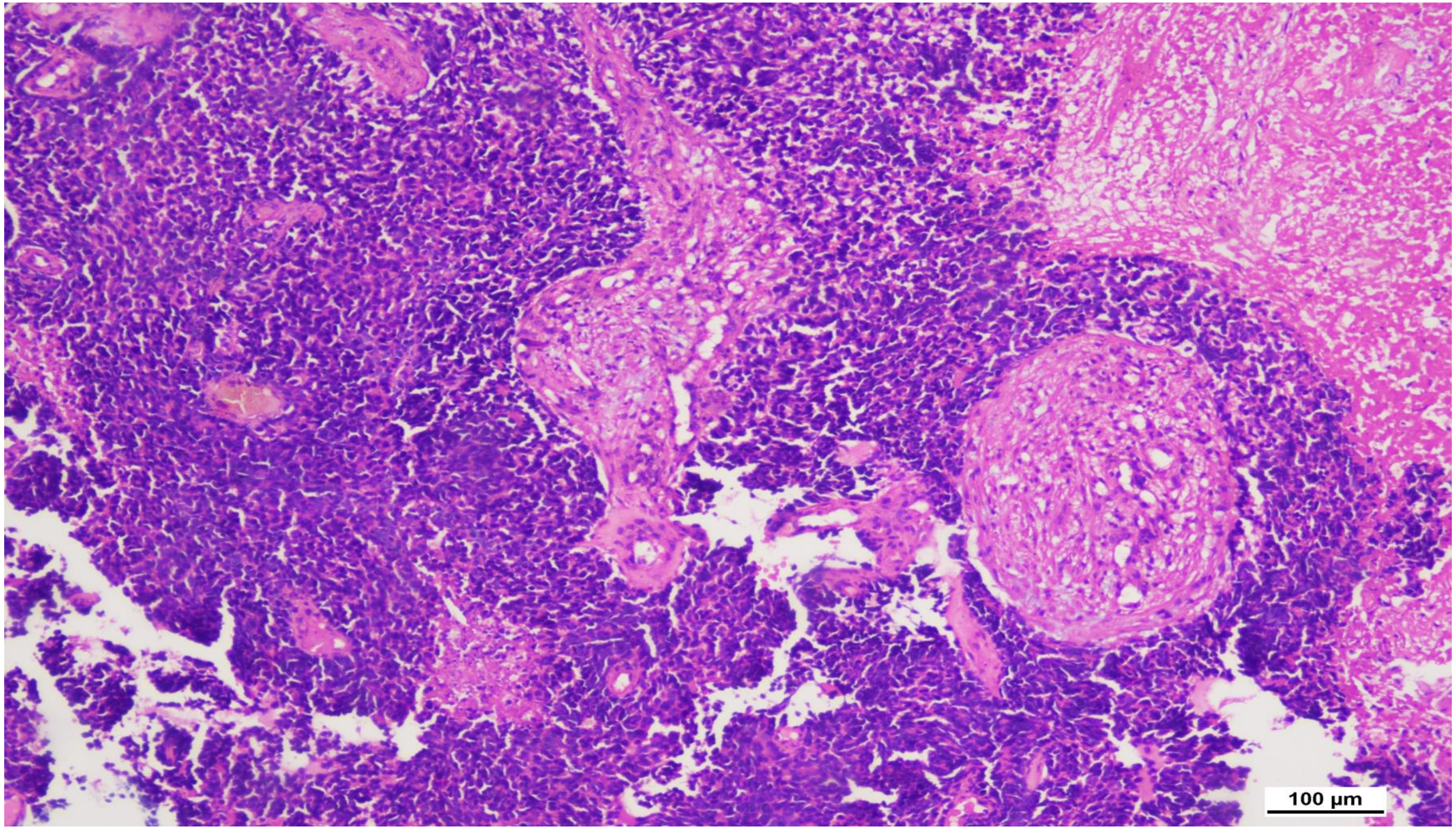

This is a histopathology image of a 55-year-old woman who was diagnosed with cervical cancer. She underwent radical hysterectomy and pelvic lymph node dissection surgery.

Based on this cervix sample, what is the interpretation of the image?

10

BOT-1

This histopathology image shows a high-power view of a cervical cancer specimen. Based on the context provided and the features visible in the image, the findings suggest the following interpretation:

1. **Tumor Characteristics**:
   - The image shows densely packed small cells with hyperchromatic nuclei and scant cytoplasm, which are hallmarks of **small cell carcinoma** of the cervix.
   - The nuclear molding, frequent mitotic figures, and areas of necrosis are typical features of this high-grade malignancy.
2. **Surrounding Tissue**:
   - The desmoplastic stromal reaction visible in the surrounding tissue indicates the tumor's invasive nature.
   - Lymphovascular space invasion (LVSI) is a possibility, although confirmation requires specific markers or immunohistochemistry.
3. **Differential Diagnosis**:
   - The appearance is consistent with **small cell neuroendocrine carcinoma** of the cervix.
   - Immunohistochemistry for neuroendocrine markers such as synaptophysin, chromogranin A, and CD56 would help confirm the diagnosis.
4. **Surgical and Prognostic Relevance**:
   - Radical hysterectomy and pelvic lymph node dissection aim to remove the primary tumor and assess the extent of metastasis, particularly in the lymph nodes.
   - The aggressive behavior of small cell carcinoma often warrants multimodal therapy, including chemotherapy and radiation, due to its high risk of recurrence and metastasis.

If further clarification or specific marker analysis is needed, immunohistochemical stains or additional clinical context (e.g., lymph node status, metastases) would provide more definitive diagnostic and prognostic insights.

BOT-2

Based on the information and the image provided, this histopathology slide likely shows **metastatic squamous cell carcinoma in a lymph node**. Here's why:

- **Patient History:** The 55-year-old woman has cervical cancer and underwent lymph node dissection. This suggests the image is likely from one of the removed lymph nodes, and we're looking for evidence of metastasis (spread of cancer).
- **Image Features:**
  - **Lymph node architecture:** You can see the typical structure of a lymph node on the right side of the image. This includes a more organized arrangement of cells, likely lymphocytes within the node.
  - **Tumor cells:** The left side of the image shows a disorganized mass of cells with features characteristic of squamous cell carcinoma. These include:
    - **Nests and sheets of cells:** The cancerous cells are arranged in distinct groups.
    - **Cellular atypia:** The cells show variation in size and shape (pleomorphism), with enlarged, irregular nuclei and a high nuclear-to-cytoplasmic ratio.
    - **Keratinization:** You might see areas of pink keratinization within the tumor nests, a hallmark of squamous cell carcinoma.

In summary: This image likely demonstrates that the cervical squamous cell carcinoma has spread (metastasized) to the lymph nodes. This is important prognostic information that helps stage the cancer and guide further treatment decisions.

Important Notes:

- **Definitive diagnosis:** A pathologist would provide a definitive diagnosis by examining the slide under a microscope and potentially performing additional tests (e.g., immunohistochemistry).
- **Further information:** More clinical information (like the original cervical cancer grade) would help in a more complete assessment.
- **Treatment implications:** The presence of lymph node metastasis usually indicates a more advanced stage of cancer, which may require more aggressive treatment like radiation therapy or chemotherapy in addition to surgery.

BOT-3

This histopathology image, stained with hematoxylin and eosin (H&E), reveals a section of cervical tissue. Here are some key observations from the image:

1. Irregular Cell Shapes: The cells exhibit significant variability in size and shape, a hallmark of malignancy. The nuclei appear large and hyperchromatic (darkly stained), indicating high nuclear-to-cytoplasmic ratios, which is typical in cancerous cells.
2. Dense Cell Aggregation: There is a dense cluster of cells, suggesting the presence of a tumor mass. These cells are closely packed, further supporting the diagnosis of cancer.
3. Necrosis: Some areas show cell death (necrosis), which is common in tumors due to rapid cell growth outpacing blood supply.
4. Fibrosis: The presence of fibrous tissue indicates a reaction to the tumor, often seen in chronic conditions and malignancies.
5. Inflammatory Cells: There might be the presence of inflammatory cells surrounding the tumor, as the body attempts to respond to the cancerous growth.

These features confirm the diagnosis of cervical cancer and provide insights into the pathological changes occurring within the tissue. The presence of necrosis and fibrosis, along with the abnormal cellular morphology, aligns with typical findings in malignant tissues. This histopathological examination is crucial for understanding the extent and nature of the cancer, guiding further treatment and management decisions.

Ovarian Cancer, Clear Cell carcinoma

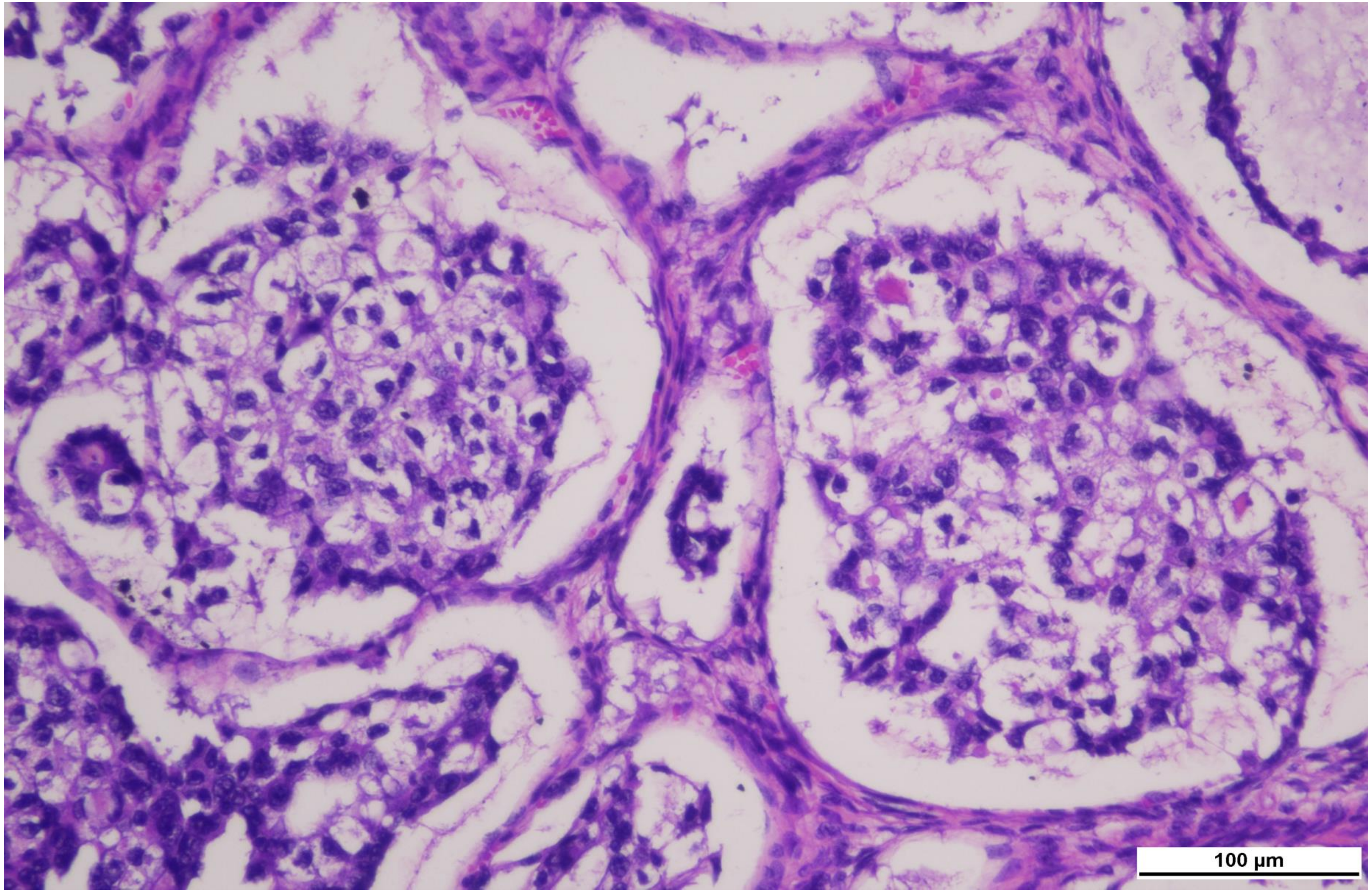

This is a histopathology image of a 63-year-old woman who was diagnosed with ovarian cancer. She underwent a hysterectomy and bilateral salpingo-oophorectomy.

Based on this ovarian sample, what is the interpretation of the image?

BOT-1

This histopathology image is a high-power view of an ovarian cancer specimen. Based on the morphological features and clinical context provided, the interpretation is as follows:

Key Observations:

1.Cellular Architecture:

- •The image shows glandular or tubulocystic structures with cells lining the luminal spaces.
- •The lining cells have clear cytoplasm, distinct cell borders, and centrally or eccentrically placed nuclei. This is characteristic of **clear cell carcinoma** of the ovary.

2.Nuclear Features:

- •The nuclei appear atypical, with some demonstrating prominent nucleoli, a common feature in clear cell carcinoma.
- •The nuclear-to-cytoplasmic ratio is relatively low due to the abundant cytoplasm.

3.Cytoplasmic Features:

- •The clear cytoplasm is due to the presence of glycogen, which is often seen in clear cell tumors. Periodic acid-Schiff (PAS) staining with diastase digestion can confirm this finding.

4.Stromal Features:

- •The surrounding stroma appears fibrotic with areas of desmoplastic reaction, consistent with an invasive malignancy.

Diagnosis:

•The findings are consistent with **clear cell carcinoma of the ovary**, a subtype of epithelial ovarian cancer. It is known for its association with endometriosis and a relatively aggressive clinical behavior.

Clinical Relevance:

1.Prognosis:

- •Clear cell carcinoma of the ovary is associated with poor prognosis compared to other epithelial ovarian cancers like serous carcinoma.
- •It is typically resistant to platinum-based chemotherapy.

2.Further Analysis:

- •Immunohistochemistry may reveal markers such as Napsin A, HNF-1β (positive), and WT1 (negative) to support the diagnosis.
- •Assessing for vascular or lymphatic invasion in adjacent sections can provide additional prognostic information.

3.Management Implications:

- •Surgery followed by chemotherapy is the standard treatment. However, due to its resistance to conventional chemotherapy, targeted therapies may be considered in the future.

If further clarification or immunohistochemical correlation is needed, additional sections or clinical data could provide further insights.

BOT-2

This image likely shows a **high-grade serous ovarian carcinoma**. Here's a breakdown of the evidence:

- **Patient History:** A 63-year-old woman with ovarian cancer who underwent a hysterectomy and bilateral salpingo-oophorectomy strongly suggests this image is from her ovarian tissue.
- **Image Features:**
  - **Complex glandular structures:** The image shows irregular, crowded glands lined by atypical cells. This is a common pattern seen in serous carcinomas.
  - **Significant cellular atypia:** The cells lining the glands show marked variation in size and shape (pleomorphism), with enlarged, hyperchromatic (darkly staining) nuclei and prominent nucleoli. This indicates a high-grade tumor.
  - **High nuclear-to-cytoplasmic ratio:** The cells have relatively little cytoplasm compared to the size of their nuclei, another feature of malignancy.
  - **Mitotic figures:** You might see cells undergoing division (mitosis), which is a sign of rapid proliferation often seen in aggressive cancers.
  - **Possible papillary structures:** Some areas might show finger-like projections (papillae) lined by the atypical cells, a characteristic feature of serous carcinomas.

Why high-grade serous carcinoma?

- **Most common type:** High-grade serous carcinoma is the most common type of ovarian cancer.
- **Microscopic appearance:** The features described above align well with the typical microscopic appearance of high-grade serous carcinoma.

Important Notes:

- **Definitive diagnosis:** A pathologist would make the final diagnosis by examining the slide under a microscope, considering the patient's clinical history, and potentially performing additional tests (e.g., immunohistochemistry).
- **Grading and staging:** The pathologist would also assess the tumor grade (degree of cellular atypia) and stage (extent of spread) to guide treatment decisions.
- **Treatment implications:** High-grade serous carcinoma is often aggressive, so this diagnosis typically leads to further treatment like chemotherapy.

This interpretation provides a likely explanation for the image based on the available information. Always remember that a definitive diagnosis requires a comprehensive evaluation by a qualified pathologist.

BOT-3

This histopathology image, stained with hematoxylin and eosin (H&E), shows several key features indicative of ovarian cancer:

1. **Cellular Architecture**: There are clusters of cells with high nuclear-to-cytoplasmic ratios, which is a common characteristic of malignant cells. The nuclei are densely stained (dark purple), indicating hyperchromasia.
2. **Structural Disorganization**: The normal architecture of ovarian tissue is disrupted. There is a lack of organized glandular or stromal structures, which is typical in malignant tissues.
3. **Mitotic Figures**: Numerous cells are undergoing mitosis, indicating rapid cell division—a hallmark of cancer.

These histopathological features confirm the diagnosis of ovarian cancer and highlight the aggressive nature of the disease due to the high proliferation rate and disrupted tissue architecture. This examination is crucial for understanding the extent and nature of the cancer, guiding further treatment and management decisions.

Ovarian Cancer, Mucinous type

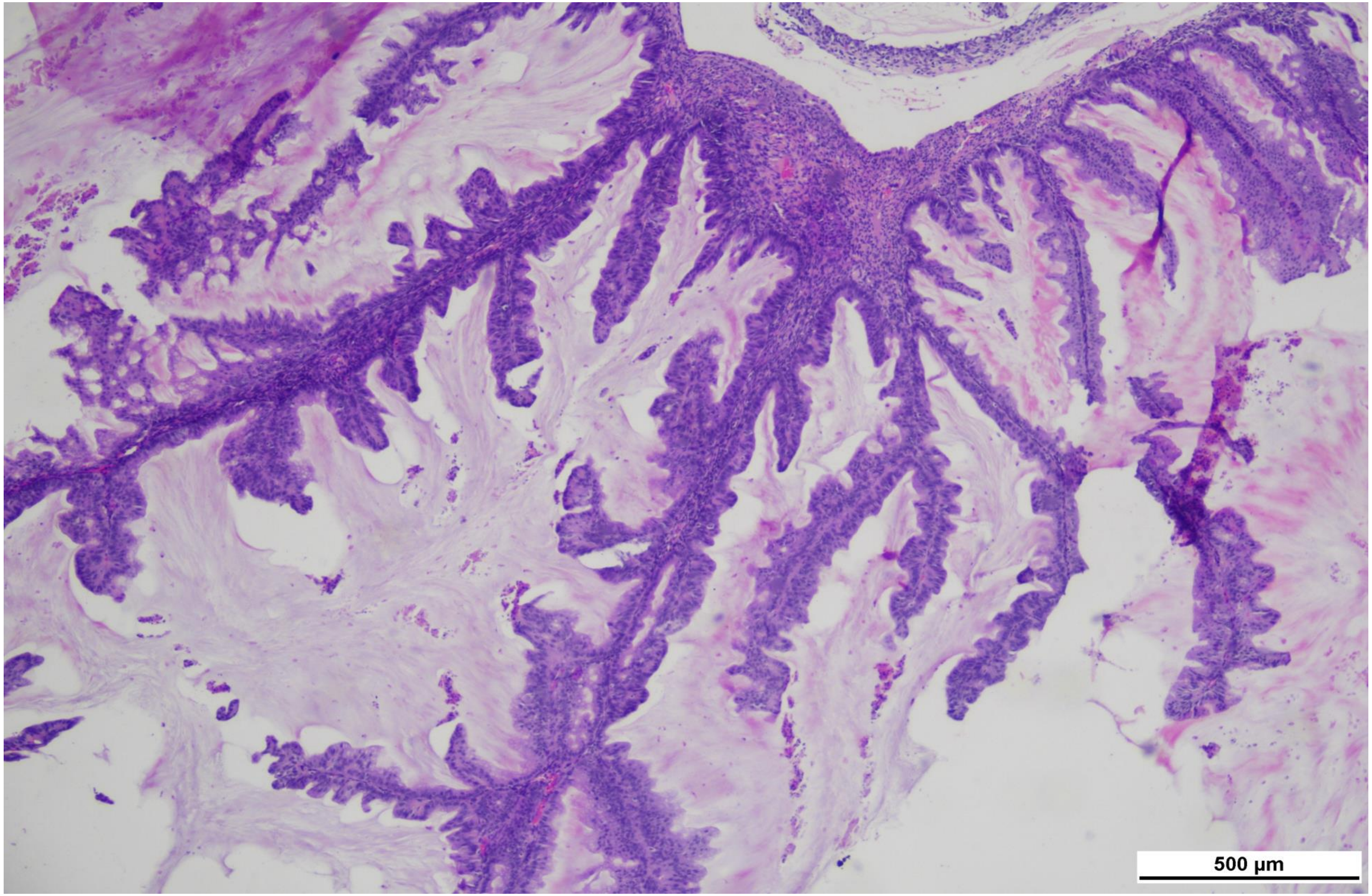

This is a histopathology image of a 59-year-old woman who was diagnosed with ovarian cancer. She underwent a hysterectomy and bilateral salpingo-oophorectomy.

Based on this ovarian sample, what is the interpretation of the image?

12

BOT-1

This histopathology image demonstrates a low-power view of an ovarian tumor with distinctive morphological features. Based on the context provided and visible details, the interpretation is as follows:

Key Observations:

1. **Architectural Pattern:**
    - The image shows a papillary and expansile growth pattern with prominent epithelial-lined structures.
    - The papillae are covered by columnar epithelial cells with abundant cytoplasm, and the intervening stroma is fibrotic.
2. **Cellular Features:**
    - The epithelial lining appears tall, columnar, and mucin-rich, suggesting a **mucinous neoplasm**.
    - Nuclear atypia is present but not overtly severe in this section, consistent with a mucinous carcinoma rather than a borderline tumor.
3. **Stromal Changes:**
    - The stroma is fibrotic and acellular in some areas, which is commonly seen in mucinous tumors with an expansile pattern of invasion.
4. **Differential Diagnosis:**
    - The findings are consistent with **mucinous carcinoma of the ovary**, characterized by its expansile growth pattern and mucin-producing epithelium.
    - Differential diagnoses include borderline mucinous tumors or metastatic mucinous adenocarcinoma (e.g., from the gastrointestinal tract), but the expansile growth and the lack of infiltrative glands favor a primary ovarian mucinous carcinoma. .

Diagnosis:

- **Primary mucinous carcinoma of the ovary (expansile type).**

Clinical Relevance:

1. **Prognostic Implications:**
    - Mucinous carcinomas of the ovary are rare and generally carry a better prognosis than high-grade serous carcinomas if confined to the ovary.
    - The expansile pattern, as opposed to the infiltrative pattern, is associated with a more favorable outcome.
2. **Further Analysis:**
    - Immunohistochemistry (e.g., CK7-positive, CK20-negative, CDX2-negative) can help confirm the primary ovarian origin.
    - Ruling out metastatic disease (especially from gastrointestinal primaries) is essential.
3. **Treatment and Management:**
    - Surgery is the mainstay of treatment, often followed by chemotherapy if the tumor is advanced or shows features of aggressive behavior.

This interpretation should be correlated with the clinical findings, radiological data, and any available immunohistochemical studies to provide a comprehensive diagnosis

BOT-2

This image most likely shows a **borderline serous ovarian tumor**, also known as a **low malignant potential (LMP) serous tumor**. Here's why:

•**Patient History:** A 59-year-old woman with ovarian cancer who underwent surgery points towards an ovarian tumor.

•**Image Features:**

- •**Branching papillae:** The image displays numerous, complex, finger-like projections (papillae) extending into cystic spaces. This is a hallmark of serous tumors.
- •**Mild to moderate atypia:** The cells covering the papillae show some degree of nuclear enlargement and irregularity, but not to the extent seen in high-grade serous carcinoma. This suggests a borderline tumor.
- •**Stratification:** You might notice some areas where the cells are layered (stratified) on the papillae, another feature often seen in borderline tumors.
- •**Lack of destructive invasion:** Importantly, there's no clear evidence of invasion into the surrounding stroma (connective tissue), which would be expected in a frankly malignant tumor.

Why borderline serous tumor?

•**Common in this age group:** Borderline serous tumors are more common in premenopausal and perimenopausal women.

•**Microscopic features:** The described features align with the characteristic appearance of borderline serous tumors.

Important Notes:

•**Definitive diagnosis:** A pathologist makes the final diagnosis by examining the slide under a microscope, considering the patient's clinical history, and potentially performing additional tests (e.g., immunohistochemistry).

•**Prognosis:** Borderline tumors generally have a better prognosis than invasive carcinomas. They tend to grow slowly and rarely spread. However, careful follow-up is necessary as a small percentage can recur or progress to invasive cancer.

•**Treatment implications:** The treatment for borderline tumors typically involves surgery. Further treatment may depend on factors like the extent of the tumor and the patient's overall health.

This interpretation offers a likely explanation for the image based on the provided information. Remember that a definitive diagnosis and treatment plan require a comprehensive evaluation by a healthcare professional.

BOT-3

This histopathology image reveals the following characteristics, crucial for diagnosing ovarian cancer:

1. **Complex Glandular Structures**: These are indicative of serous carcinoma, a common type of ovarian cancer. The glandular patterns show papillary formations, which are typical in this type of malignancy.
2. **High Cellular Density**: The tissue shows densely packed cells, a hallmark of cancerous tissue.
3. **Hyperchromatic Nuclei**: The dark purple staining indicates hyperchromasia, where the nuclei are abnormally dark due to increased DNA content, suggesting malignancy.

These features collectively confirm the diagnosis of ovarian cancer and highlight the aggressive nature of the disease. The presence of complex glandular structures with papillary formations is particularly indicative of serous carcinoma, guiding further treatment and management decisions.
